# Challenges in selecting admixture models and marker sets to infer genetic ancestry in a Brazilian admixed population

**DOI:** 10.1038/s41598-022-25521-7

**Published:** 2022-12-08

**Authors:** Luciana Maia Escher, Michel S. Naslavsky, Marília O. Scliar, Yeda A. O. Duarte, Mayana Zatz, Kelly Nunes, Silviene F. Oliveira

**Affiliations:** 1 Human Genetics Laboratory, Institute of Biological Sciences, University of Brasilia, Brasília, DF Brazil; 2 Department of Genetics and Evolutionary Biology, Biosciences Institute, University of São Paulo, São Paulo, SP Brazil; 3 Human Genome and Stem Cell Research Center, University of São Paulo, São Paulo, SP Brazil; 4 Medical-Surgical Nursing Department, School of Nursing, University of São Paulo, São Paulo, SP Brazil; 5 Epidemiology Department, Public Health School, University of São Paulo, São Paulo, SP Brazil

**Keywords:** Genetic variation, Genetic markers, Genetics

## Abstract

The inference of genetic ancestry plays an increasingly prominent role in clinical, population, and forensic genetics studies. Several genotyping strategies and analytical methodologies have been developed over the last few decades to assign individuals to specific biogeographic regions. However, despite these efforts, ancestry inference in populations with a recent history of admixture, such as those in Brazil, remains a challenge. In admixed populations, proportion and components of genetic ancestry vary on different levels: (i) between populations; (ii) between individuals of the same population, and (iii) throughout the individual's genome. The present study evaluated 1171 admixed Brazilian samples to compare the genetic ancestry inferred by tri-/tetra-hybrid admixture models and evaluated different marker sets from those with small numbers of ancestry informative markers panels (AIMs), to high-density SNPs (HDSNP) and whole-genome-sequence (WGS) data. Analyses revealed greater variation in the correlation coefficient of ancestry components within and between admixed populations, especially for minority ancestral components. We also observed positive correlation between the number of markers in the AIMs panel and HDSNP/WGS. Furthermore, the greater the number of markers, the more accurate the tri-/tetra-hybrid admixture models.

## Introduction

Understanding how human genetic diversity is distributed and its implications has been a recurrent focus in clinical, population, and forensic genetics studies^1–3^. Since the 1970s, owing to the pioneering work by Richard Lewontin, it has been understood that most human genetic variation occurs between individuals of the same population group, while genetic variation between individuals of distant populations is restricted to a small proportion of the human genome. This study is one of the first to refute the use of social races in biological studies and draw attention to the fact that genetic information is more accurate for biological issues than social groups or ethno-racial self-declaration^4^.

Since then, several studies have subsequently confirmed these observations and revealed that the distribution of genetic diversity and population differentiation is a continuous gradient within and between populations across continents^5,6^. Therefore, the categorization of human groups by current geo-political regions are arbitrary choices and not true biological clusters. Thus, these studies clarify, that there is only a small set of genetic polymorphisms with a distinct allelic frequency between human populations or continents.

As such, these small genetic differences have been widely studied to address specific biological issues. For example, in clinical genetics, some diseases are recognized as having different incidences among population groups, for example: chronic kidney disease^7^, hypertension^8^, and inflammatory bowel disease^9^. Identifying associated genetic variants and the correct assignment of individuals in these groups helps in the development of personalized medical care^10^. In forensic genetics, when identifying the individual via CODIS (the Combined DNA Index System), it is often necessary to provide additional information such as phenotypic characteristics (eye, hair, and skin color) and/or the most probable continental origin^11^.

Over the last few decades, several methods and strategies have been developed in an attempt to assign an individual´s geographical origin based on DNA variations. This is what geneticists often refer to as genetic ancestry^12^. One of the first strategic approaches applied was to identify sets with a few dozen ancestry informative markers (AIMs—genetic markers which exhibit substantially different frequencies between different populations) in order to compose informative ancestry panels with the purpose of clustering individuals into continental and subcontinental population groups^13–15^. The development of AIM panels attempts to select a group of genetic markers that compose a small, accurate, low-cost, and highly informative set. In general, AIM panels vary in some characteristics, including: specific loci, number of loci, genotyping strategies, and parental reference populations^16–18^. Studies usually use only one AIM panel or even a complementary set, for example: PIMA + 34-plex^19^, Pacifiplex and 34-plex^20^, and KiddLab + Seldin + 34-plex^21^.

On the other hand, the emergence of high-throughput genotyping technologies, such as high-density SNPs array (HDSNPs), whole-exome-sequence (WES) and whole-genome-sequence (WGS) enabled high horizontal genome coverage studies for genomic ancestry inference^22,23^. In this scenario, there is not only a significant increase in the number of genetic markers evaluated, the number of genotyped individuals per study grows from hundreds to thousands^24–26^. Consequently, some analytical approaches have been adapted, in addition to the development of new analytical strategies^27–29^.

Additionally, in both the AIM and high-throughput genotyping strategies, proper genetic ancestry inference is dependent on the existence of a reference population panel for each ancestral component under study. Genetic ancestry inference remains deficient in some population subgroups due to lack of reference data collection, in addition to some inconclusive or non-validated studies. For example, there is an effort to develop reference panels for Asians and Native Americans, in addition to increasing reference genome data for Latin Americans^30^.

Admixed populations, such as those in America, pose a peculiar case for genetic ancestry inference as, they originated over the last 500 years through a complex admixture process with population sources of individuals from different continents: Native Americans, Europeans, Africans^31,32^ and more recently East Asians^33^. The genomes of admixed individuals are a redistribution of genetic variation observed in parental populations, which produces new genomic combinations of pre-existing genetic variants. This leads to a paradigm shift, in which the geographic origin of the admixed individual becomes a secondary issue, the primary objective being to identify each ancestral component, its distribution and proportion in the individual's admixed genome.

Furthermore, in admixed populations, the proportion and components of genetic ancestry vary at different levels: (i) between populations; (ii) between individuals of the same population, and (iii) throughout the individual's genome^34^. This has a direct impact on the reproducibility and transposition of study results in these populations. Thus, nowadays, genetic ancestry inference in an admixed population is an essential tool to control the effect of population stratification in association studies^35^, for the identification of disease-associated genes^36^, precision medical care^11^ and to reveal population history^37,38^.

Therefore, the correct assignment of each genetic ancestral component is essential for studies with admixed populations. Concerning this issue, some studies compared AIM panels in American admixed populations, observing differences in ancestry proportion inference between panels, which may be related to both the number of markers and parental reference populations of each panel^19,39,40^.

Of the Latin American countries, Brazil was the only Portuguese colony, which resulted in peculiarities in the admixture process. Brazil received more than 4.5 million African slaves whose origin in the African continent may differ in terms of place and time from those of non-Portuguese colonies in Latin America^37^. Approximately 3 million Native Americans (indigenous people) lived in the current Brazilian territory in the fifteenth century who, after contact with Europeans, declined in number by at least 90%^41^. Recently, in the twentieth century, Brazil continues to receive millions of immigrants, especially from East Asia^33^.

Today Brazil is the most populous country in Latin America and the seventh most populated country in the world, with more than 215 million inhabitants. Understanding how different marker sets are assigned to Brazilian genetic ancestry is of extreme relevance to both historical and public health issues. The first comparative studies to address this issue were only performed with AIMs or pharmacogenomic high-density SNP array panels and are based on the tri-hybrid admixture model (Native American, European and African)^19,39,40^. Herein, we present for the first time a comparison of ancestry estimates between different genetic marks sets—AIMs, high-density array of SNPs and Whole Genome Sequencing—in the Brazilian population using the tri-hybrid (African, European and Native American) and tetra-hybrid admixture models (African, European, Native American and East Asian).

This study analyzed 1,171 admixed samples from Brazil^23^, which we compared to (i) 5 AIMs panels: 34AISNP^42^ + PIMA^19^; 55AISNP^18^; 128AISNP^43^; 170AISNP^44^, and 446AISNP^45^, selected by both the tri- and tetra-hybrid admixed models. In addition, we evaluated the combination of the 5 aforementioned panels, referred to herein as 665AISNP; (ii) a high-density SNPs array (HDSNP), specially developed for population genetics studies and therefore without biased markers such as the GWAS SNP array or pharmacogenomics, and (iii) whole genome sequencing (WGS) data.

## Results

### Ancestry inference in parental population groups

The proposal of panel sets is to correctly assign individuals to their original continental groups, especially AISNPs. However, factors such as the number of markers and the set of populations used to establish allelic frequencies of the continental group may influence ancestry estimation accuracy. To better assess these issues, we analyzed the influence of marker number by comparing the inference of ancestry in our panel sets for the 4 main parental continental groups (African, European, East Asian, and Native American) which contributed most to the Brazilian population. For the parental populations, we used samples of the Human Genomic Diversity Panel (HGDP)^46^ and 1000 Genomes Project phase III (1KGP)^24^ (Supplementary Table S1).

First, we evaluated the accuracy of the panel sets in correctly assigning pre-categorized individuals^24,46^ into each of the 4 continental groups. Only a small proportion of samples were not assigned correctly (Z-score > |3|; p-value 0.01; ranging from 1.66 to 0.7% and 0.86 to 0.4% in the HGDP and 1KGP, respectively) (Supplementary Tables S2–S4).

**Figure 1:**
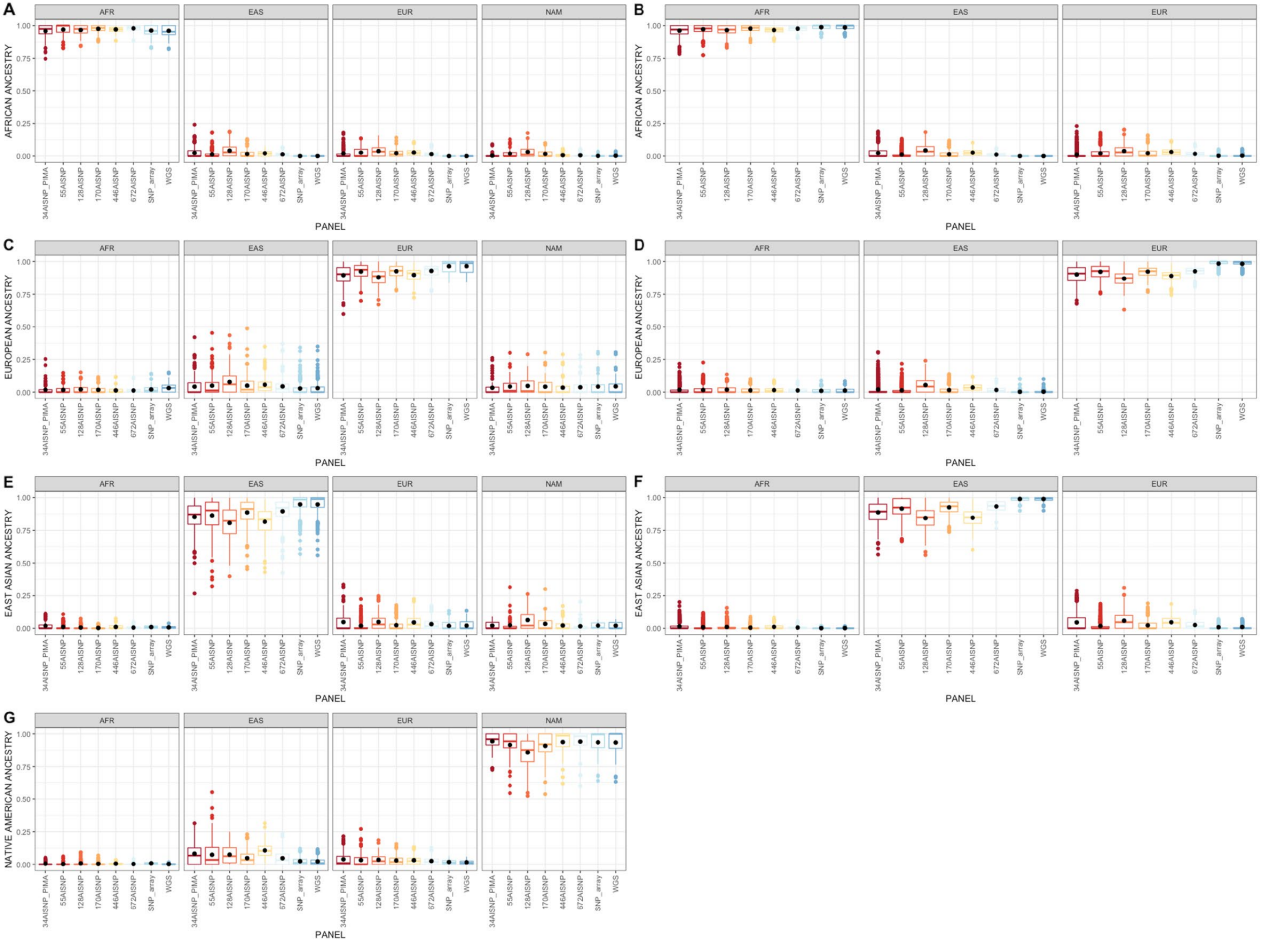
Boxplot with the distribution of ancestry inferences of individuals within each continental group. The boundary of the box closest to zero indicates the 25th percentile, the line within the box represents the median, and the boundary of the box farthest from zero indicates the 75th percentile. Black points within the box mark the average. Whiskers above and below the box indicate the 10th and 90th percentiles. Points above and below the whiskers indicate outliers outside the 10th and 90th percentiles. (**A**, **C**, **E** and **G**) refer to the HGDP samples. (**B**, **D** and **F**) relate to the 1KGP samples.

Secondly, the distribution of the inferred proportion of individual ancestry for each set of panels and continental group were verified (Fig. 1). For African continent samples, all panel sets had high accuracy in inferring African ancestry in both datasets (median > 96%, lowest observed dispersion ancestral components inferred). On the other hand, for other continental samples, some panel sets displayed greater dispersion in ancestry inferences, showing median and average values < 90%. The 128AISNP and 446AISNP panels had the lowest medians (82–88% and 83–89.5%, respectively) for samples from the continental European, East Asian and Native American groups. Meanwhile, the HDSNP and WGS panels had the lowest dispersions in all continental groups with median values > 98%.

Finally, a pairwise comparison of the inferred proportion of individual ancestry was performed between each panel set for each continental group (Supplementary Fig. S1A–G). The 8 panel sets evaluated showed high correlation coefficient values (r^2^ > 0.96), ranging from 0.98 to 1 for the African and European samples (Supplementary Fig. S1A,B,E,F) and from 0.96 to 1 for East Asians and Native Americans (Supplementary Fig. S1C,D,G).

### Ancestry inference in Brazilian populations

A common question when studying genetic ancestry of the Brazilian population is whether to use a tri- or tetra-hybrid admixture model, and what panel set. In order to explore this issue, we analyzed both the admixed models and the inference of ancestry from different panel sets—AIMs, HDSNP and WGS. For the parental reference populations, we selected Africans (AFR), Europeans (EUR), East Asians (EAS), and Native Americans (NAM) from the HGDP, only including samples with z-score values < |3| (Supplementary Tables S2–S4).

**Figure 2:**
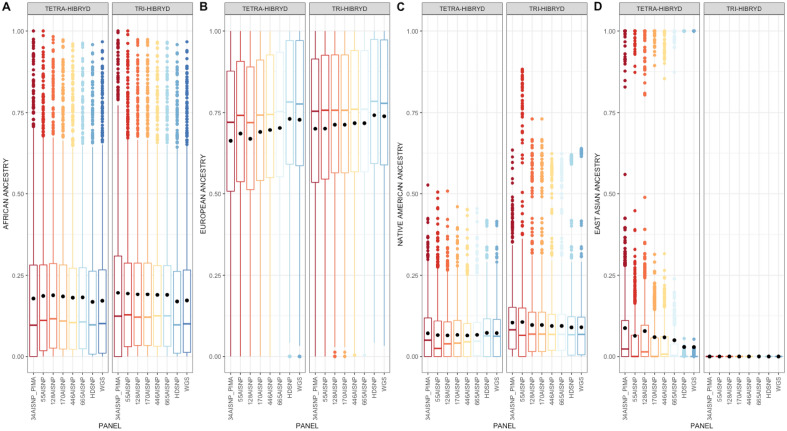
Boxplot with the distribution of ancestry inferences of individuals in the Brazilian population (SABE) for the tri- and tetra-hybrid models. The boundary of the box closest to zero indicates the 25th percentile, the line within the box represents the median, and the boundary of the box farthest from zero indicates the 75th percentile. Black points within the box mark the average. Whiskers above and below the box indicate the 10th and 90th percentiles. Points above and below the whiskers indicate outliers outside the 10th and 90th percentiles.

In general, we observed variations in ancestry inferences according to the admixed model chosen as well as the panel set (Fig. 2). By the tri-hybrid model, the average ancestry inferences in the Brazilian sample ranged from 70.02 to 74.16% for EUR ancestral component; 16.91 to 19.58% for AFR, and 8.96 to 10.59% for NAM (Supplementary Table S5). In the tetrahybrid model, the average ancestry inferences were: 66.33 to 73.02% (EUR ancestral component); 16.77 to 18–76% (AFR); 6.46 to 7.26% (NAM), and 2.90 to 8.72% (EAS) (Fig. 2; Supplementary Table S6).

**Figure 3:**
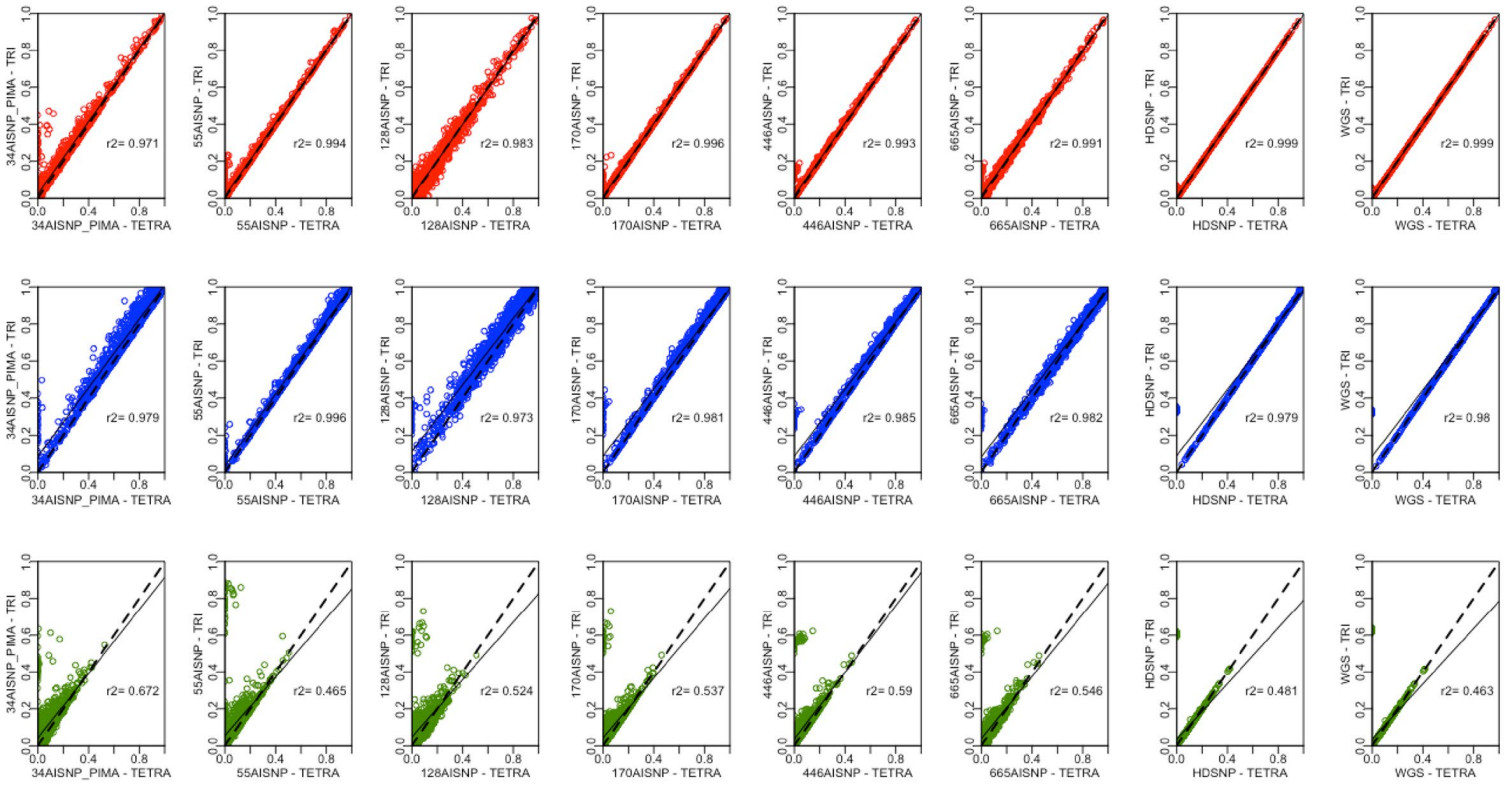
Pairwise comparison of ancestry inferences by tri and tetra-hybrid models for Brazilian samples (SABE) with the 8 panel sets evaluated: 34 AISNP + PIMA; 55 AISNP; 128 AISNP; 170 AISNP; 446 AISNP; 672 AISNP; HDSNP, and WGS. The x-axis corresponds to the ancestry inference by the tetra-hydride model for a given panel. The y-axis corresponds to the inference of genetic ancestry by the tri-hybrid model for a given panel. In the figure, r^2^ corresponds to the correlation coefficient, the black dashed line represents the trend, and the solid black line corresponds to the perfect agreement between two panels. The red, blue and green colors correspond to the inference for the African, European and Native American ancestral components, respectively.

To determine whether there are significant differences in ancestry inferences according to the tri- or tetra-hybrid models, we adopted two analytical approaches. First, as many studies are interested in the population average of each ancestral component, we performed the paired t-test to compare the average obtained with the same panel from the tri- and tetra-hybrid models (Supplementary Table S7). The averages for the AFR ancestry component inferred did not differ significantly between the models. On the other hand, all panel sets showed significantly different averages for the NAM component (t-test > 4; p-value < 0.0025), and the 128AISNP for the EUR component (t-test = 4.17; p-value = 3.13^–5^). We subsequently performed a pairwise comparison to verify the correlation between the ancestry inferences obtained by the same panel from the tri- and tetra-hybrid models. We observed that the inferred AFR and EUR ancestral component correlation coefficient ranged from 0.97 to 0.99, and the NAM component between 0.46 and 0.67 (Fig. 3).

To better understand how the EAS ancestral component is being detected by the admixture models, we evaluated the assignments of 33 samples in the Brazilian dataset, all of which were self-declared Asian descendants. In the tetra-hybrid model, all panels detected more than 85% EAS ancestral component in the samples analysed. We then analyzed this subset by comparing the inferences of AFR, EUR, and NAM components between the tri- and tetra-hybrid models. For the tetra-hybrid model, the inferences of these three ancestral components were close to 0, while between 20 and 66% in the tri-hydrid model. In Fig. 3, we see that in almost all comparisons, these samples are clustered and offset from the correspondence line. In order to assess how the EAS ancestral component was assigned in the other samples of the dataset, we excluded samples with > 85% Asian ancestry. The inferred EAS average for this subset was: 6.17% (s.d. = 8.5%) 34AISNP + PIMA; 3.69% (s.d. = 6.25%) 55AISNP; 5.31% (s.d. = 8.5%) 128AISNP; 3.24% (s.d. = 5.17%) 170AISNP; 3.25% (s.d. = 4.73) 446AISNP; 2.27% (s.d. = 3.55%) 665AISNP; 0.11% (s.d. = 0.36%) HDSNP, and 0.09% (s.d. = 0.33%) WGS. For HDSNP and WGS, none of the sample had EAS component inference above 5%, while the AIMs panels had samples with maximum observed values ranging from 23.9 to 55.9%.

Finally, we evaluated whether the ancestry inference from the different sets of panels differs from each other (Fig. 2). A pairwise comparison of the averages was performed with no significant differences (t-test) observed for the AFR and NAM component in both models (Supplementary Tables S8 and S9). With the tri-hybrid model, the inferences of the EUR ancestral component had significant differences in the comparisons of the 34AISNP + PIMA and 55AISNP panels to HDSNP and WGS with p-values of < 0.005 and 0.007, respectively (Supplementary Table S8). In the tetra-hybrid model, for inferences of the EUR component, with the exception of the 446AISNP and 665AISNP panels, the others showed significant differences to HDSNP and WGS panels (p-value < 0.01). Additionally, for the EAS component, only 665AISNP had no significant difference with HDSNP and WGS (Table S9). We also performed correlation analysis between panels for ancestry inference for the inference of the AFR ancestral component (r^2^_tri_ = 0.89 to 1; r^2^_tetra_ = 0.90 to 1); EUR (r^2^_tri_ = 0.91 to 1; r^2^_tetra_ = 0.91 to 1); EAS (r^2^_tetra_ = 0.80 to 1), and NAM component (r^2^_tri_ = 0.76 to 1; r^2^_tetra_ = 0.54 to 0.99) (Supplementary Figs. S3 and S4).

### Ancestry inference in admixed American populations

To find out if this pattern observed in the Brazilian sample is similar in other admixed populations in America continent, we performed the same analyzes in the admixed populations of 1KGP: Afro-Caribbean (ACB); Afro-American (ASW); Colombian (CLM); Mexican (MXL); Peruvian (PEL), and Puerto Rican (PUR). The admixed populations evaluated herein have different proportions of parental ancestry, ranging from those with proportions of mostly African ancestry (ACB and AWS), mostly European ancestry (CLM, PUR), and mostly Native American ancestry (MXL and PEL) (Supplementary Tables S5 and S6).

When comparing ancestry averages inferred by the same panel for each ancestry component with tri- or tetra-hybrid admixture models, nonsignificant differences were observed (except for the 128AISNP panel in the NAM component in the PUR sample; t-test = 3.7, p-value = 0.029) (Supplementary Table S7). On the other hand, the pattern of correlation coefficients is heterogeneous between the ancestry components and the 1KGP admixed populations (Supplementary Fig. S4F). In the comparisons of ancestry inference averages by the different panels in the same admixture model, we also found heterogeneous results. In the tri-hybrid model, ACB showed differences between the averages inferred for the EUR and NAM componentes, and MXL for the AFR component (Supplementary Table S8). The pairwise comparison of individual ancestry inferences between the panels shows variation in the correlation coefficients, both between ancestry components in the admixed population, and between the admixed populations. In ACB, the correlation coefficient for AFR ancestry component ranged from r^2^_TRI_ = 0.66 to 1 (Supplementary Fig. S11A) and in ASW from r^2^_TRI_ = 0.88 to 1 (Supplementary Fig. S12A). In CLM, the correlation coefficient for the EUR ancestry component ranged from r^2^_TRI_ = 0.78 to 1 (Supplementary Fig. S13B), and from from r^2^_TRI_ = 0.74 to 1 in PUR (Supplementary Fig. S16B). Regarding MXL and PER, the correlation coefficient for NAM ancestry ranged from r^2^_TRI_ = 0.87 to 1 (Supplementary Figs. S13C and S15C). A general trend in these comparisons is higher correlation coefficients between panels that share a greater number of markers (e.g. 128AISNP × 170AISNP; 446AISNP × 665AISNP and HDSNP × WGS), in addition to those with the highest number of markers (e.g. 446AINSP, 665AISNP, HDSNP and WGS) (Supplementary Figs. S11–S16).

## Discussion

In the present study, we evaluated 8 panel sets: six AISNPs, one HDSNP and one WGS. Using tri- and tetra-hybrid admixture models, we compared ancestry inferences in Brazilian admixed populations and a set of admixed American populations.

To verify the accuracy of the panels, samples from HGDP and 1KGP datasets were used, whose geographic origin is known and without evidence of recent admixture. Despite the low marker overlap observed in the AISNPs panels (see Supplementary Material Notes), all panels showed a high accuracy rate (error rate 0.4–1.66%; Supplementary Table S2) and high degree of correlation in the pairwise comparisons of the panels (r^2^ > 0.96; Supplementary Figs. S1A–G). However, it is also possible to observe heterogeneity in the distribution of genetic ancestry inferred within each parental group by the different panel sets (Fig. 1). The smallest dispersion was observed in AFR (median values > 90%), while EUR and EAS presented the largest one (median values < 90% in panels such as 128AISNP and 446AISNP).

These results reveal, albeit with varying degrees of accuracy between them, that the available AISNP panels meet the proposed role of correctly attributing ancestry according to the continental group to which the individual belongs. Several studies already compared the accuracy of panels and obtained similar results^18,21,47^. As such, many authors currently argue that there is no necessity for new AIMs panels to assign the 6 biogeographic regions: Sub-Saharan Africa, Europe, Southwest Asia, South Asia, East Asia and the Americas. Instead, efforts should be directed towards building panels for global use, with greater representation of population groups^18^.

Most AIMs panels use HGDP and 1KGP data as a reference population for marker selection, including some of those evaluated in the present study^19,42^. These two public databases were essential for understanding the distribution of genetic diversity and affinity among human population groups^24,46,48^. However, they only capture a portion of human population diversity. Therefore, many AIMs panels endeavoured to include more populations from different population groups during their development process, for example: 55 AISNP^18^; 128 AISNP^43^; 446 AISNP^45^.

Soundararajan et al.^49^ argued that if there is a low representation of data from reference populations, a greater number of markers becomes necessary for the robustness of allele frequencies for the definition of population groups of interest. Our results converge at this point as we observed greater correspondence in individual ancestry inferences between panels with a greater number of markers, in particular to those of the HDSNP and WGS data.

In the present study, we focused on Brazilian admixed populations. This population emerged in the last half-century, especially from Native American, European, and African sources. More recently, it has also received contributions from other regions, including East Asia and the Middle East.

Admixed populations require a closer look in terms of ancestry inferences as their genomic particularities give rise to several challenges. Each admixed population has a peculiar evolutionary history, differing in parental sources, proportion and time of admixture. Furthermore, the admixing process produces variation at different levels: in ancestry between admixed populations, between individuals in the same admixed population, and throughout the genome of the same admixed individual^34^. For this reason, a method, model or panel that captures the profile in one admixed population or admixed individual well will hardly have the same performance for another.

We know that the EAS contribution is less than 1% for most of the Latin American admixed populations. Therefore, the choice of tri- or tetra-hybrid model will depend on the admixture profile of the population. Our motivation to analyze the tetra-hybrid model lies in the fact that in recent decades there has been a growing migratory flow of East Asian populations to large urban centers in the USA and Brazil. East Asian immigration to Brazil began in 1908 with the Japanese and today, according to the Ministry of Foreign Affairs of Japan, more than 2 million Japanese descendants live in Brazil. São Paulo, the city where the Brazilian samples of the present study were collected, is home to one of the largest Japanese communities outside Japan. The Brazilian cohort has 33 samples with 100% East Asian ancestral component that are direct descendants of the first Japanese immigrants^23^. Data from the last Brazilian census revealed that, in 10 years, there was a 173.7% increase in the number of individuals who declared themselves to be of Asian descent (Japanese, Chinese and Korean)^33^.

Based on this scenario, using WGS data from 1171 Brazilian individuals, we evaluated how different admixed models and sets of panels behave to infer ancestry in the Brazilian population. First, we checked for differences in the inferences of each ancestral component according to the tri- or tetra-hybrid admixture model. The population average inferred by either admixed model only differed for the NAM ancestral component (Supplementary Table S7). Similarly, the NAM ancestral component is the one with the lowest degree of correlation between the two admixture models (Supplementary Fig. S3). These results suggest that the chosen admixture model can influence the inference of the average NAM ancestral component in this Brazilian sample. In Figs. 2 and 3, it is also possible to observe a trend of greater proportions in the inference of the NAM ancestral component in the tri-hybrid model than in the tetra-hybrid model, both in terms of the population average and the individual. In order to better understand this trend, it is necessary to evaluate the assignment of the EAS ancestral component in these samples.

Once we had self-declared individuals of Asian descent in this Brazilian cohort, we verified how the tri- and tetra-hybrid models assigned ancestry. For these individuals, the tri-hybrid model, the HDSNP and WGS panels assigned: ~ 63% to NAM, ~ 32% to EUR, and ~ 5% to the AFR ancestral components. There are more ranges of inferred percentage for the AIMs panels (Fig. 3). The panels with the highest number of markers (446AISNP and 665AISNP) were closer to the inferences of high-density panels of SNPs, while those with the lowest number of markers (34AISNP + PIMA, 55AISNP, 128AISNP and 170AISNP) had large ranges, in some cases including the assignment of proportions for the African ancestral component > 40%. This result shows a redistribution of the EAS component, mostly to the NAM component, followed by the EUR component, and to an even smaller proportion, the AFR component. As the NAM ancestral component is a minority in the Brazilian cohort (< 8%), this may constitute to the increase of the NAM ancestral component in the average population discussed in the previous paragraph.

Given the recent migratory flow from East Asia to Brazil and the fact that the samples from the Brazilian cohort were collected in 2010 and had individuals > 60 years old (71.86 ± 7.94) at the time of collection (details in^23^), it was unexpected to visualize individuals with this ancestral component as a minority in their genome. Thus, we evaluated the remaining 1138 samples as probably not possessing the EAS ancestral component. Our results showed that for the tetra-hybrid model, especially for the AIMs panels, there was more noise in the EAS component inference (Fig. 2), while for the HDSNP and WGS panels, the inferences had less noise (no individual with > 5%). These results suggest that the two high-density marker panels are able to better assign ancestral components in the tetrahybrid model. In turn, in the trihybrid model, samples with some proportion of the EAS ancestral component in the tetrahybrid model, showed an increase in the NAM and EUR ancestral components. This observation can be seen as another factor contributing to the differences in the inferred Native American ancestral component between the admixed models.

We also compared the tri- and tetra-hybrid models in other admixed populations in America (1KGP) for which there are no historical records of large migratory flows from EAS (except for Peru). Therefore, it is unusual to analyze the tetra-hybrid model in this dataset and we only performed it in order to better explore the patterns. Unlike the Brazilian cohort, we did not observe significant differences in the population averages of the components between the admixed models. However, Figs. S5–S10 clearly show noise with the inference of the EAS ancestral component in populations for which it is not part of the parental source.

Therefore, choosing an admixed model is not a simple decision, as each model has advantages and disadvantages in each population. The decision of which model to apply will depend on the question the investigator wants to ask and whether there is interest in the population average or ancestry of each individual in the sample. If it is to decipher specific admixture components, for example to learn about an individual’s family migratory patterns, then all possible parental populations involved should be included. If they are simply trying to determine the major ancestral component, for example exclusion purposes, then a smaller model with the key continental groups may suffice.

The second objective of our study was to compare ancestry inferences with different sets of panels (AISNP, HDSNP and WGS). In the Brazilian sample, we observed significant differences for the EUR ancestral component averages in the tri-hybrid model (34AISNP + PIMA and 55AISNP × HDSNP and WGS) (Supplementary Table S8) and for the EUR (34AISNP + PIMA, 55AISNP, 128AISNP and 170 AISNP × HDSNP and WGS) and EAS (all panels, except 665AISNP × HDSNP GWS) in the tetra-hybrid model (Supplementary Table S9). These results are possibly related to what was observed for the parental populations, where there is greater dispersion in the distribution of EUR, EAS and NAM ancestry (Fig. 1), indicating a variation in the accuracy of correctly assigning this ancestral component. On the other hand, we did not observe differences in the population averages inferred by the sets of panels for the AFR and NAM ancestral components. Although, in the pairwise analyses, the smallest correlations between panels occurred in inferences from the NAM ancestral component (Supplementary Figs. S2C and S3C). This result suggests that although the population average inference of the NAM ancestral component is similar between the panels, there are differences in the inferences on the individual level.

The analysis involving admixed populations of the 1KGP showed heterogeneous results. In paired comparisons of individual ancestry inference between panels (Supplementary Figs. S11–S16), we observed variation correlation coefficients both between ancestry components within the same admixed population and between admixed populations. The inconsistencies observed in the ancestry inferences between the panels were even more evident for the minority ancestry components of the individuals in our results (e.g. Supplementary Figs. S14A, S15A and S16C). This probably occurred because the genome of an admixed individual is a mosaic composed of segments from different parental sources. Over generations, due to the process of meiotic recombination, the components of distinct ancestry are shuffled between homologous chromosomes^36,50^. Thus, the greater the number of generations since admixture onset, the smaller the size of the genomic segments of the ancestry will be. In addition, the greater the proportion of an ancestral component, the greater the size of its segments in the genome, while conversely, the smaller the proportion of the ancestral component, the smaller the segments in the genome^50^. In this scenario, due to lower density and genomic coverage, AISNP tends to be less accurate data than higher SNP density and higher genomic coverage.

The NAM component had the lowest correspondence in ancestry inferences between the panels (Supplementary Figs. S3C and S16D). It is widely recognized that the Native American populations, due to their recent bottleneck history, are the most differentiated in the world^48^ having the lowest number of representatives in the reference panels. Panels were developed with the aim of enriching the NAM component^18,43,45^, however, they do not always capture this component well in all admixed populations. Thus, the underrepesentation of Native American sources, in addition to the minority NAM ancestral component in PUR and Brazilian sample, may be contributing to the observed differences in ancestral inference between the panel sets for this ancestral component.

Through the present study, we verified that there are differences in the inferences of the ancestral components according to the panel chosen. There is greater correspondence of inference between panels that share a greater number of markers (128AISNP and 170AISNP; 446AISNP and 665AISNP; HDSNP and WGS), and among those with the highest number of markers (446AISNP, 665AISNP, HDSNP and WGS). Again, it is important to point out that the choice of panel will depend on the purpose and needs of the study. For example, in forensic genetics, sometimes samples with quantity and quality are not available, which limits the genotyping methodology^51^. Meanwhile, in clinical or genetic association studies, accurate genomic ancestry is essential. Furthermore, it is often necessary to go a step beyond the genomic average and make inferences about ancestry in specific genomic segments^52,53^.

The admixed populations of America are being increasingly studied in terms of population history, clinical and forensic studies. Therefore, nowadays, it is essential to discuss and understand how methodological advances, both in genotyping and in analysis, help to improve the inference of genetic ancestry in admixed populations. In the present study, we analysed data from WGS, HDSNP and AIMs in a Brazilian samples, through different admixture models and compared with other admixed populations of the American continent. We showed that heterogeneity within and between admixed populations still poses methodological challenges. Therefore, it is fundamental when defining the research question, to be aware of the advantages and limitations of each admixture model and set of panels for the populations of interest.

## Materials and methods

### Datasets

Samples from 3 datasets were analyzed: (i) Human Genomic Diversity Panel (HGDP)^46^; (ii) 1000 Genomes Project phase III (1KGP)^24^, and (iii) Brazilian Cohort of Health, Well-being and Aging (*Saúde e Bem Estar*—SABE)^23^.

Based on the American admixture history, analyses were performed with parental samples from African, European, East Asian and Native American populations of HGDP (543 individuals) and 1KGP (1511 individuals) as described in Table S1. We also analyzed the 504 samples from the 6 admixed populations from the 1KGP, and the 1,171 Brazilian samples from the SABE cohort (Supplementary Table S1).

All samples were genotyped by WGS and are publicly available (https://www.internationalgenome.org/data—HGDP and 1KGP; https://ega-archive.org/studies/EGAS00001005052—SABE). All individuals enrolled in the SABE cohort signed written consent forms to participate in this study approved by local and national institutional review boards: COEP/FSP/USP OF.COEP/23/10, CONEP 2044/2014, and CEP HIAE 1263-10.

### Ancestry informative markers SNPs panel (AISNP panel)

Due to availability in the 3 datasets, we evaluated only SNPs as AIM. Based on this criterion, we selected 5 AIMs panels frequently used in studies with Latin American populations: 34AISNP^42^ + PIMA^19^; 55AISNP^18^; 128 AISNP^43^; 170 AISNP^44^; 446 AISNP^45^. In addition, we also evaluated the combination of the 6 panels, which we named 672 AISNP. The SNPs of the AIMs panels used in the present study are described in Supplementary Table S2.

### High-density SNP chip array (HDSNPs panel)

Axiom™ Genome-Wide Human Origins (~ 600 K SNPs—ThermoFisher Scientific) was selected as a representative of high-density SNP arrays. This genotyping panel was optimized for population genetic studies and developed from genomic markers identified in 11 human populations: France, China, Papua New Guinea, San, Yoruba, Mbuti pygmies, Karitiana, Italy-Sardinia, Melanesia, Cambodia, and Mongolia, avoiding confounding biases introduced using GWAS SNP arrays.

### Merge datasets

Based on the WGS data from the 3 datasets, the following sets of SNPs were selected: (i) *AISNP panels*: 672 SNPs comprise the 6 AISNP panels selected for the present study. Of these, 5 SNPs (rs12402499; rs17287498; rs1321333; rs10954737; rs10071261) are not detected in all datasets (Supplementary Table S9), of which, 3 SNPs are informative of Native American ancestry and 2 of African ancestry; (ii) *HDSNPs panel*: ~ 600,000 SNPs that comprise the Axiom Human Origins array. The overlap between the 3 datasets was 555,168 SNPs, and (iii) *WGS data*: the original datasets with more than 60 million variants described. For the present study, we excluded SNPs: (a) MAF < 1%; (b) missing data per SNP > 1%; (c) Hardy–Weinberg p-value < 1 × 10^–8^, and (d) filter for LD coefficient (r^2^ = 0.1—see “Supplementary Material” section for more details). The final dataset contains 2,018,023 SNPs. For each set of markers, the 3 datasets (HGDP, 1KGP, SABE) were merged using vcftools v.0.1.15^54^ and plink v.1.9^55^. To validate these merge data, a PCA analysis was performed (Supplementary Fig. S17). Throughout the text we refer to AISNP, HDSNP and WGS as "panel sets".

### Data analysis

Allelic frequency inferences, Hardy–Weinberg and Fisher's exact test was performed using the PLINK v.1.9 software^55^. Correction for multiple testing was made according to Bonferroni correction.

### Genetic ancestry inference

ADMIXTURE v.1.3^56^ was used to perform global ancestry inference. Analyses were performed in an unsupervised manner when considering only parental populations, and in a supervised manner when considering admixed populations. Parameters used: 4 clusters (K = 4) and 2000 bootstrap replicates. Each analysis was repeated 10 runs and the results combined using the CLUMP software (v.1.222)^57^.

Information redundancy may occur in high-density SNP data (HDSNP and WGS). Therefore, to minimize and evaluate background linkage disequilibrium in the analyses, we also tested some disequilibrium linkage coefficients (r^2^ = 0.01, 0.05, 0.1, 0.3 and 0.5), assuming a distance not closer than 200 Kb between adjacent markers^55,56^.

Comparisons of ancestry inference were performed by correlation analysis and z-score test by R scripts using package stats v.4.1.1^58^.

## Supplementary Information

Supplementary Information 1.

Supplementary Tables.

## Data Availability

The datasets reported in this article are publicly available (https://www.internationalgenome.org/data—HGDP and 1KGP; European Genome-phenome Archive (EGA), under EGA Study accession number EGAS00001005052—SABE).
