# Supplementary material for: Challenges in selecting admixture models and marker sets to infer genetic ancestry in a Brazilian admixed population
Source: Sci Rep. 2022 Dec 8;12:21240. doi: 10.1038/s41598-022-25521-7 (PMC9731996; doi:10.1038/s41598-022-25521-7)
Supplement: Supplementary file 1 — Supplementary Information 1. [file 41598_2022_25521_MOESM1_ESM.docx]

**Supplementary Material**

**Challenges in selecting admixture models and marker sets to infer genetic ancestry in a Brazilian admixed population**

Luciana Maia Escher^1^, Michel S. Naslavsky^2,3^, Marília O. Scliar^3^, Yeda A. O. Duarte^4,5^, Mayana Zatz^2,3^, Kelly Nunes^2*+^, Silviene F. Oliveira^1*+^

^1^ Human Genetics Laboratory, Institute of Biological Sciences, University of Brasilia, DF, Brazil.

^2^ Department of Genetics and Evolutionary Biology, Biosciences Institute, University of São Paulo, São Paulo, SP, Brazil.

^3^ Human Genome and Stem Cell Research Center, University of São Paulo, São Paulo, SP, Brazil.

^4^ Medical-Surgical Nursing Department, School of Nursing, University of São Paulo, São Paulo, SP, Brazil.

^5^ Epidemiology Department, Public Health School, University of São Paulo, São Paulo, SP, Brazil.

* [knunesbio@gmail.com](mailto:knunesbio@gmail.com); [silviene.oliveira@gmail.com](mailto:silviene.oliveira@gmail.com)

^+^ Authors contributed equally to this work.

**NOTES**

**SNP overlap between AISNP panels**

In the present study, we evaluated five previously described AIMS panels: 34AISNP44+ PIMA17; 55AISNP22; 128AISNP47; 170AISNP55; 446AISNP55 (Supplementary Table N1). Of the total set of 665 AISNPs, only 25 SNPs (3.8%) overlapped with one or more AISNP panels (Figure N1). The panel with the highest number of overlaps is 55AISNP (six SNPs with 34AISNP + PIMA; 13 with 128AISNP and three with 446AISNP).

**
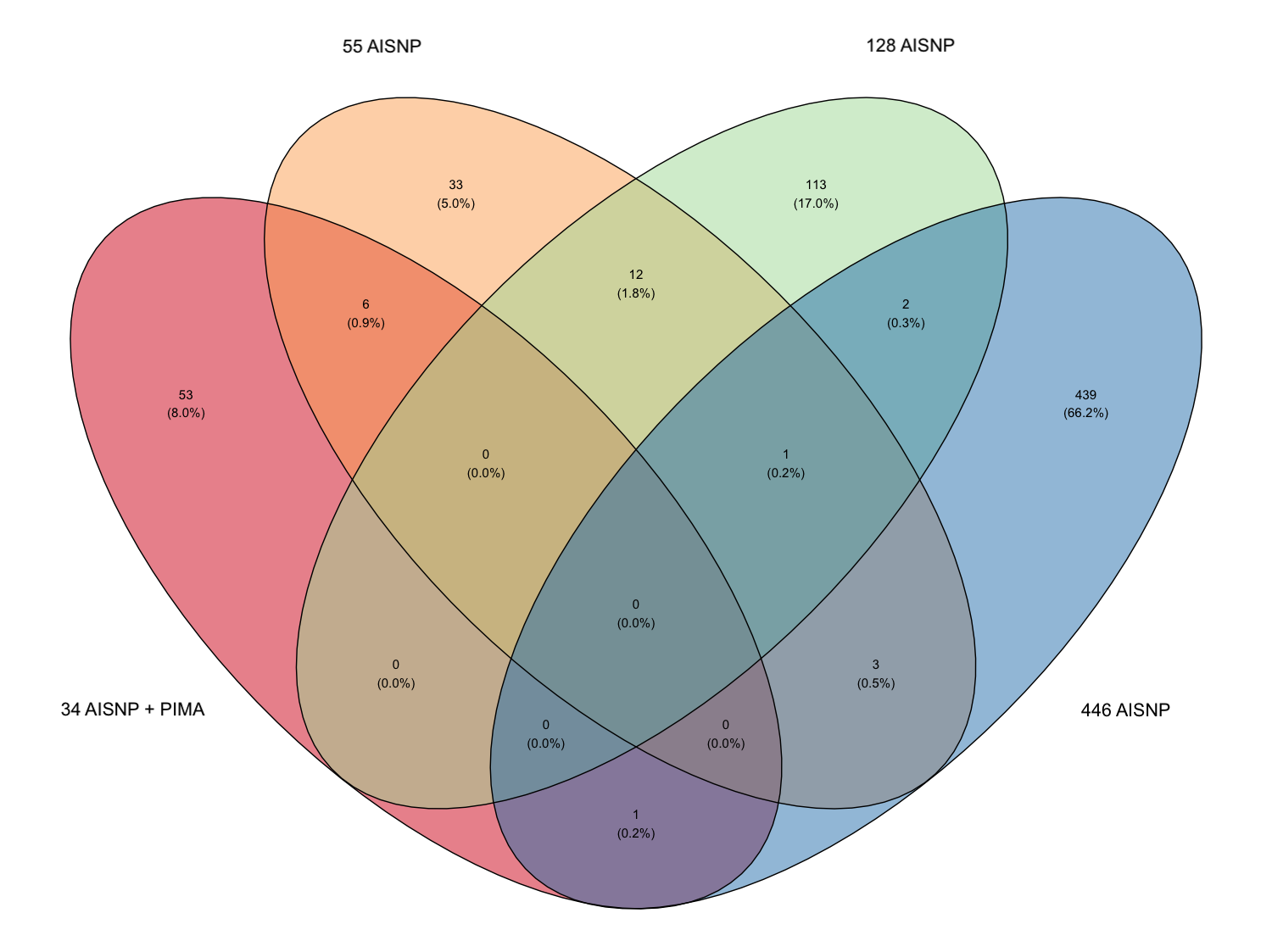
**

**Figure N1.** **Four-dimensional Venn diagram.** Venn diagram showing overlapping in the set of SNPs between the analyzed AISNP panels. The SNPS of the 34AISNP panels were merged with PIMA, resulting in 34AISNP+PIMA, and 55AISNP with 128AISNP resulting in 170AISNP.

**Prune Linkage disequilibrium (LD) in WGS data**

Comparison between AISNP panels and WGS data requires the same methodology for ancestry inference. Here we chose to use the ADMIXTURE software, for which the model assumes independent segregation between SNPs (Alexander et al., 2009) and therefore dense marker sets should be pruned to mitigate background linkage disequilibrium. To determine the optimal linkage disequilibrium coefficient (R^2^) for our WGS dataset, we evaluated several values (R^2^= 0.01, 0.05, 0.1, 0.3 and 0.5). The LD-pruned subsets of SNPs were used to infer the genetic ancestry of parental individuals in the HGDP and 1KGP datasets.

The comparative analysis of ancestry inference in the parental groups (African, Europe, East Asian and Native American) using the LD-pruned SNPs subsets shows a high degree of correlation between them (r^2^=0.997 to 1) and all comparisons between the subsets of SNPs with LD coefficients above 0.05 had r^2^=1 (Supplementary Figure N2). We also evaluated the inferred proportion of ancestry from each LD-pruned SNPs subset in each parental group (Supplementary Figure N3) and quantified how much the observed median and mean values differed from the maximum (1.00) expected value in each parent group. Since the LD coefficient of 0.01 had the smallest difference (Supplementary Table N2), the ancestry inference analyses were performed with a linkage coefficient equal to 0.1 for the HDSNPs panel and WGS data.

**
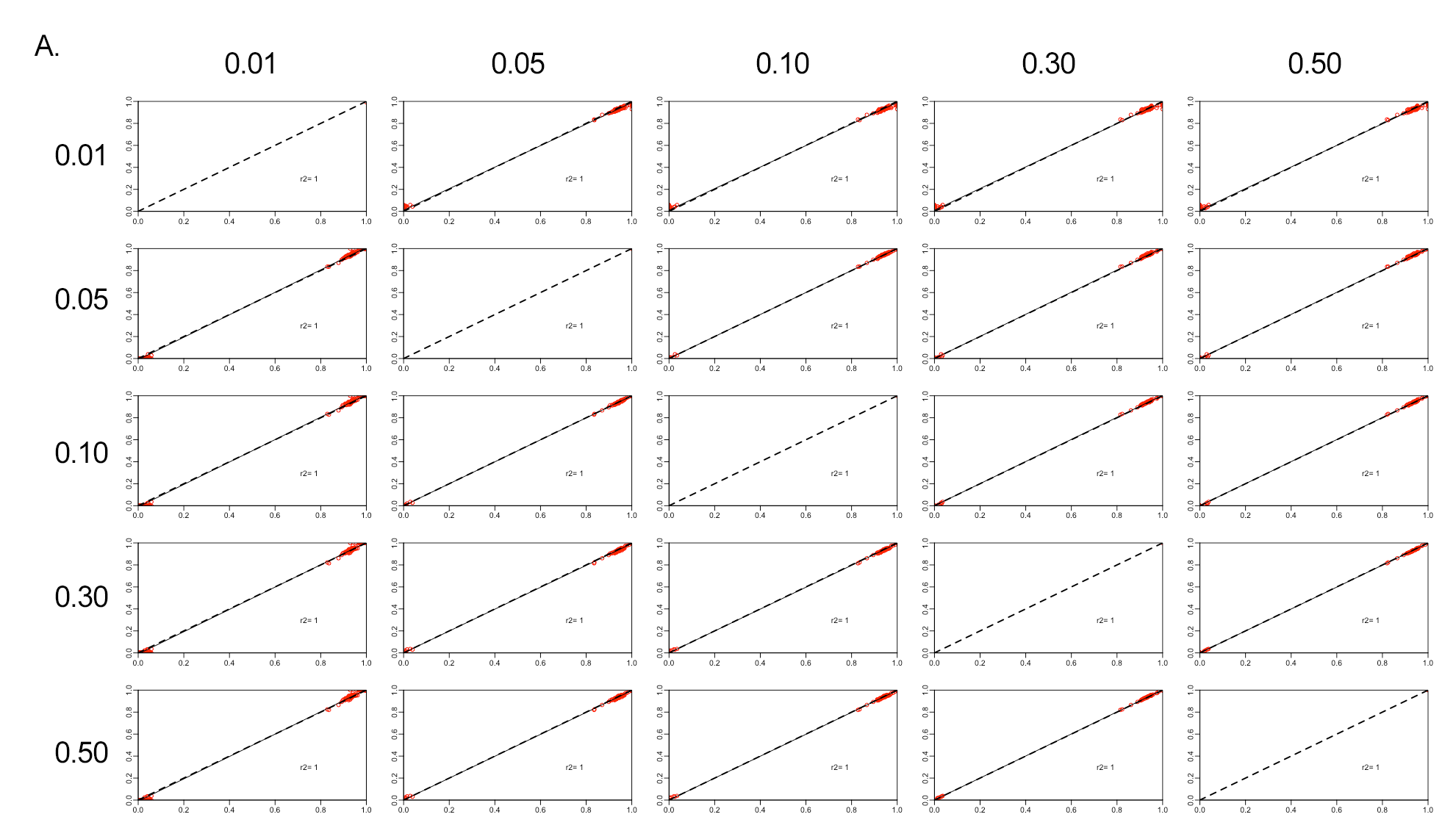

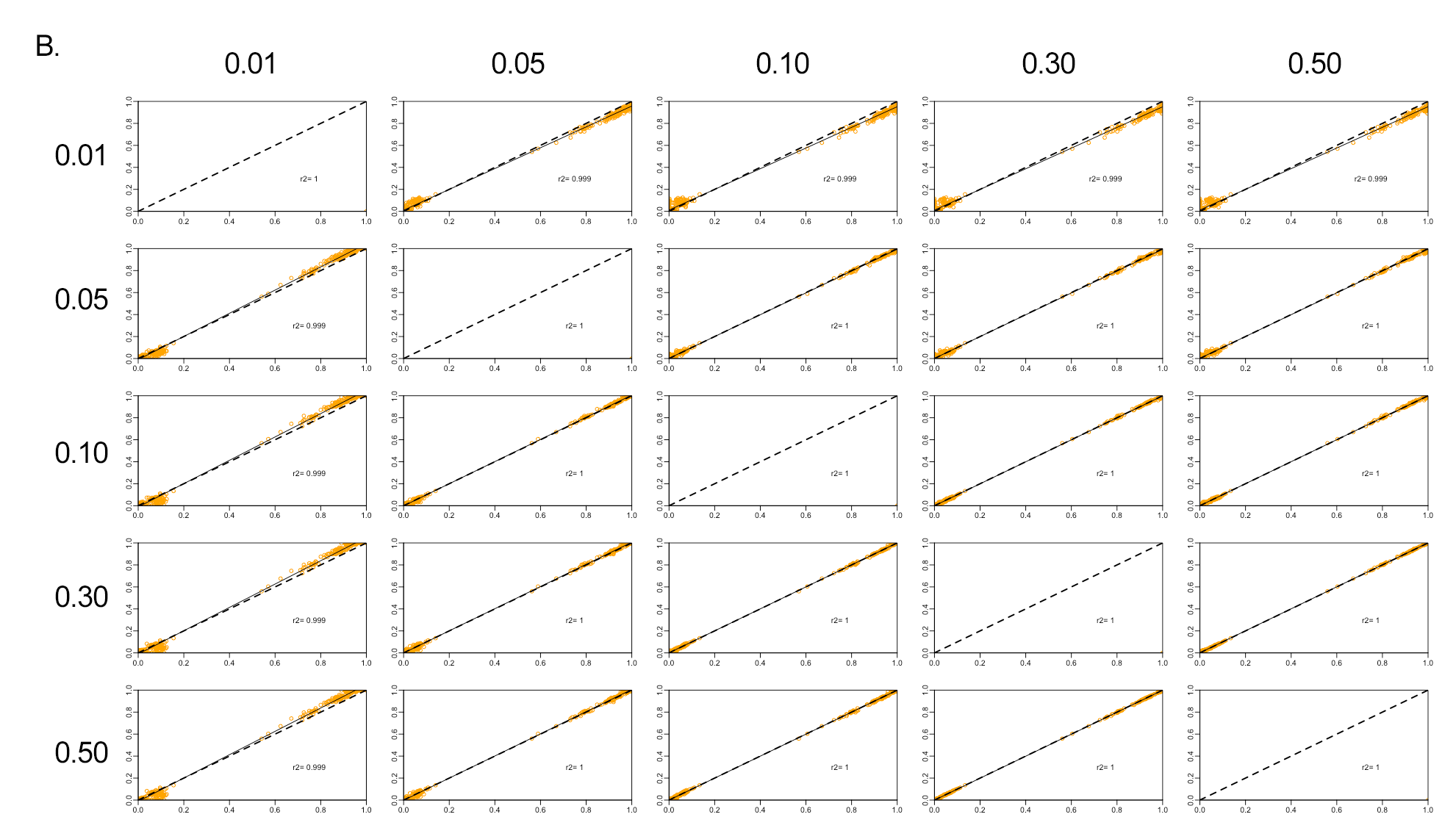

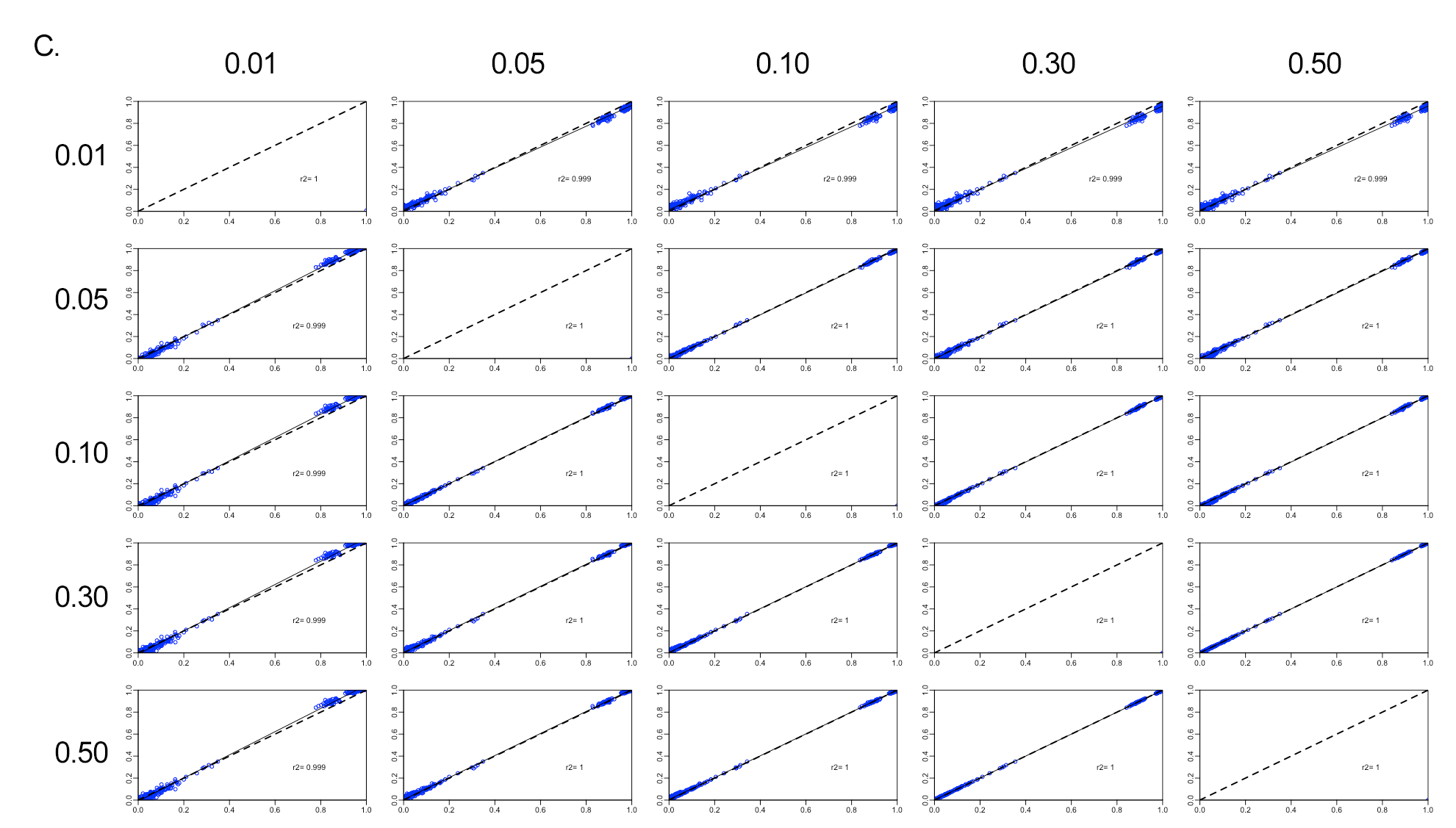

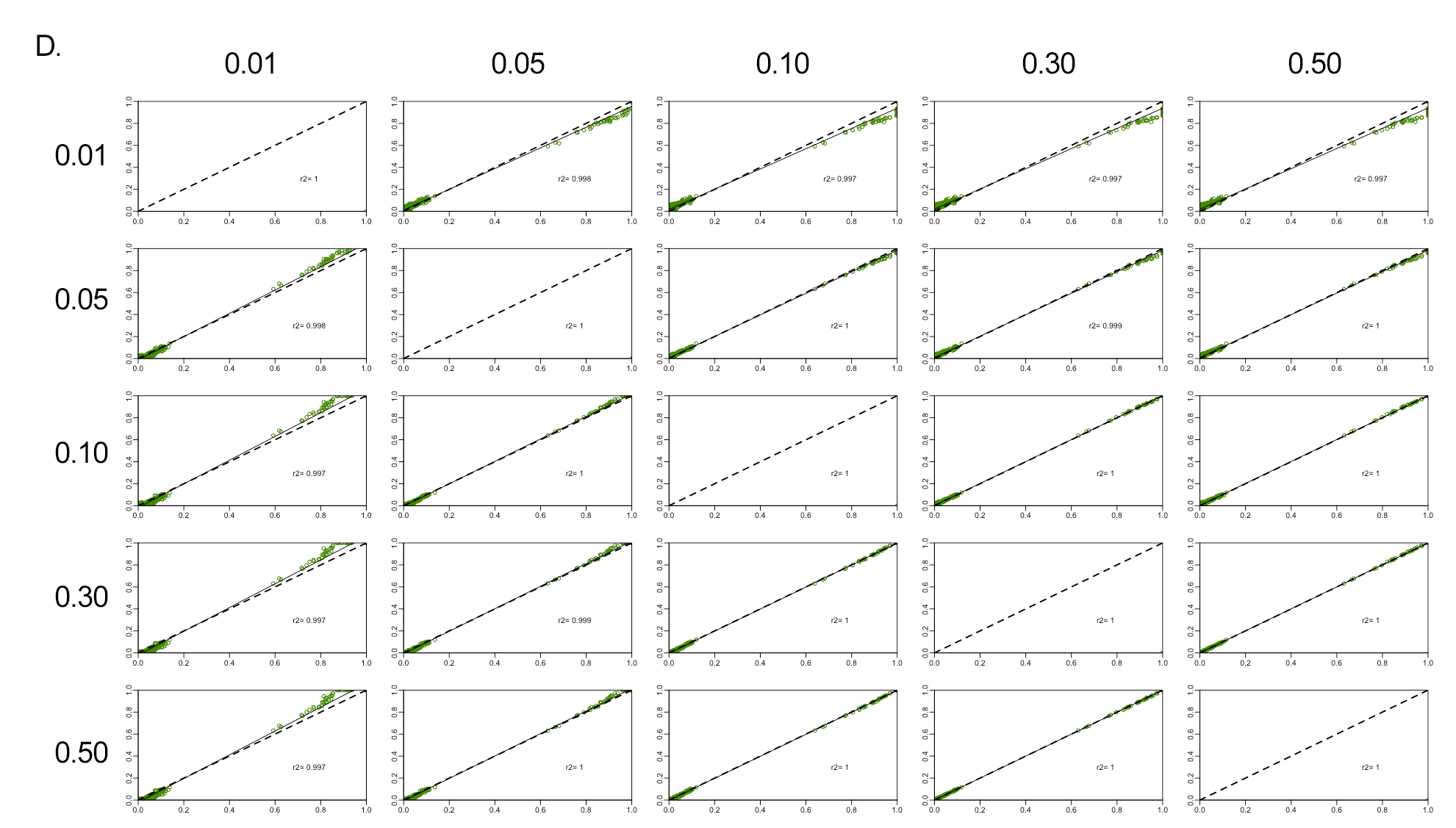
**

**Figure N2.** **Pairwise comparison of ancestry inferences for the parental HGDP-WGS data with different linkage disequilibrium coefficients (0.01, 0.05, 0.1, 0.3 and 0.5).** In the figure, r2 corresponds to the correlation coefficient, the black dashed line represents the trend, and the solid black line the perfect agreement between two panels. (A) corresponds to African (red), (B) East Asian (orange); (C) European (blue) and (D) Native American (green), respectively.


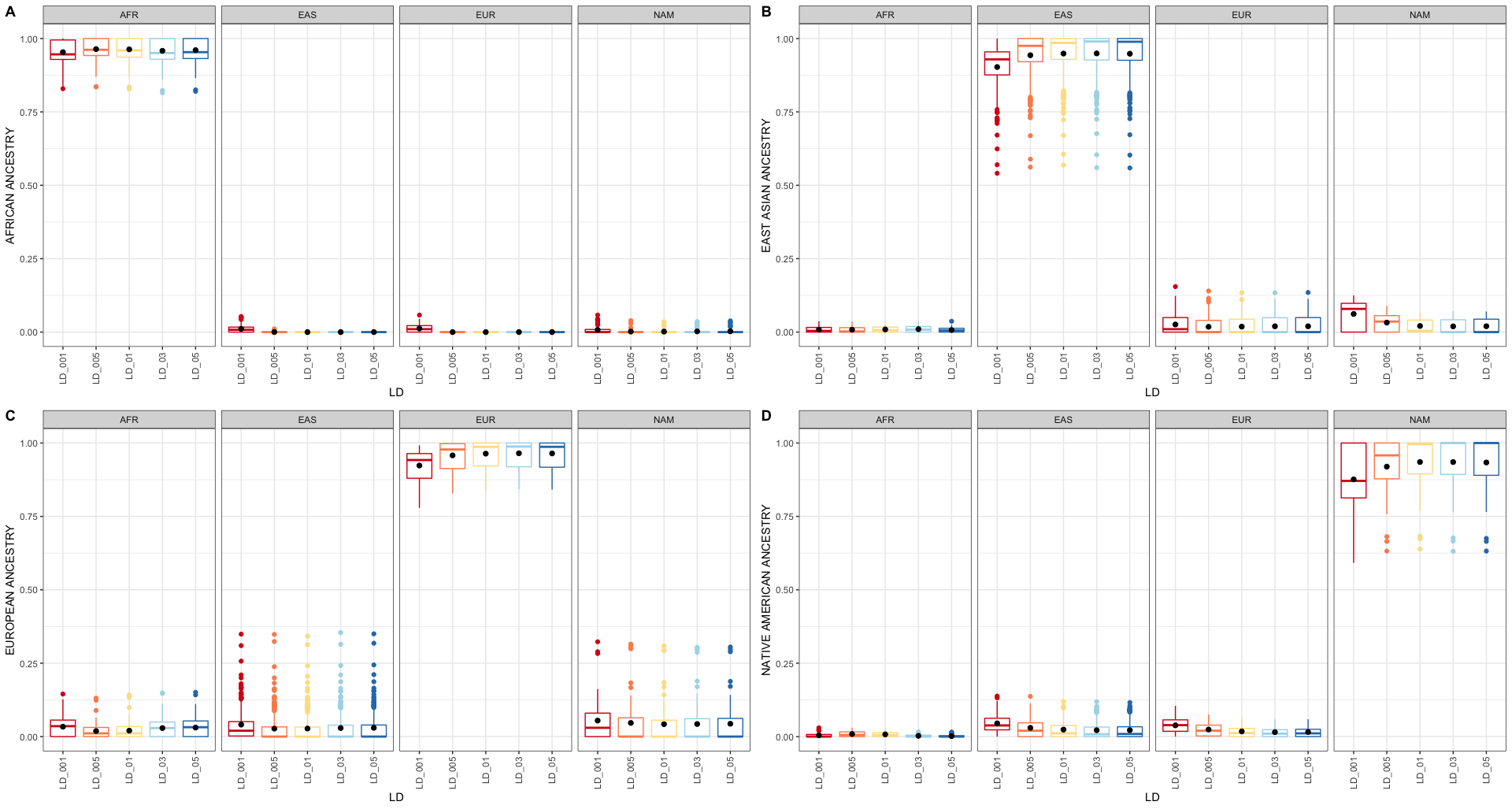


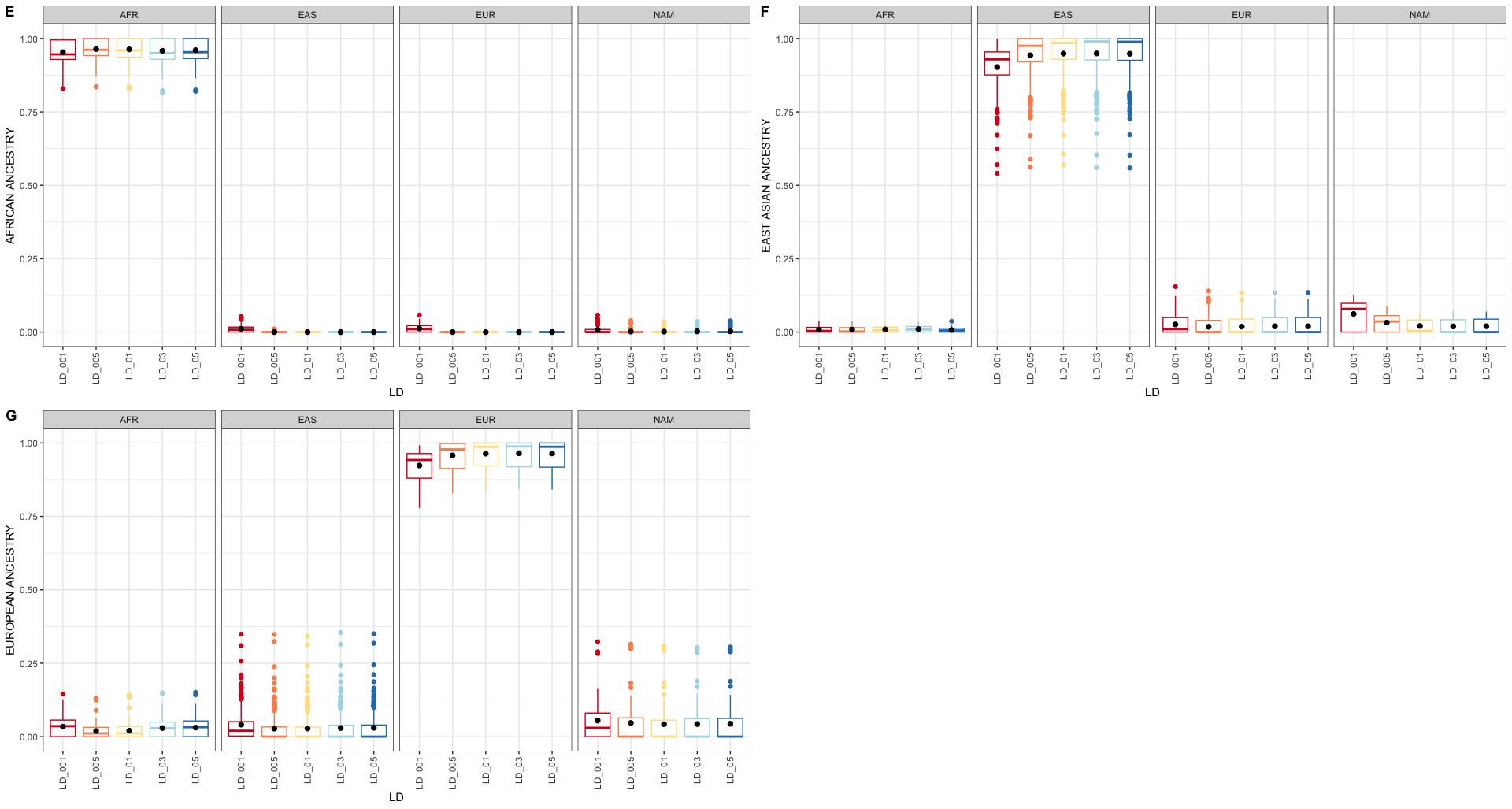


**Figure N3. Boxplot with the distribution of ancestry inferences of individuals within each continental group using different thresholds for the link disequilibrium coefficient.** The boundary of the box closest to zero indicates the 25^th^ percentile, the line within the box represents the median, and the boundary of the box farthest from zero indicates the 75^th^ percentile. Black points within the box mark the mean. Whiskers above and below the box indicate the 10^th^ and 90^th^ percentiles. Points above and below the whiskers indicate outliers outside the 10^th^ and 90^th^ percentiles. Figures A-D refer to the HGDP samples, and E-G the 1KGP samples.

**SUPPLEMENTARY FIGURES**


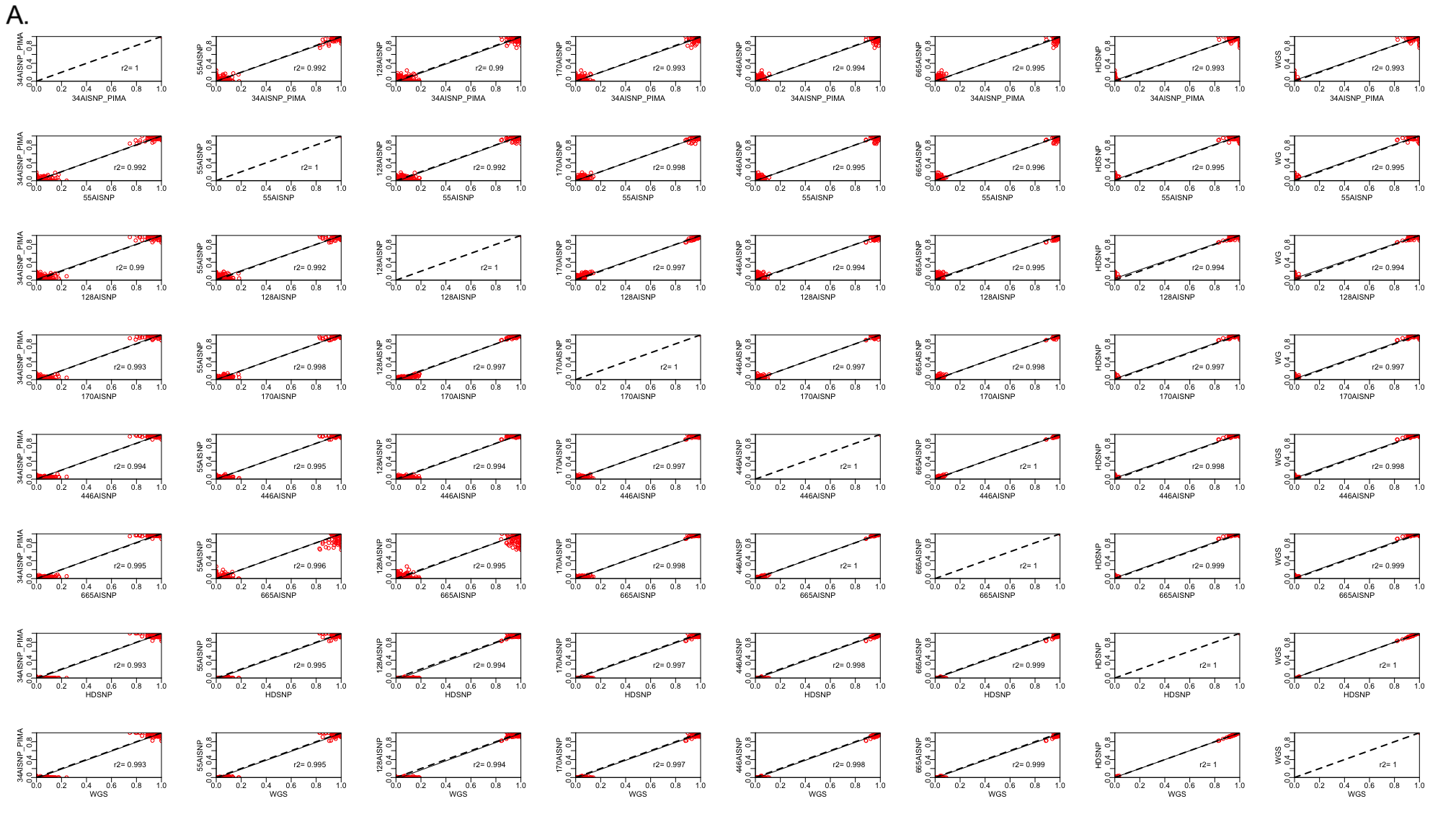


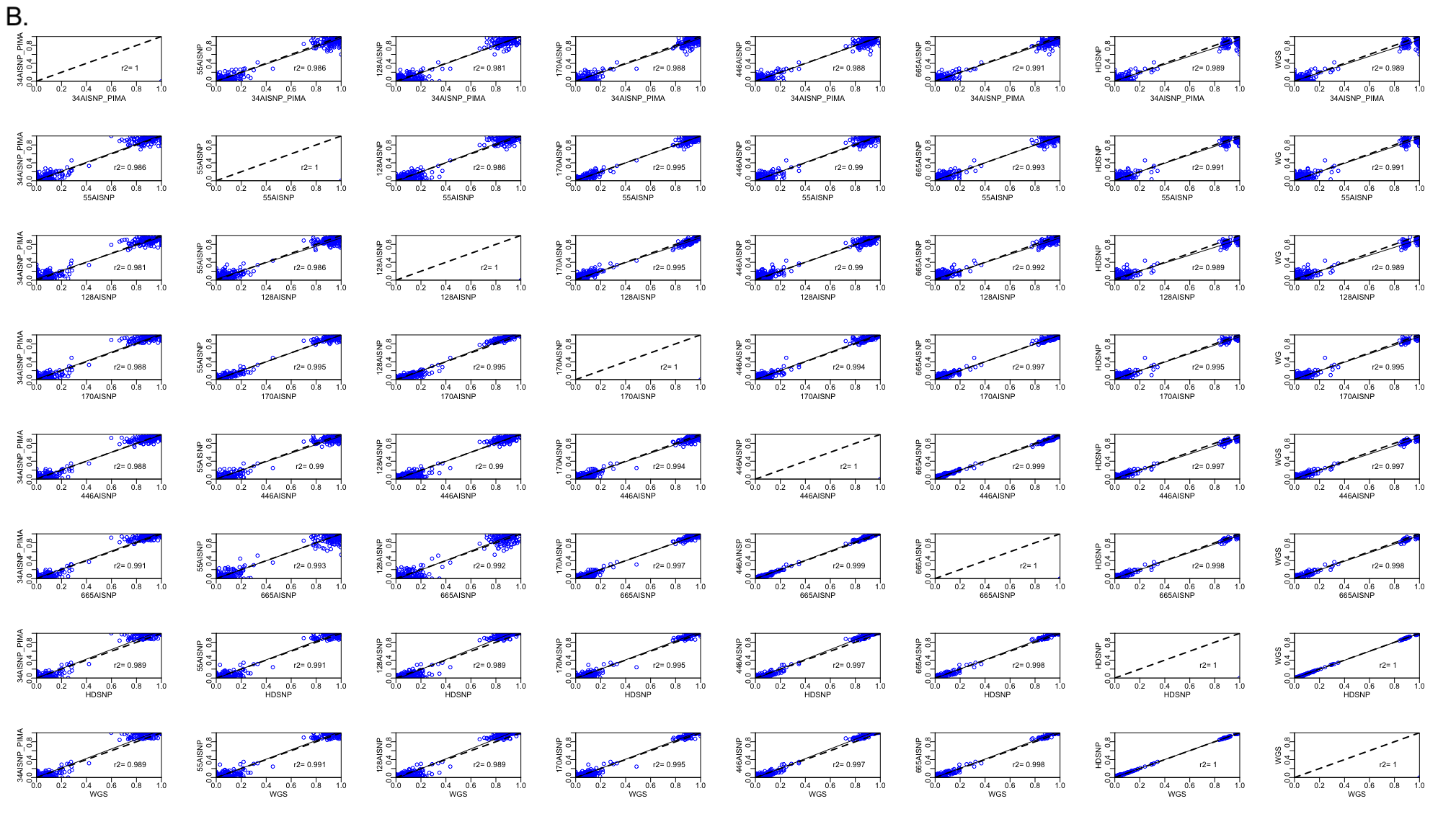


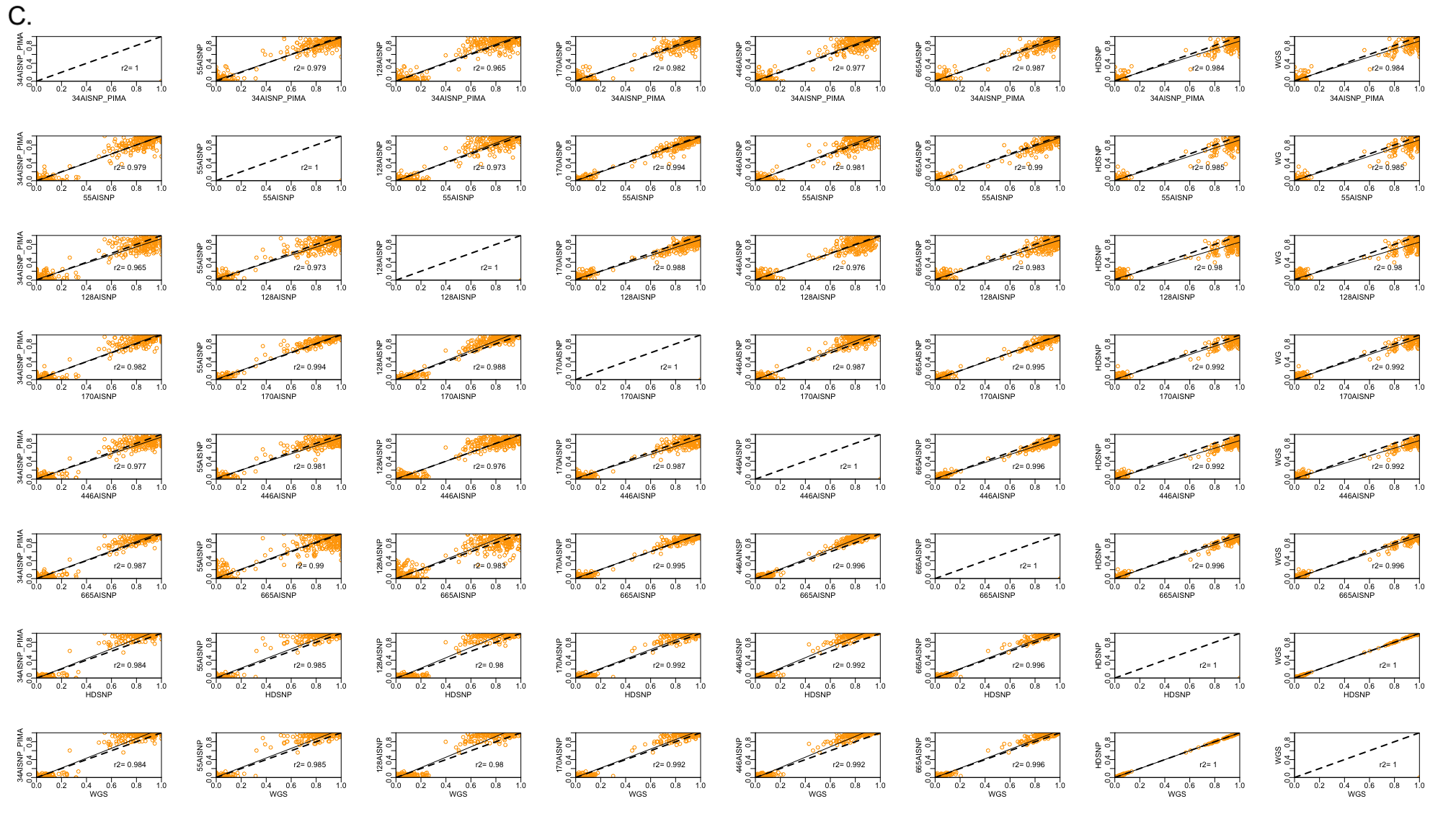


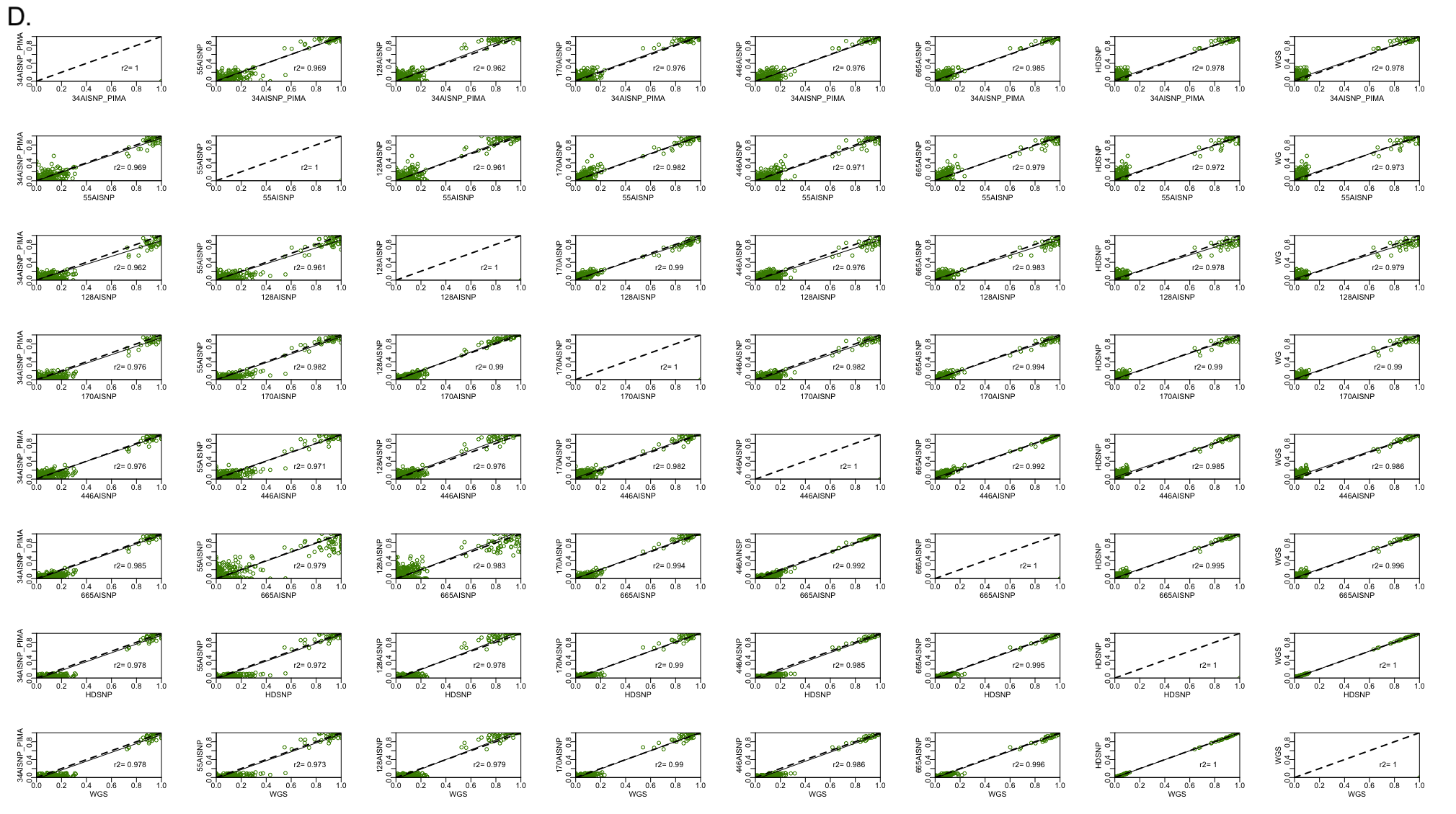


**
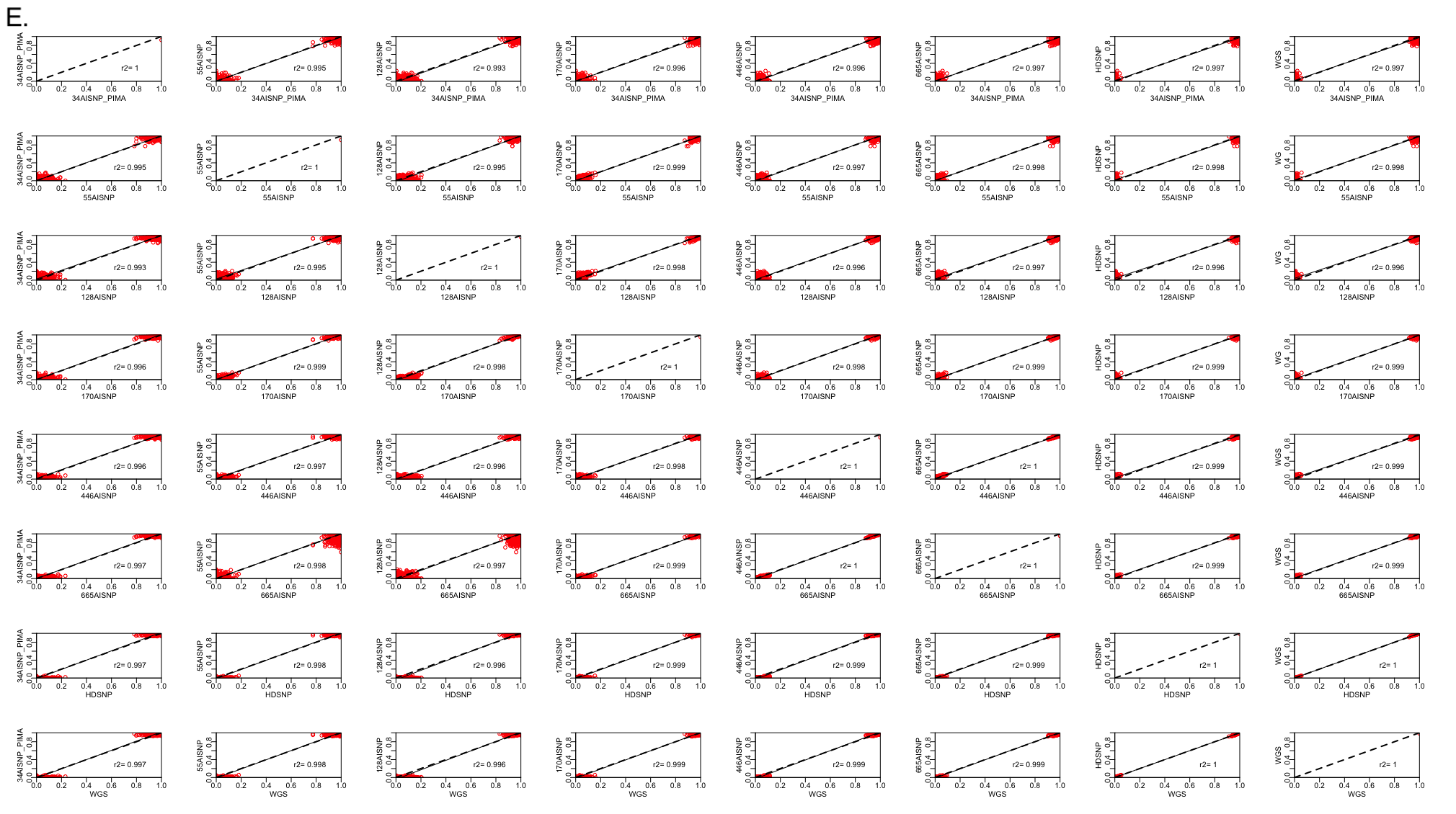
**

**
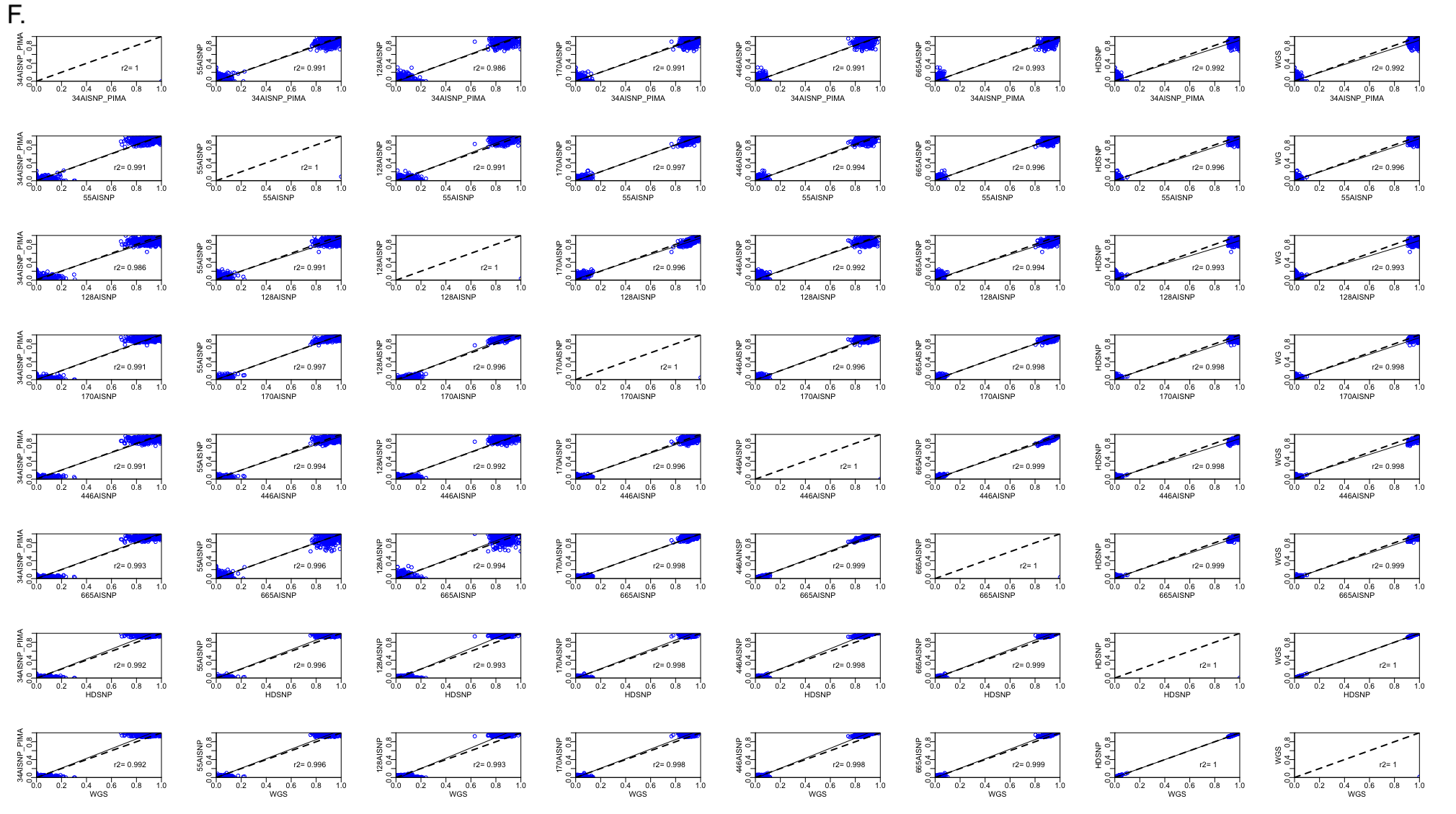
**

**
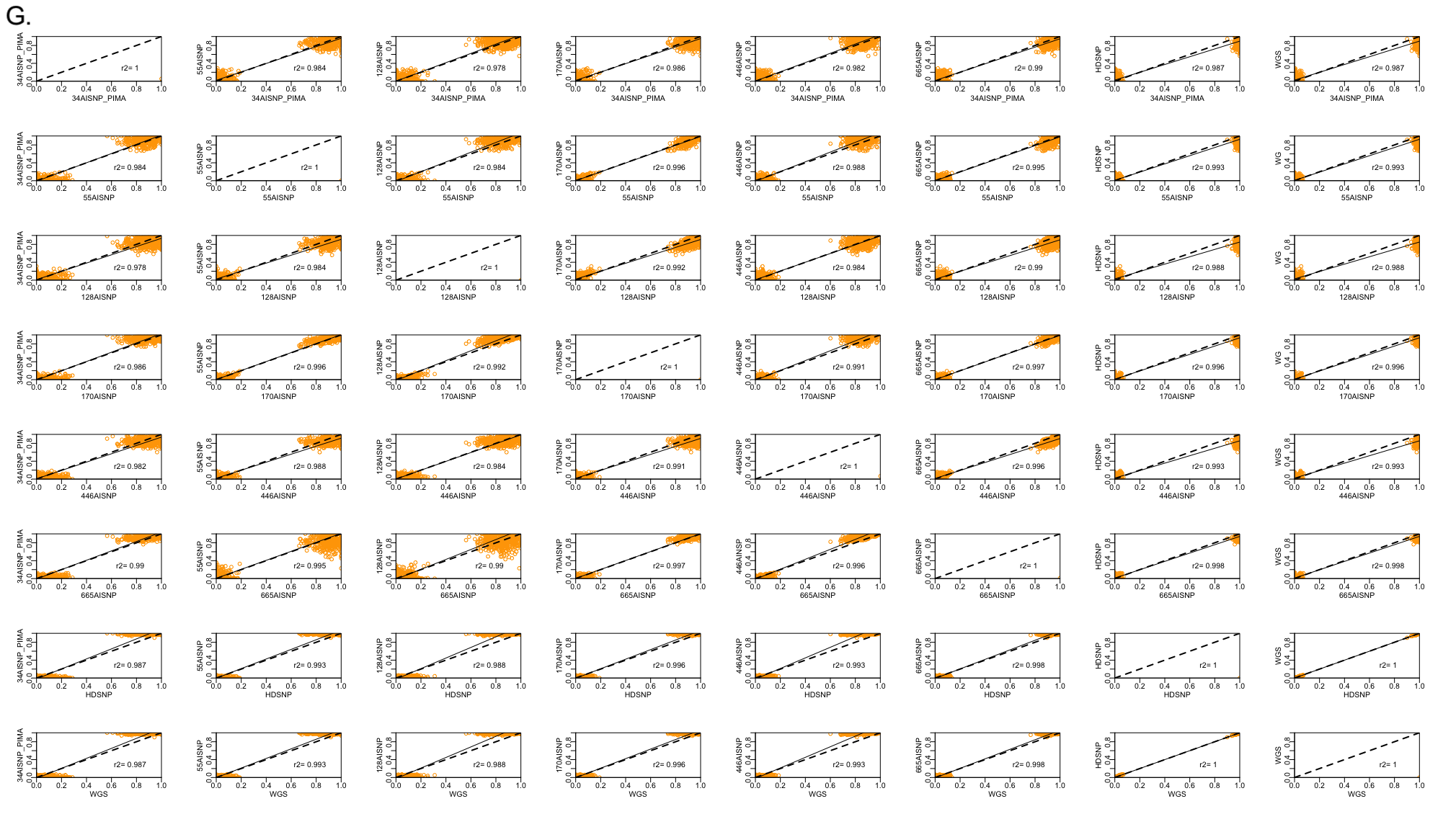
**

**Figure S1. Pairwise comparison of ancestry inferences for the parental WGS data with the 8 panel sets evaluated (34 AISNP +PIMA; 55 AISNP; 128 AISNP; 170 AISNP; 446 AISNP; 672 AISNP; HDSNP, WGS).** In the figure, r2 corresponds to the correlation coefficient, the black dashed line represents the trend, and the solid black line the perfect agreement between two panels. Samples: (A) HDGP-African (red); (B) HGDP-European (blue); (B) HGDP-East Asian (orange), and (D) HGDP-Native American (green); (E) 1KGP-African (red); (F) 1KGP-European (blue); (G) 1KGP-East Asian (orange).


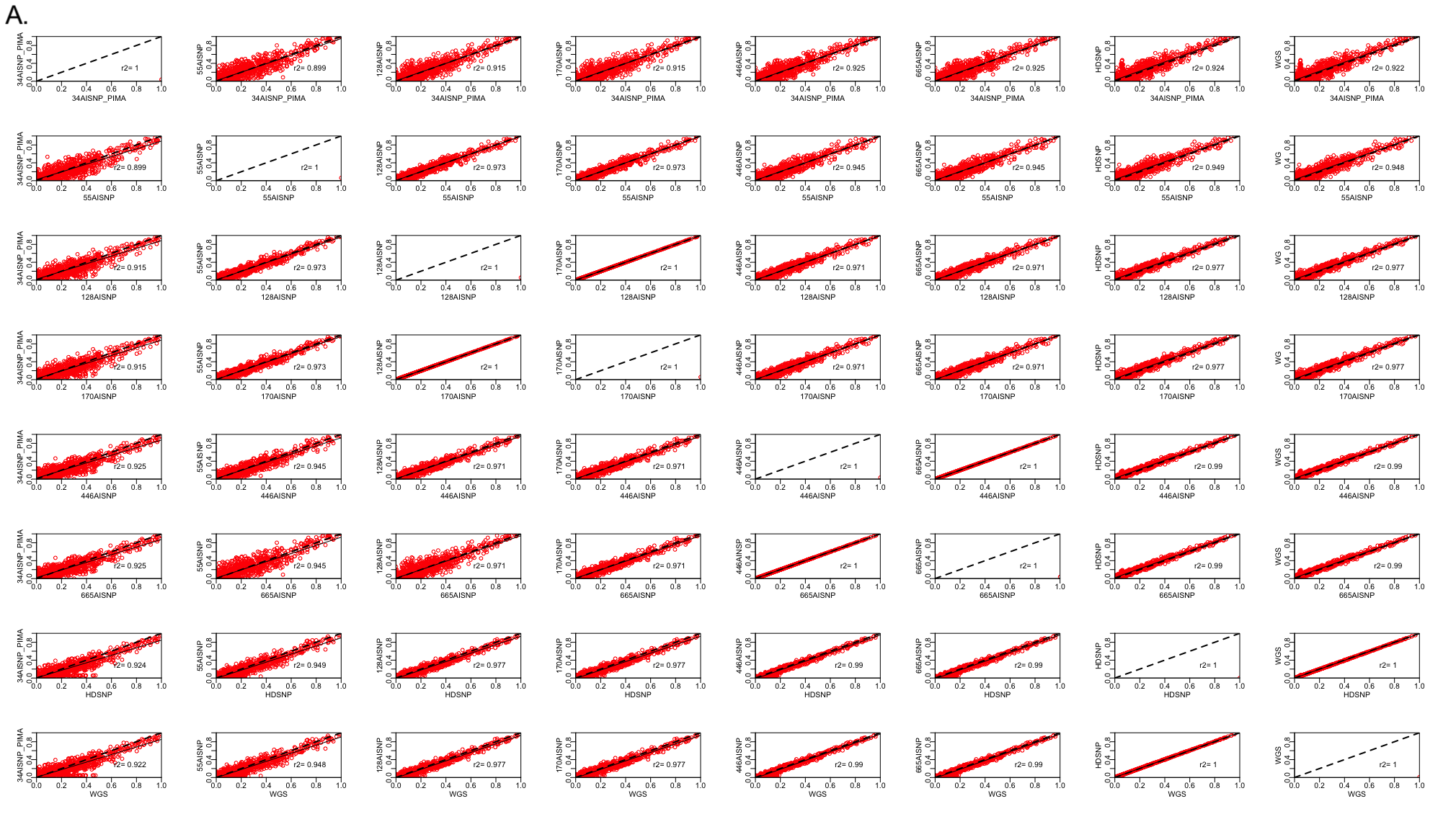


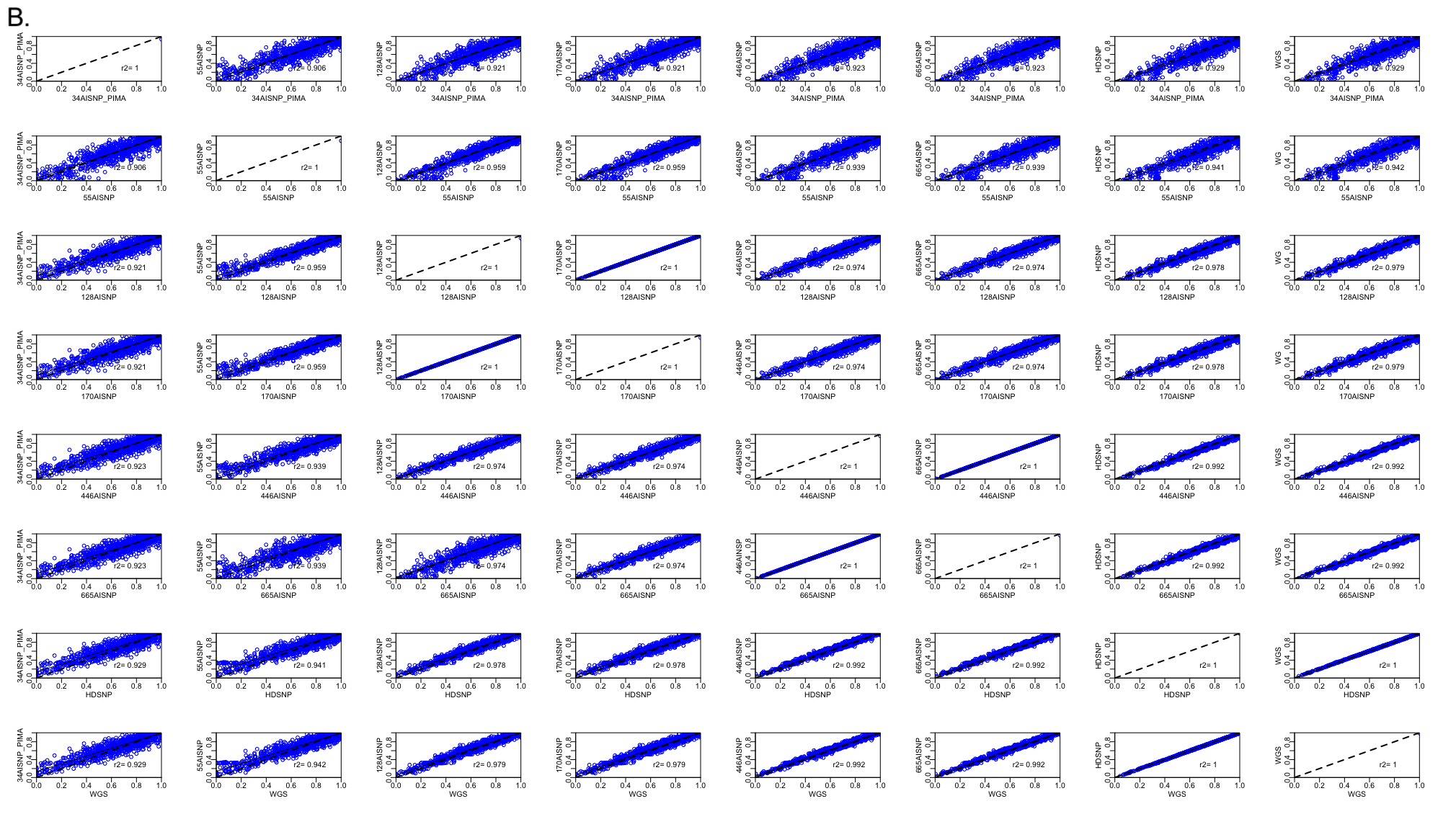


**
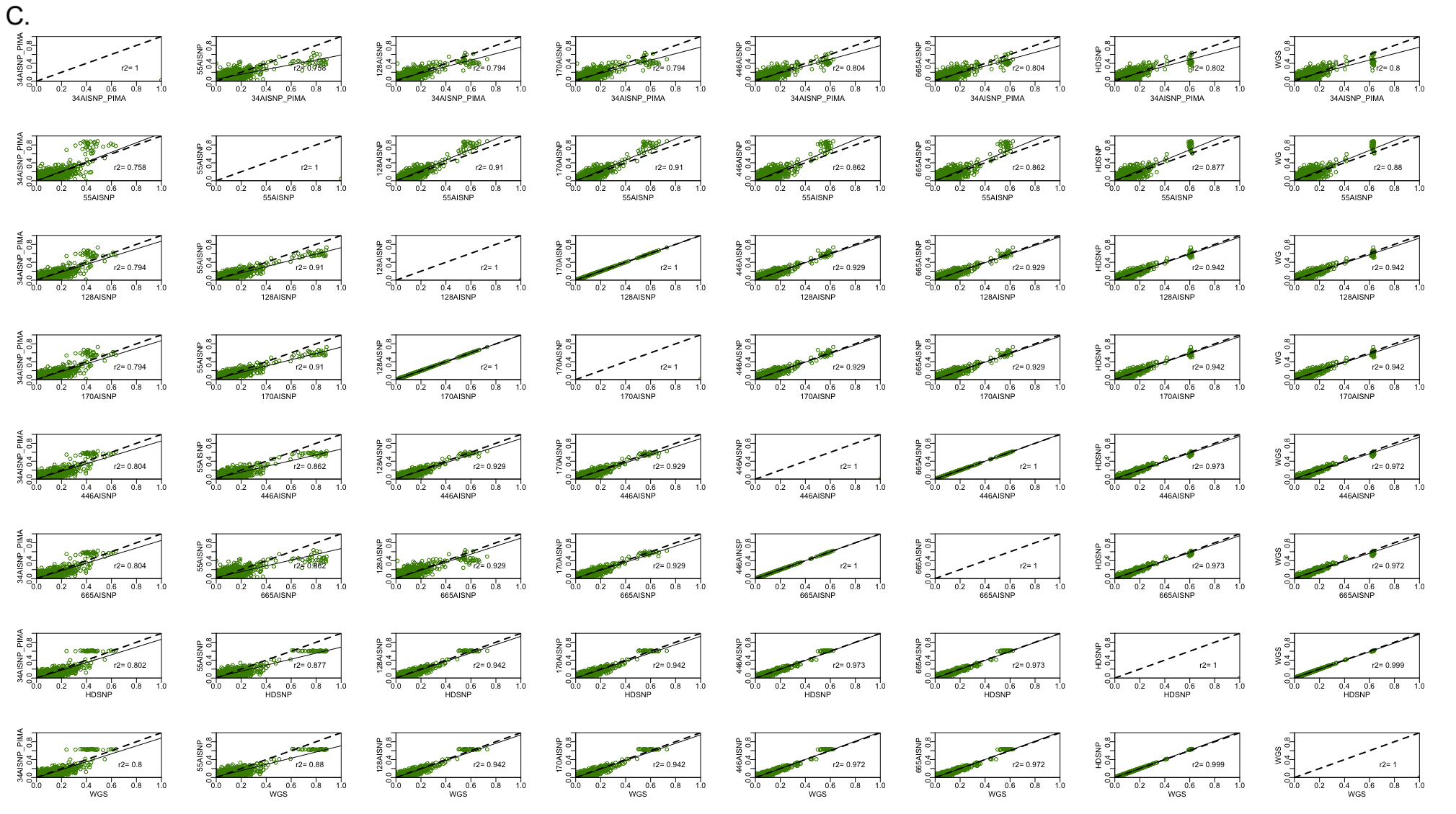
**


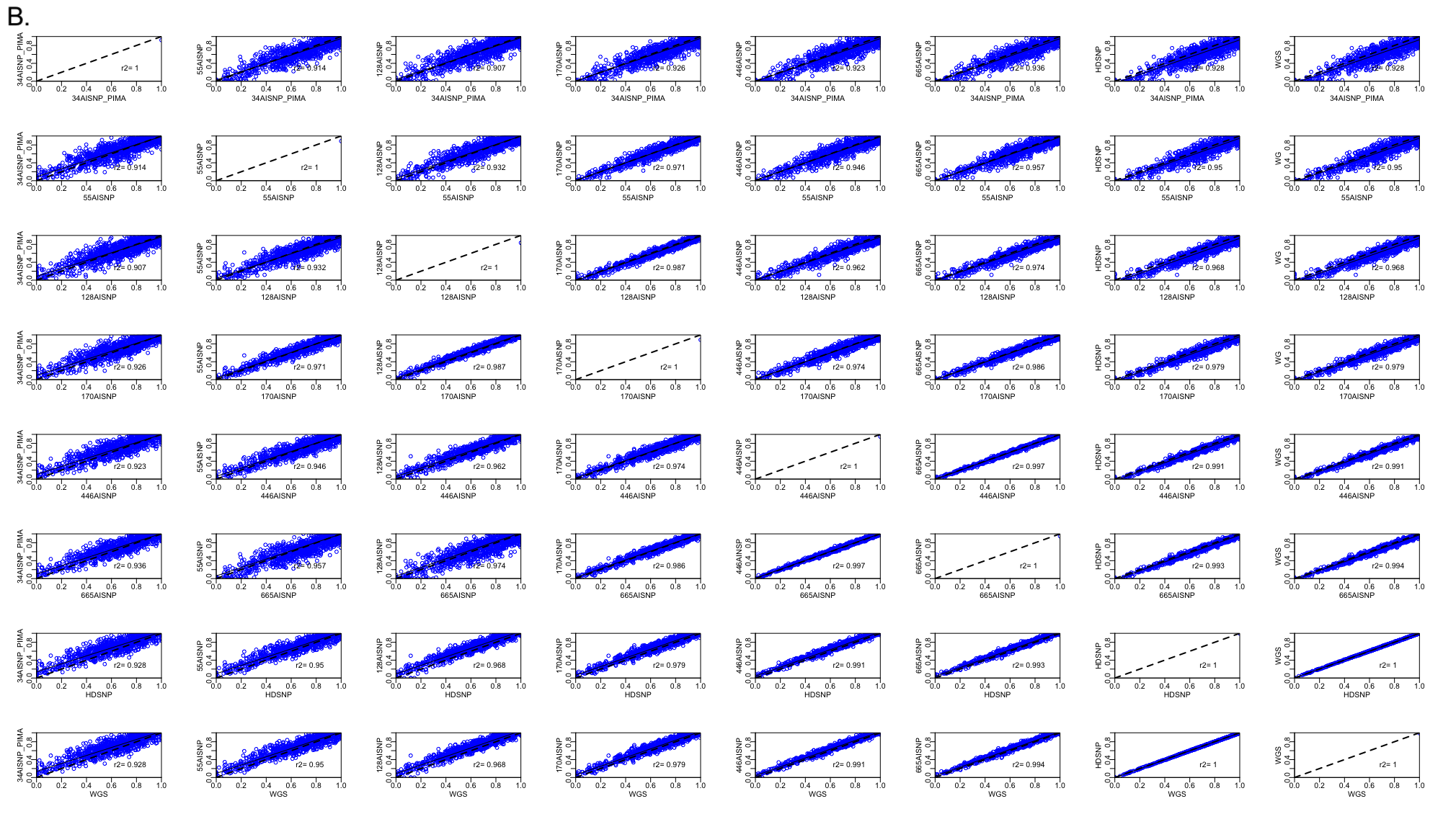
**Figure S2. Paired comparison of tri-hybrid model ancestry inferences for the Brazilian SABE samples with the 8 sets of panels evaluated (34 AISNP +PIMA; 55 AISNP; 128 AISNP; 170 AISNP; 446 AISNP; 672 AISNP; HDSNP, WGS).** In the figure, r2 corresponds to the correlation coefficient, the black dashed line represents the trend and the solid black line represents the perfect agreement between two panels. Components: (A) African descent (red); (B) European descent (blue) and (C) Native American descent (green).


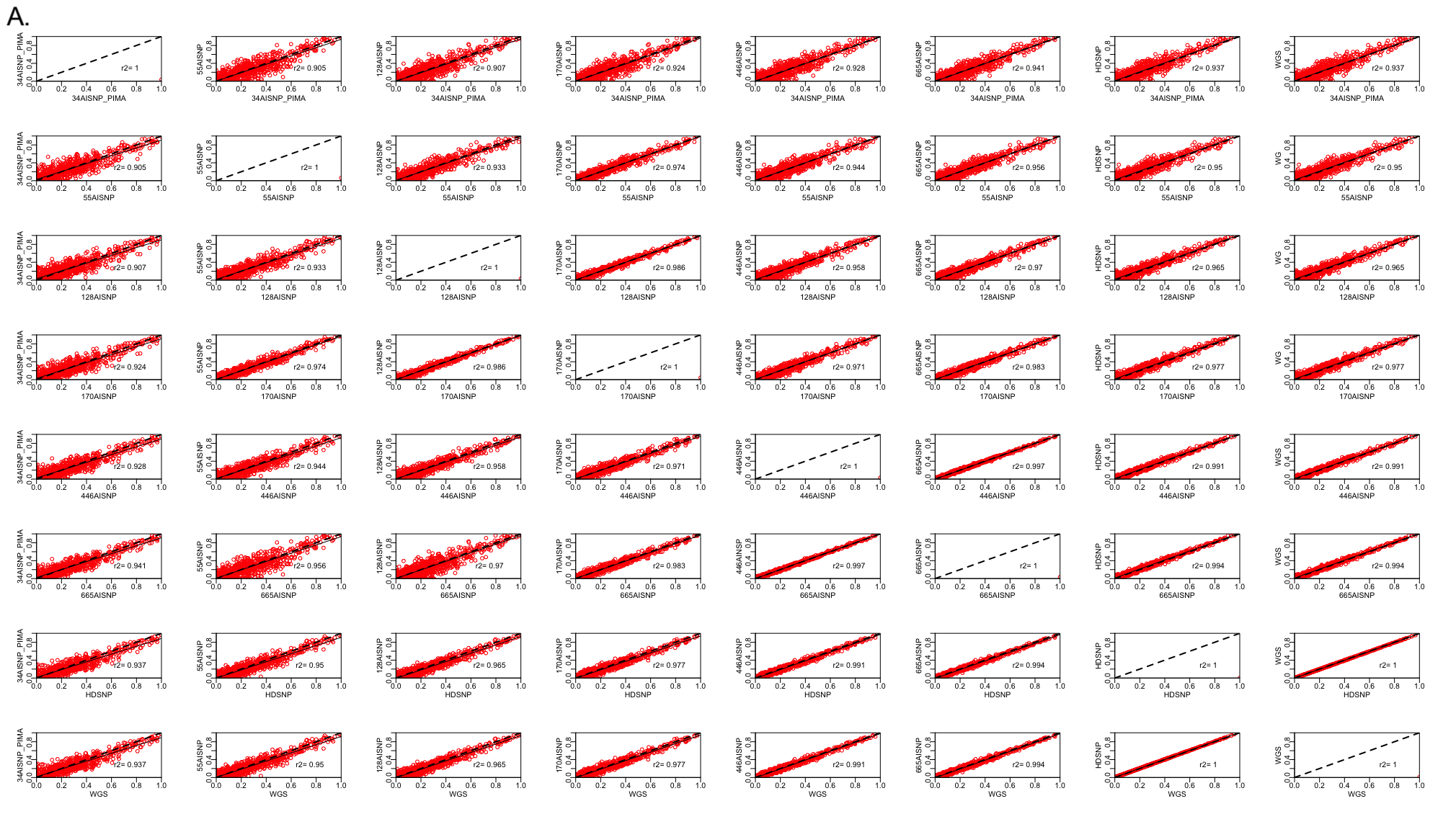


**
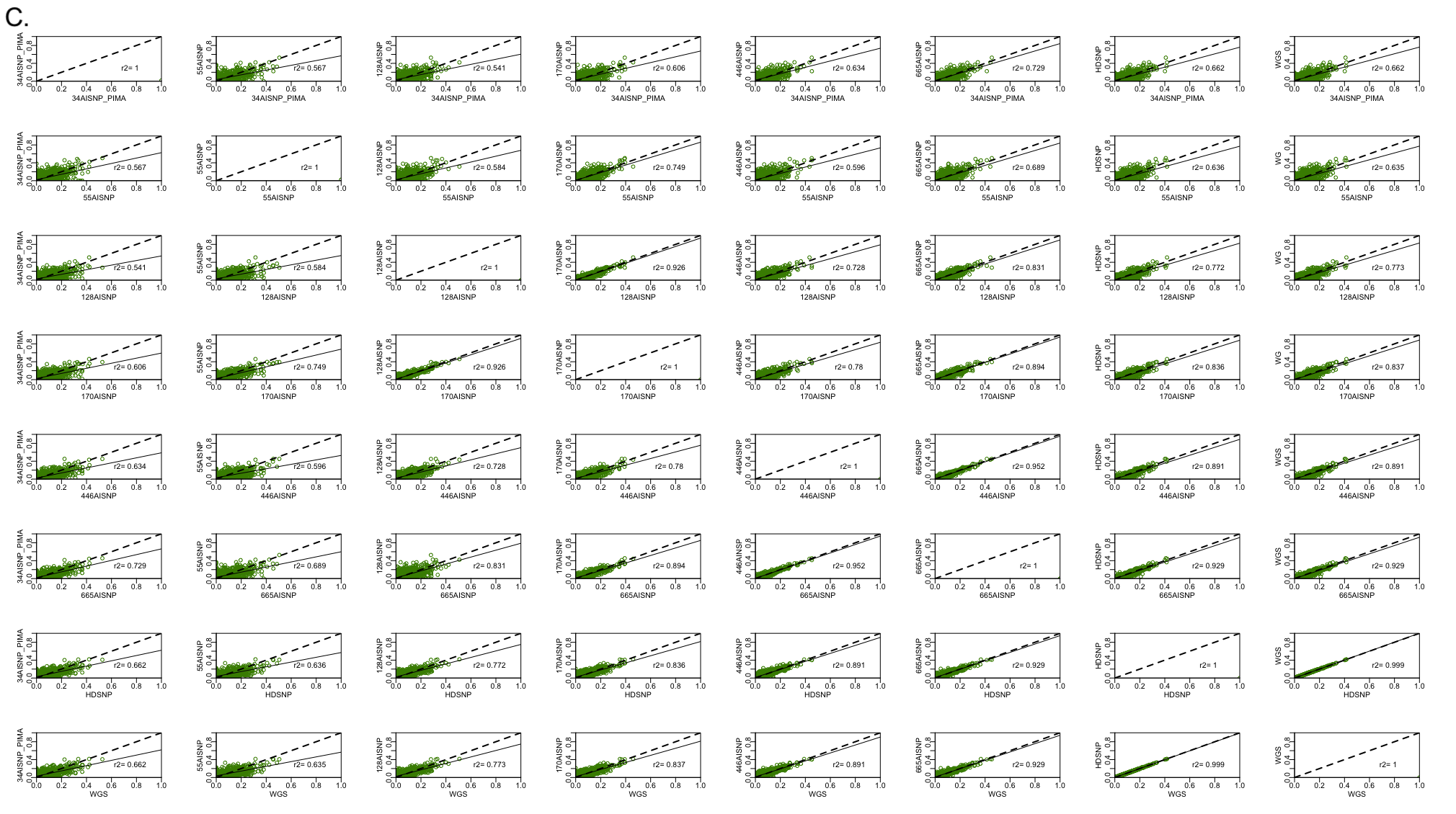
**

**
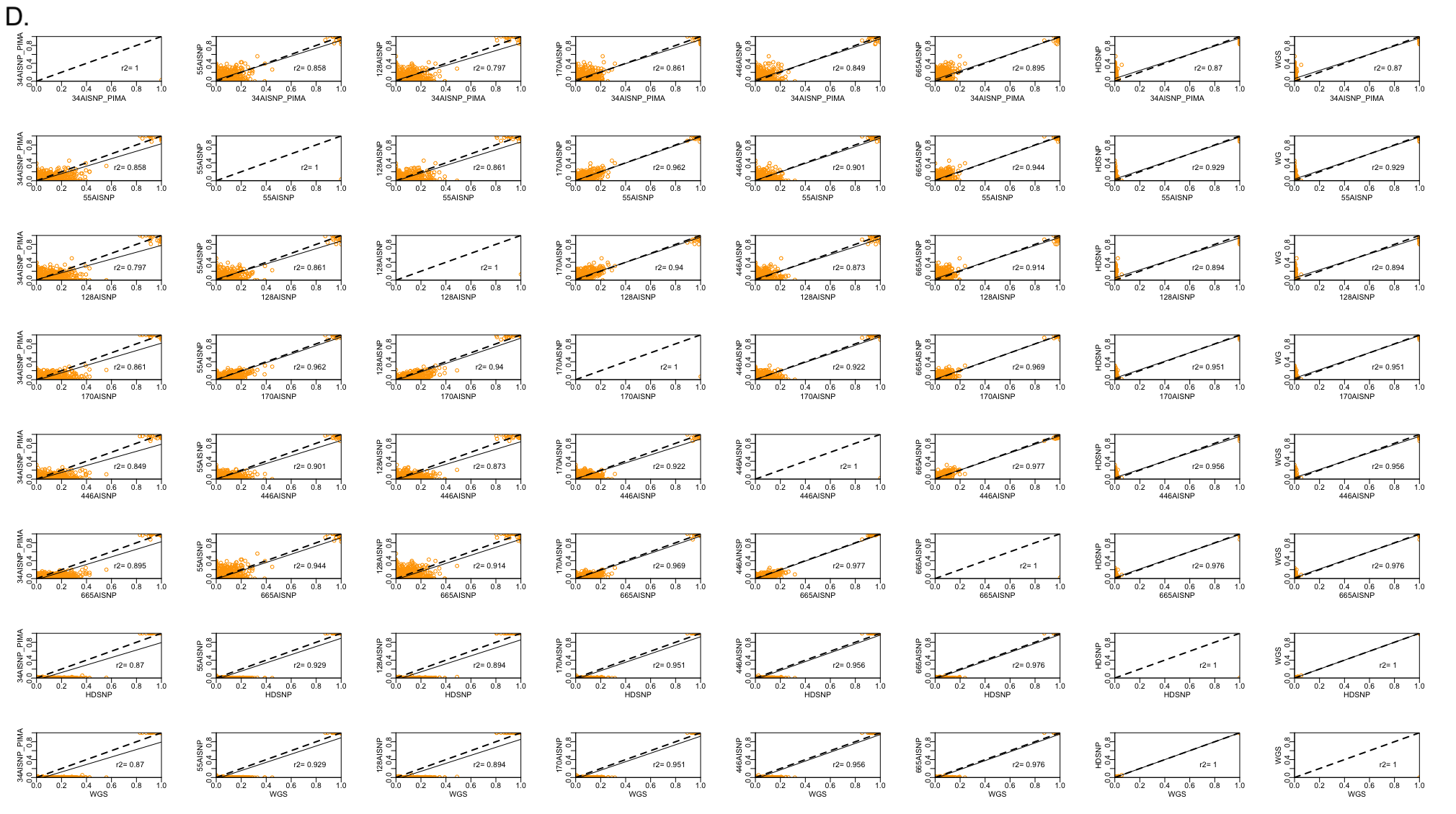
**

**Figure S3. Paired comparison of tetra-hybrid model ancestry inferences for the Brazilian SABE samples with the 8 sets of panels evaluated (34 AISNP +PIMA; 55 AISNP; 128 AISNP; 170 AISNP; 446 AISNP; 672 AISNP; HDSNP, WGS).** In the figure, r2 corresponds to the correlation coefficient, the black dashed line represents the trend and the solid black line represents the perfect agreement between two panels. Components: (A) African descent (red), (B) European descent (blue), (C) Native American descent (green) and (D) East Asian descent (orange)


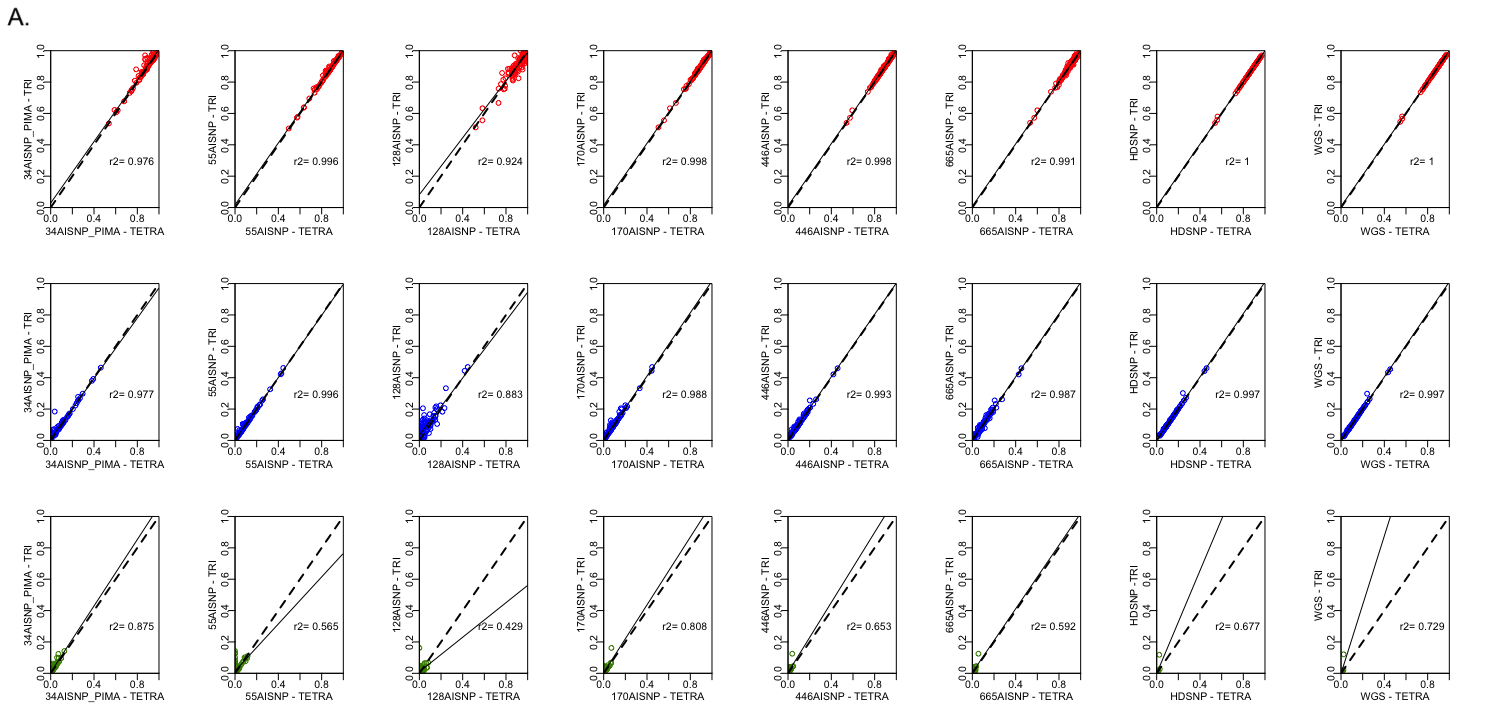


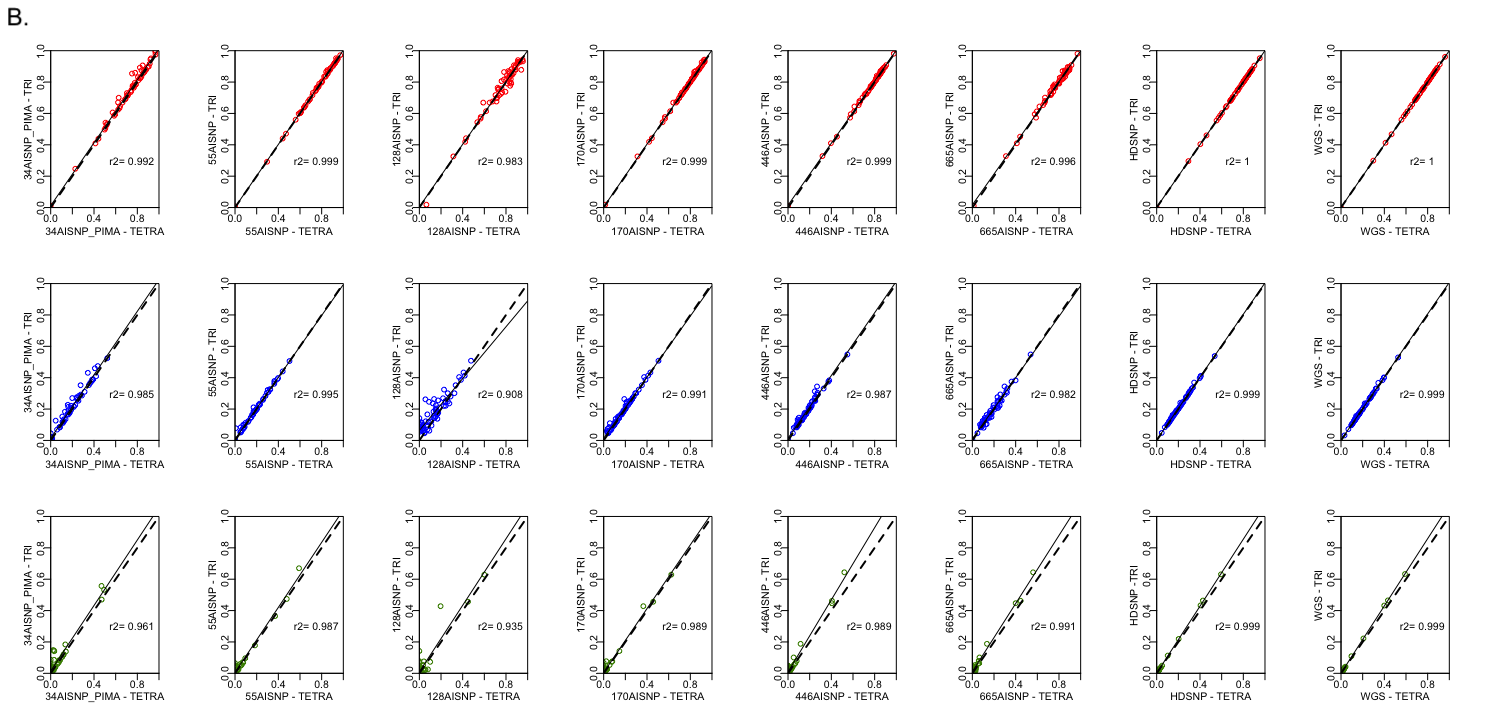


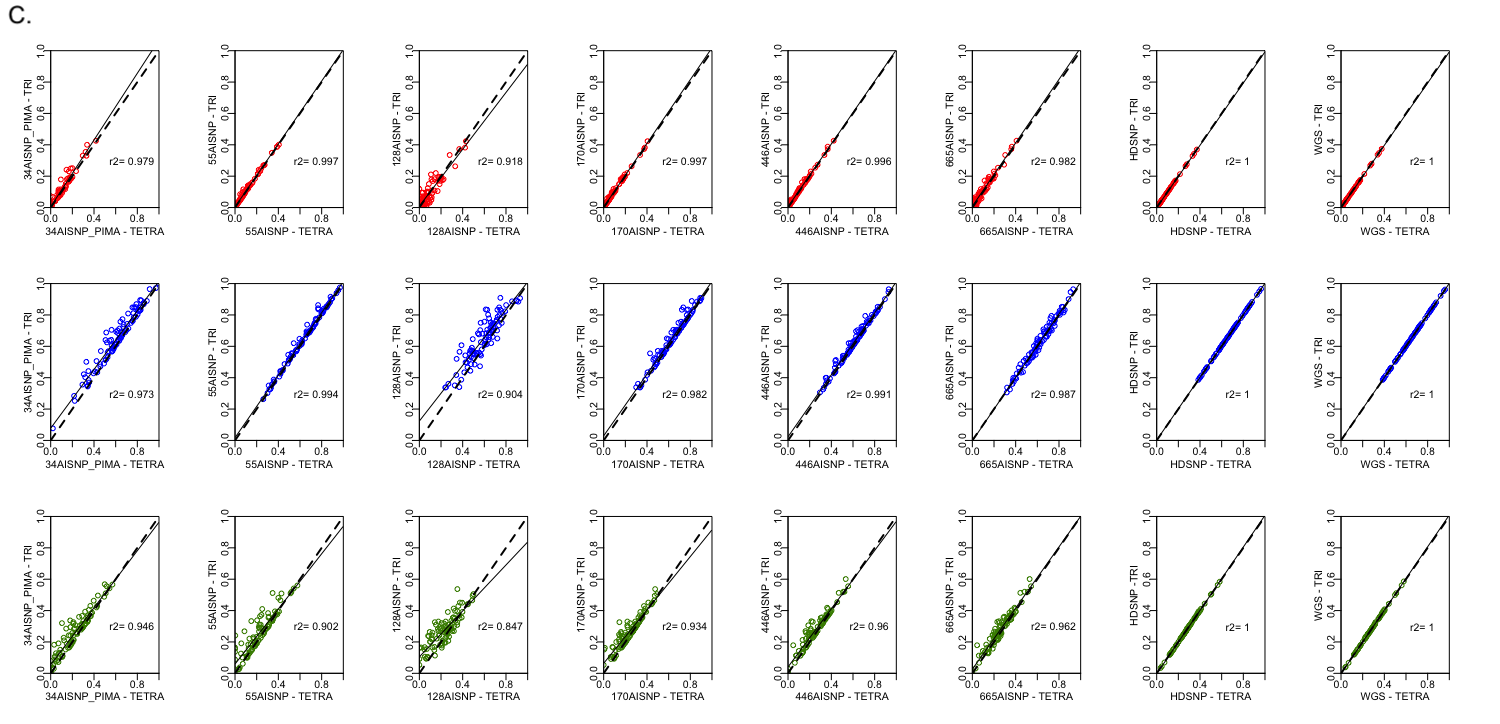


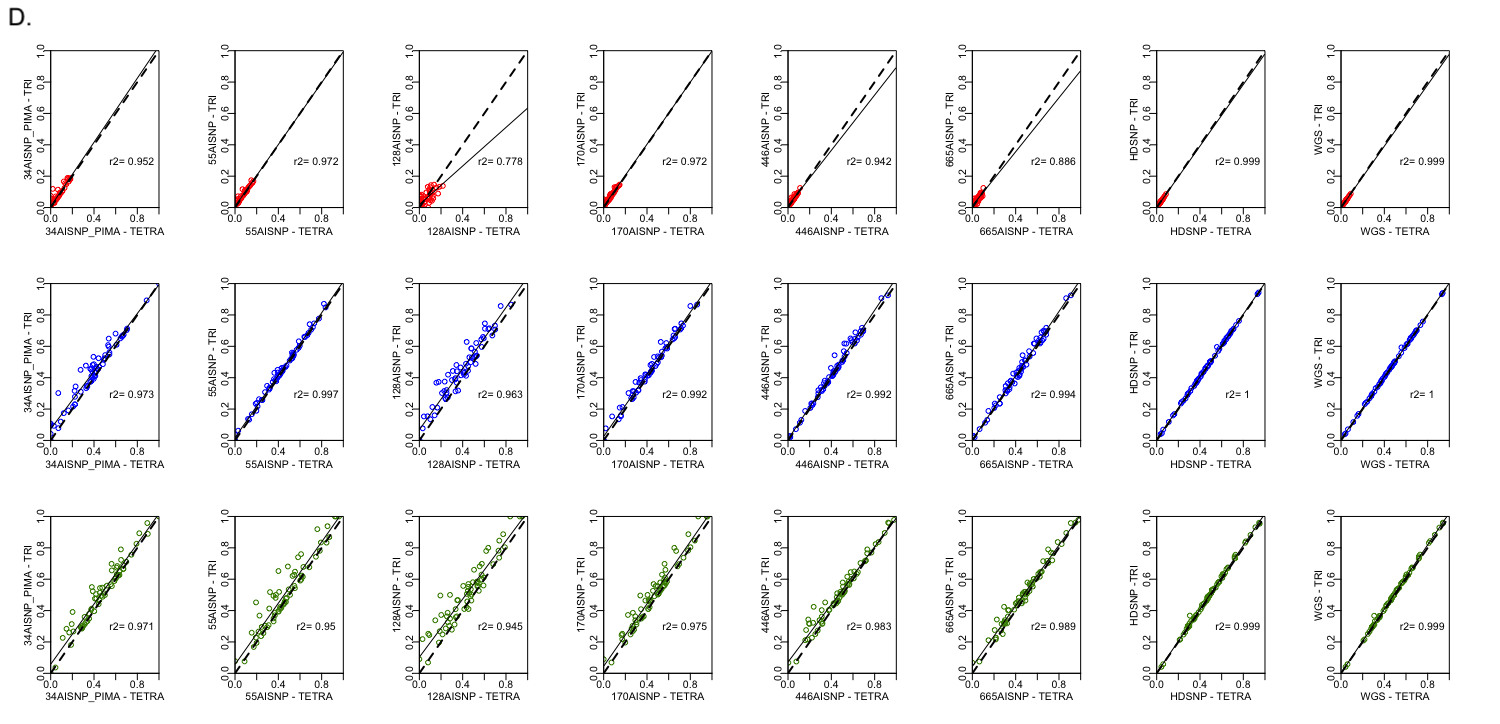


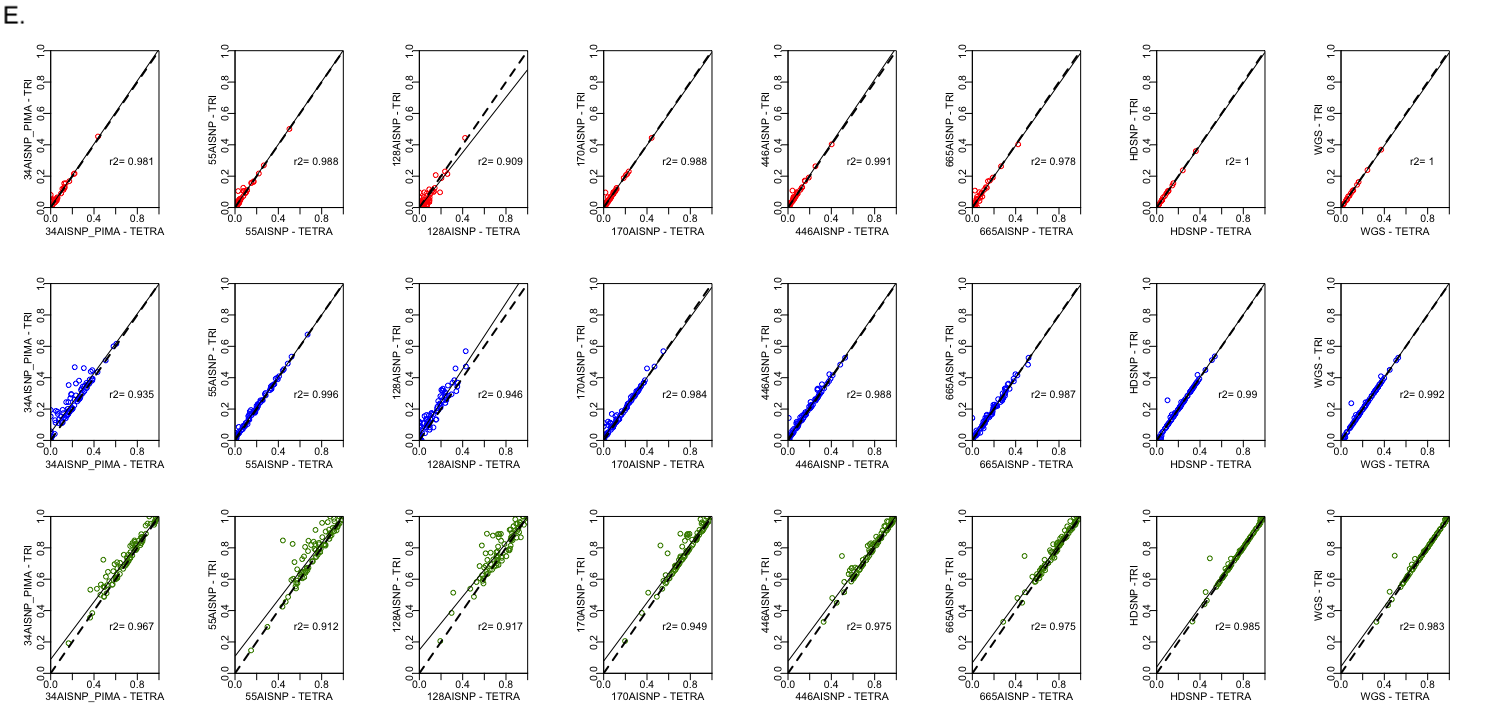


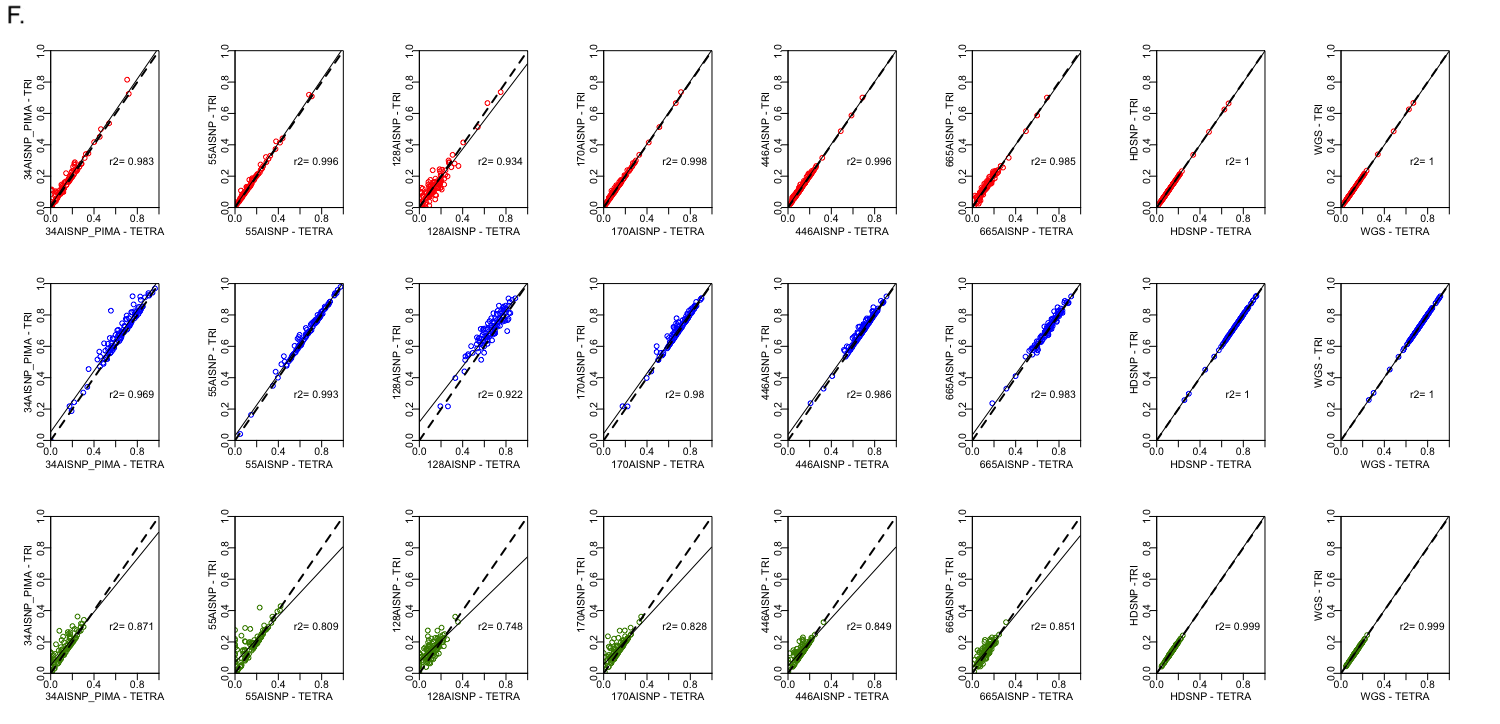


**Figure S4. Pairwise comparison of ancestry inferences by tri- and tetra-hybrid models for admixed samples (1KGP) with the 8 panel sets evaluated (34 AISNP +PIMA; 55 AISNP; 128 AISNP; 170 AISNP; 446 AISNP; 672 AISNP; HDSNP, WGS).** The x-axis corresponds to the ancestry inference by the tetra-hybrid model for a given panel. The y-axis corresponds to the inference of genetic ancestrality by the tri-hybrid model for a given panel. In the figure, r2 corresponds to the correlation coefficient, the black dashed line represents the trend, and the solid black line the perfect agreement between two panels. The red, blue and green colors correspond to the inference of African, European and Native American ancestral components, respectively. (A) corresponds to the Afro-Caribbean, (B) Afro-American, (C) Colombian, (D) Mexican, (E) Peruvian and (F) Puerto Rican.


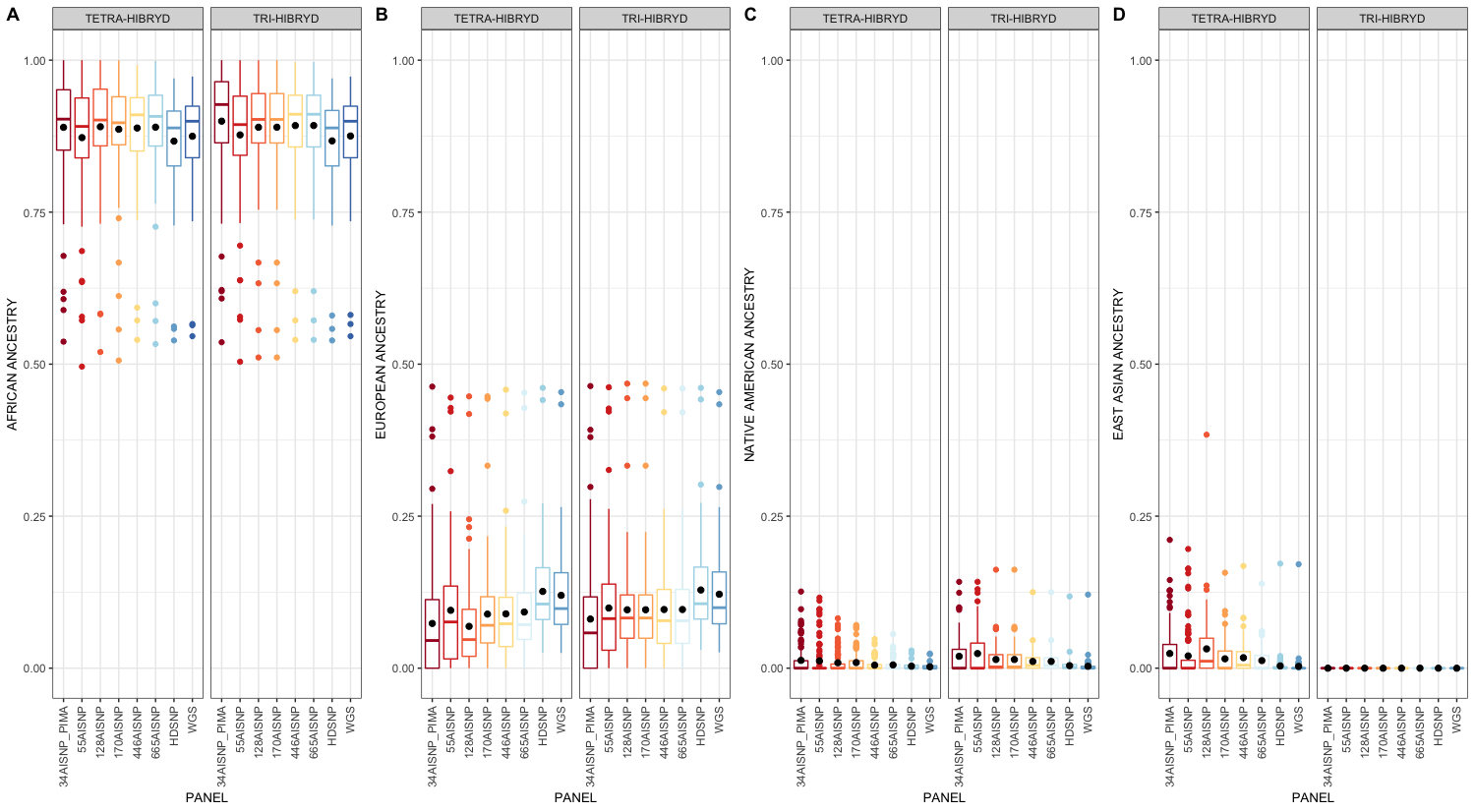


**Figure S5.** **Pairwise comparison of ancestry inferences by tri- and tetra-hybrid models for Afro-Caribbean samples (ACB) with the 8 panel sets evaluated (34 AISNP +PIMA; 55 AISNP; 128 AISNP; 170 AISNP; 446 AISNP; 672 AISNP; HDSNP, WGS).** The x-axis corresponds to the ancestry inference by the tetra-hybrid model for a given panel. The y-axis corresponds to the inference of genetic ancestrality by the tri-hybrid model for a given panel. In the figure, r2 corresponds to the correlation coefficient, the black dashed line represents the trend, and the solid black line the perfect agreement between two panels. The red, blue and green colors correspond to the inference of African, European and Native American ancestral components, respectively.


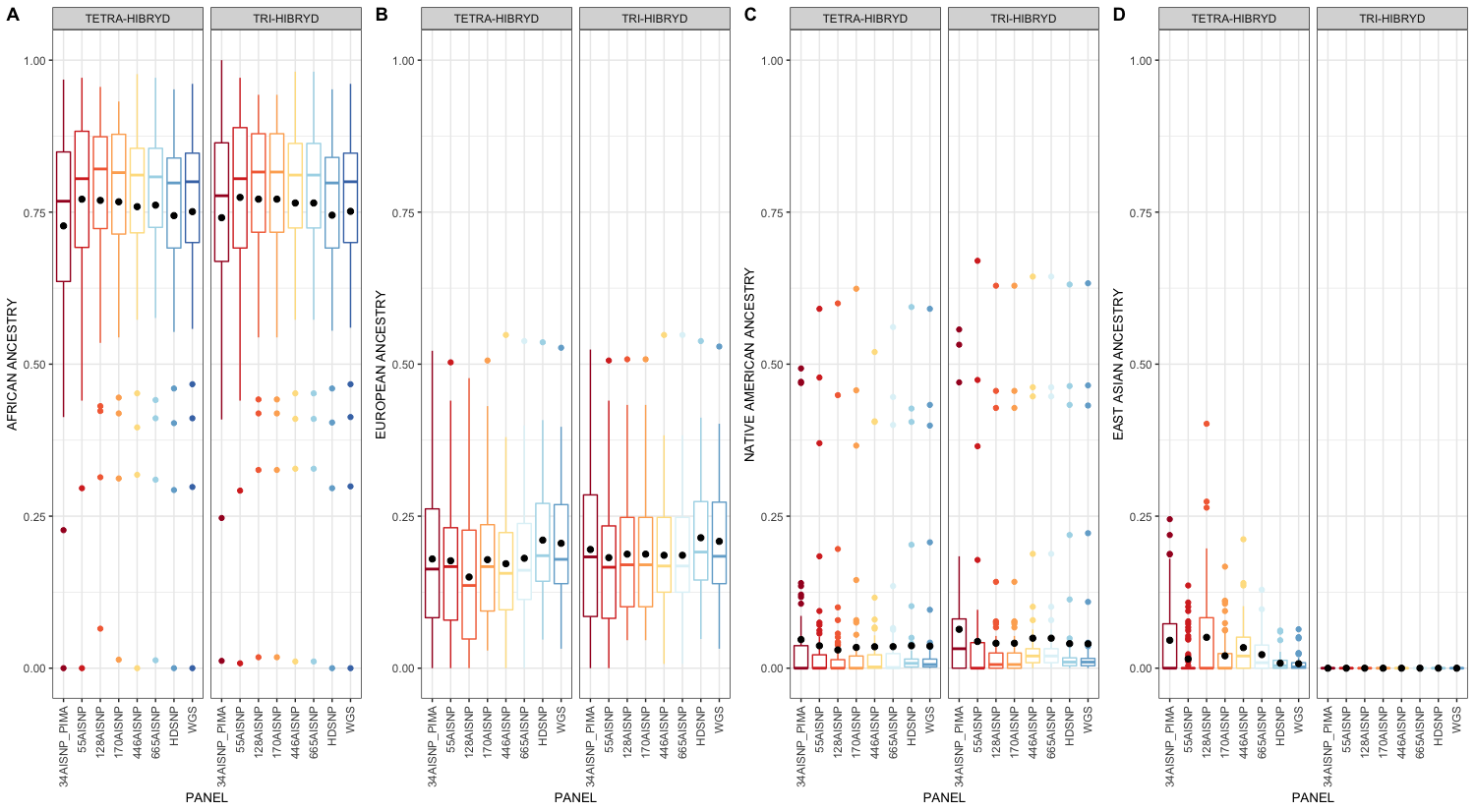


**Figure S6.** **Pairwise comparison of ancestry inferences by tri- and tetra-hybrid models for Afro-American samples (ASW) with the 8 panel sets evaluated (34 AISNP +PIMA; 55 AISNP; 128 AISNP; 170 AISNP; 446 AISNP; 672 AISNP; HDSNP, WGS).** The x-axis corresponds to the ancestry inference by the tetra-hybrid model for a given panel. The y-axis corresponds to the inference of genetic ancestrality by the tri-hybrid model for a given panel. In the figure, r2 corresponds to the correlation coefficient, the black dashed line represents the trend, and the solid black line the perfect agreement between two panels. The red, blue and green colors correspond to the inference of African, European and Native American ancestral components, respectively.


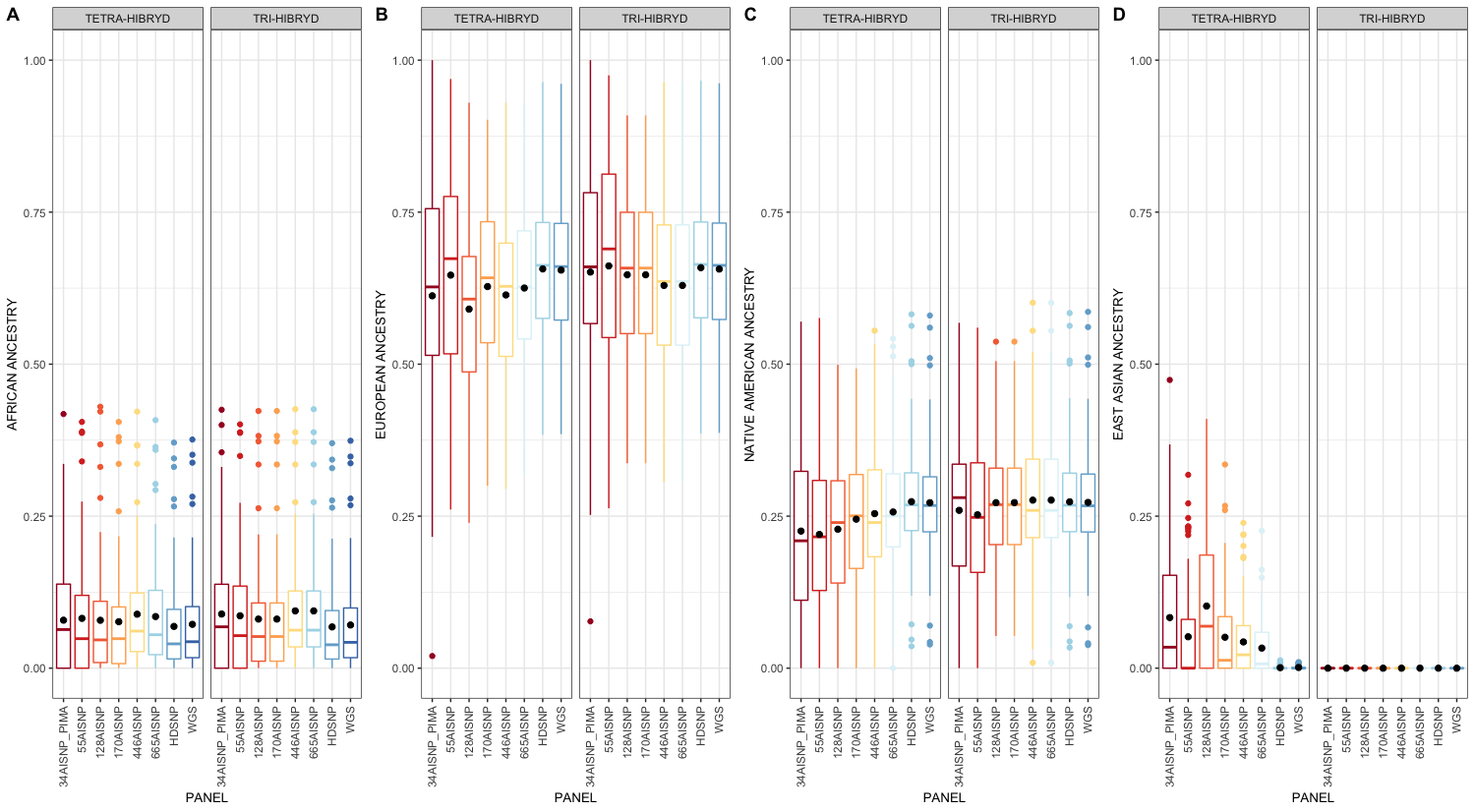


**Figure S7.** **Pairwise comparison of ancestry inferences by tri- and tetra-hybrid models for Colombian samples (CLM) with the 8 panel sets evaluated (34 AISNP +PIMA; 55 AISNP; 128 AISNP; 170 AISNP; 446 AISNP; 672 AISNP; HDSNP, WGS).** The x-axis corresponds to the ancestry inference by the tetra-hybrid model for a given panel. The y-axis corresponds to the inference of genetic ancestrality by the tri-hybrid model for a given panel. In the figure, r2 corresponds to the correlation coefficient, the black dashed line represents the trend, and the solid black line the perfect agreement between two panels. The red, blue and green colors correspond to the inference of African, European and Native American ancestral components, respectively.


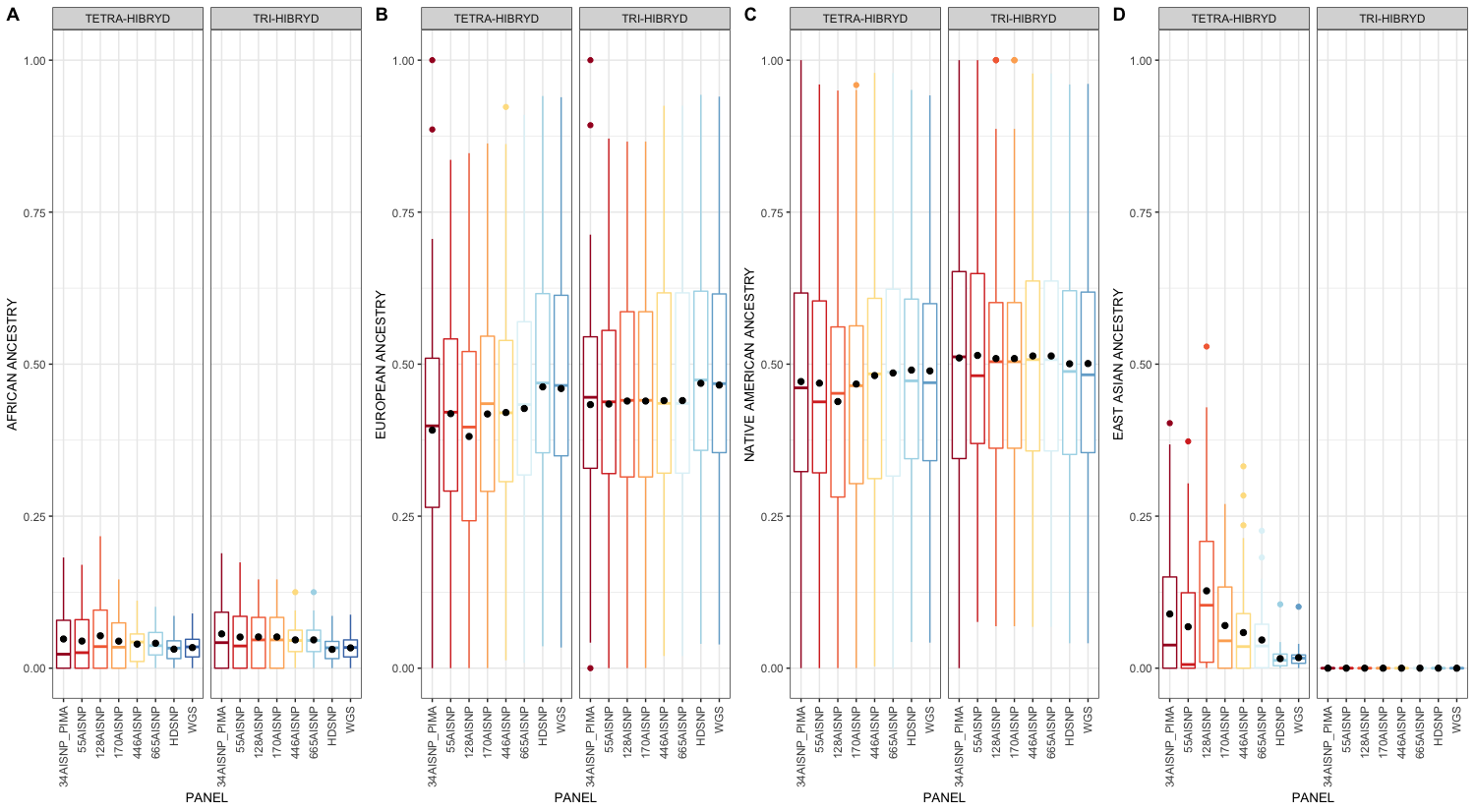


**Figure S8.** **Pairwise comparison of ancestry inferences by tri- and tetra-hybrid models for Mexican samples (MXL) with the 8 panel sets evaluated (34 AISNP +PIMA; 55 AISNP; 128 AISNP; 170 AISNP; 446 AISNP; 672 AISNP; HDSNP, WGS).** The x-axis corresponds to the ancestry inference by the tetra-hybrid model for a given panel. The y-axis corresponds to the inference of genetic ancestrality by the tri-hybrid model for a given panel. In the figure, r2 corresponds to the correlation coefficient, the black dashed line represents the trend, and the solid black line the perfect agreement between two panels. The red, blue and green colors correspond to the inference of African, European and Native American ancestral components, respectively.


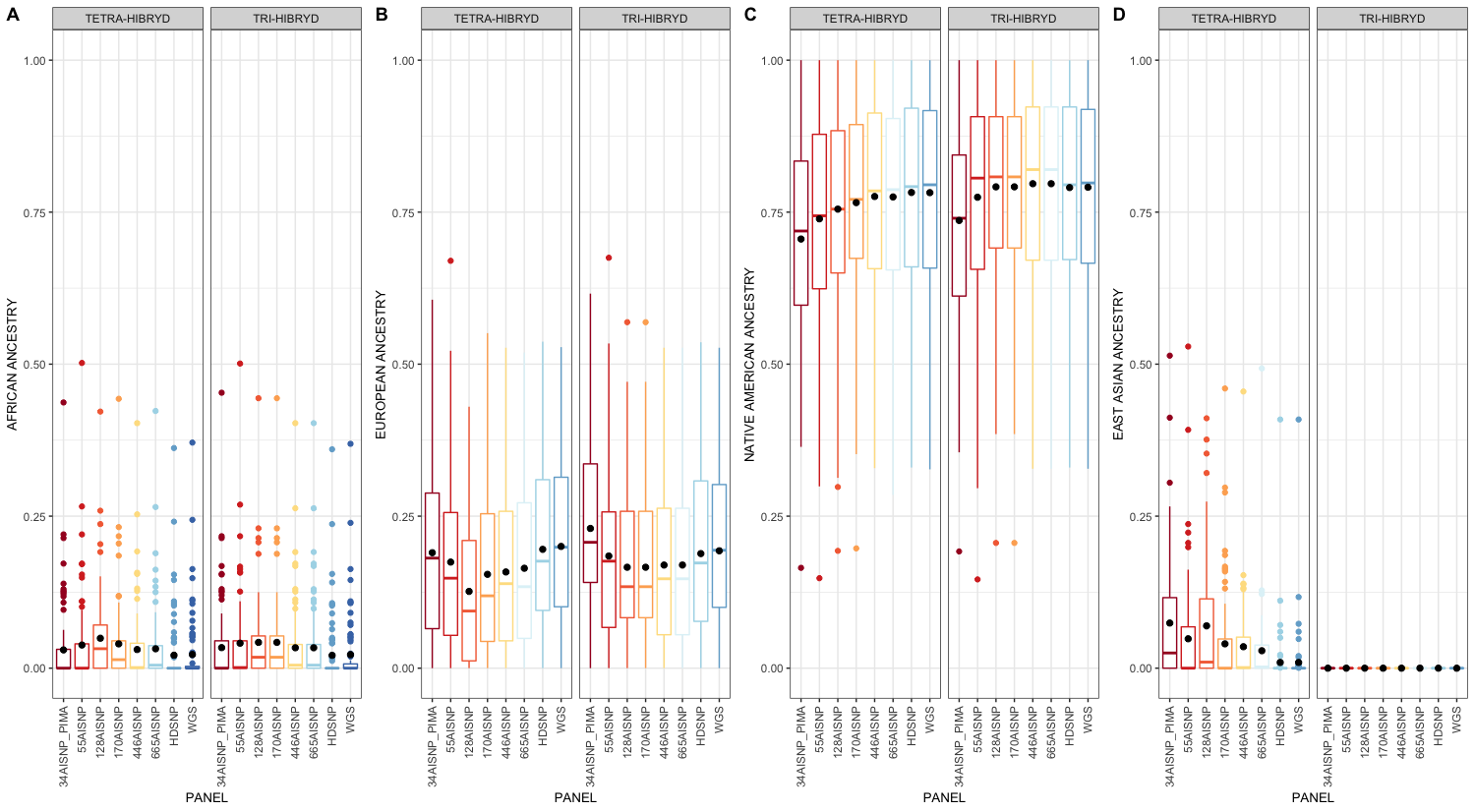


**Figure S9.** **Pairwise comparison of ancestry inferences by tri- and tetra-hybrid models for Peruvian samples (PEL) with the 8 panel sets evaluated (34 AISNP +PIMA; 55 AISNP; 128 AISNP; 170 AISNP; 446 AISNP; 672 AISNP; HDSNP, WGS).** The x-axis corresponds to the ancestry inference by the tetra-hybrid model for a given panel. The y-axis corresponds to the inference of genetic ancestrality by the tri-hybrid model for a given panel. In the figure, r2 corresponds to the correlation coefficient, the black dashed line represents the trend, and the solid black line the perfect agreement between two panels. The red, blue and green colors correspond to the inference of African, European and Native American ancestral components, respectively.


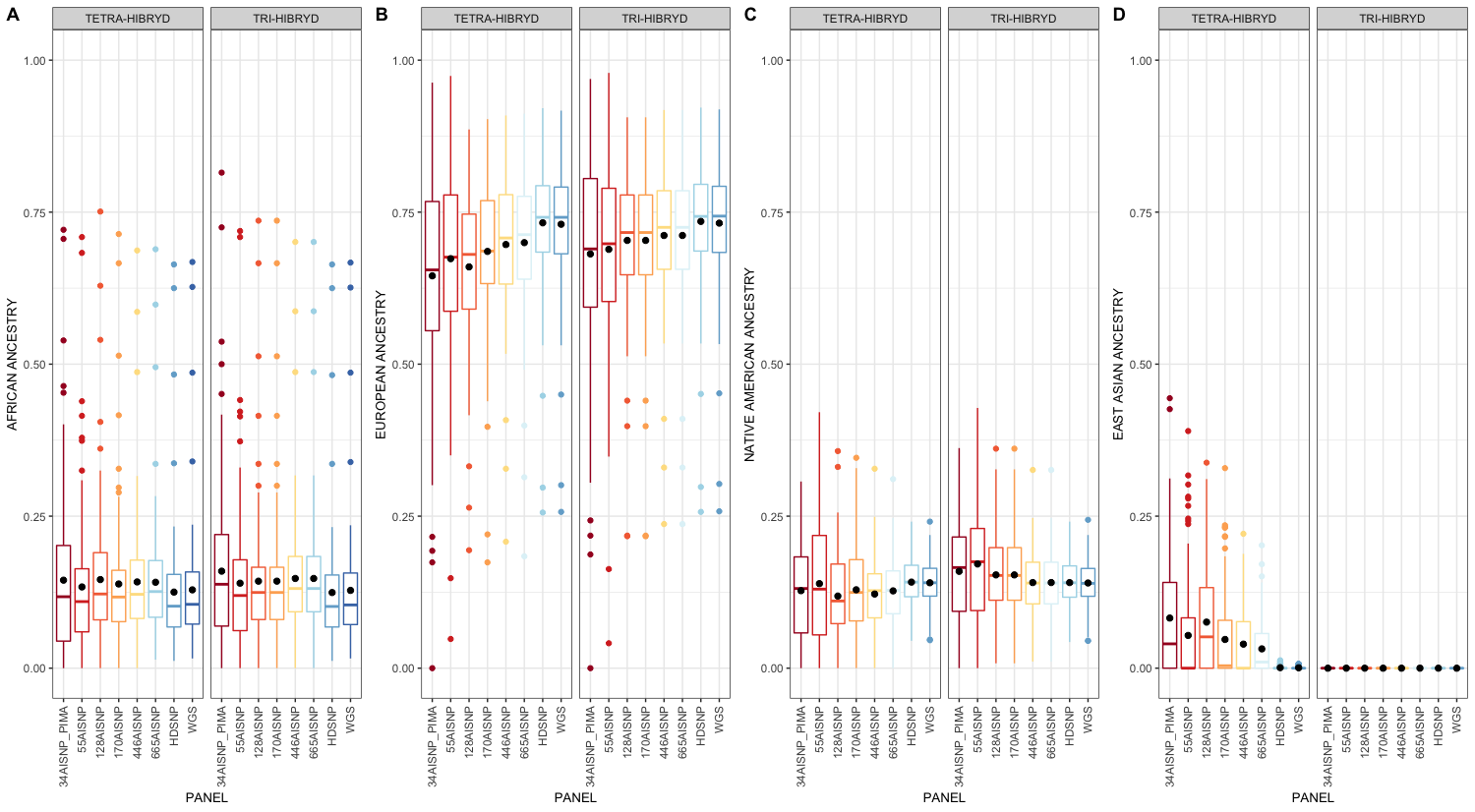


**Figure S10.** **Pairwise comparison of ancestry inferences by tri- and tetra-hybrid models for Puerto Rican samples (PUR) with the 8 panel sets evaluated (34 AISNP +PIMA; 55 AISNP; 128 AISNP; 170 AISNP; 446 AISNP; 672 AISNP; HDSNP, WGS).** The x-axis corresponds to the ancestry inference by the tetra-hybrid model for a given panel. The y-axis corresponds to the inference of genetic ancestrality by the tri-hybrid model for a given panel. In the figure, r2 corresponds to the correlation coefficient, the black dashed line represents the trend, and the solid black line the perfect agreement between two panels. The red, blue and green colors correspond to the inference of African, European and Native American ancestral componentes, respectively.


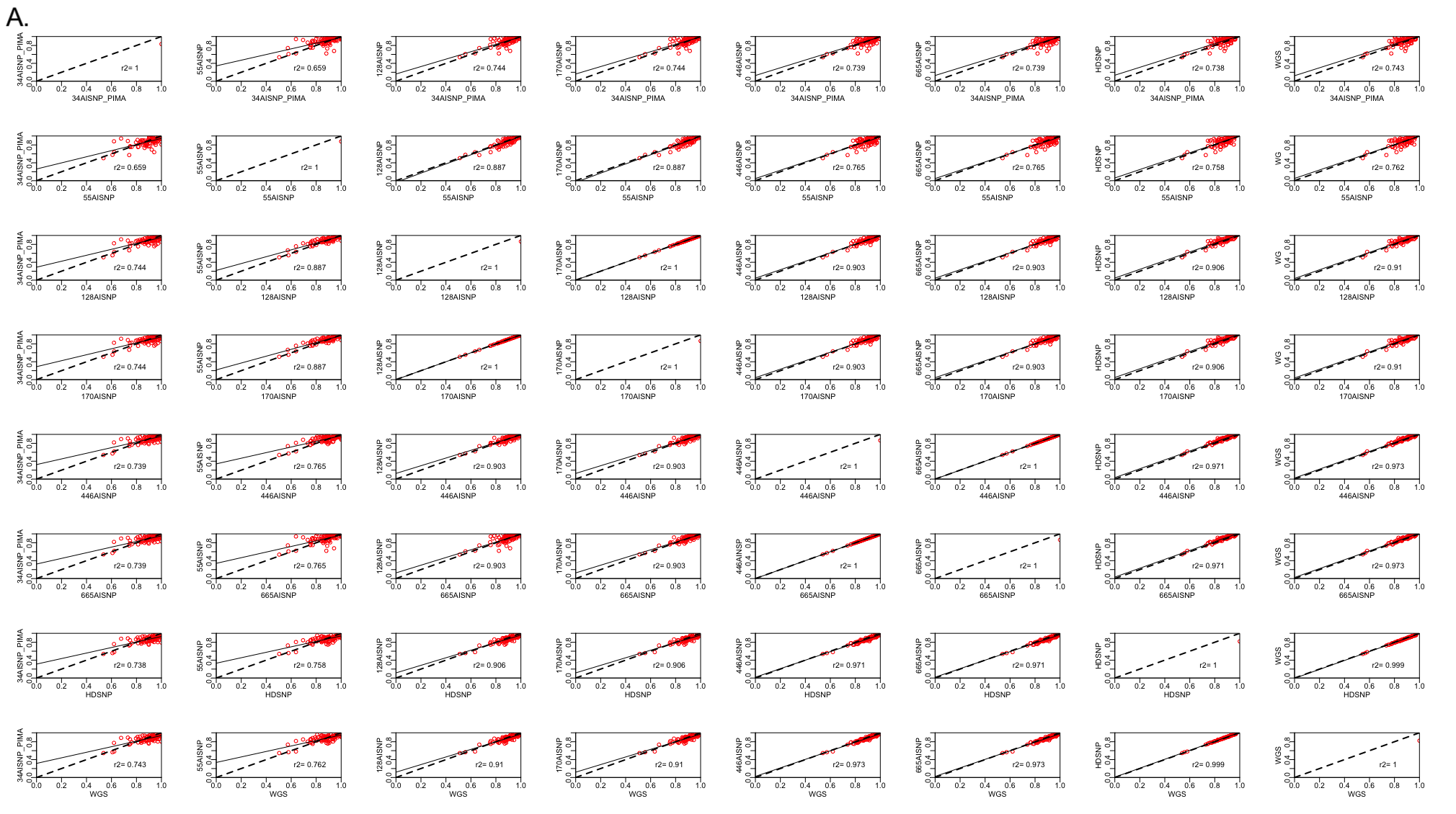


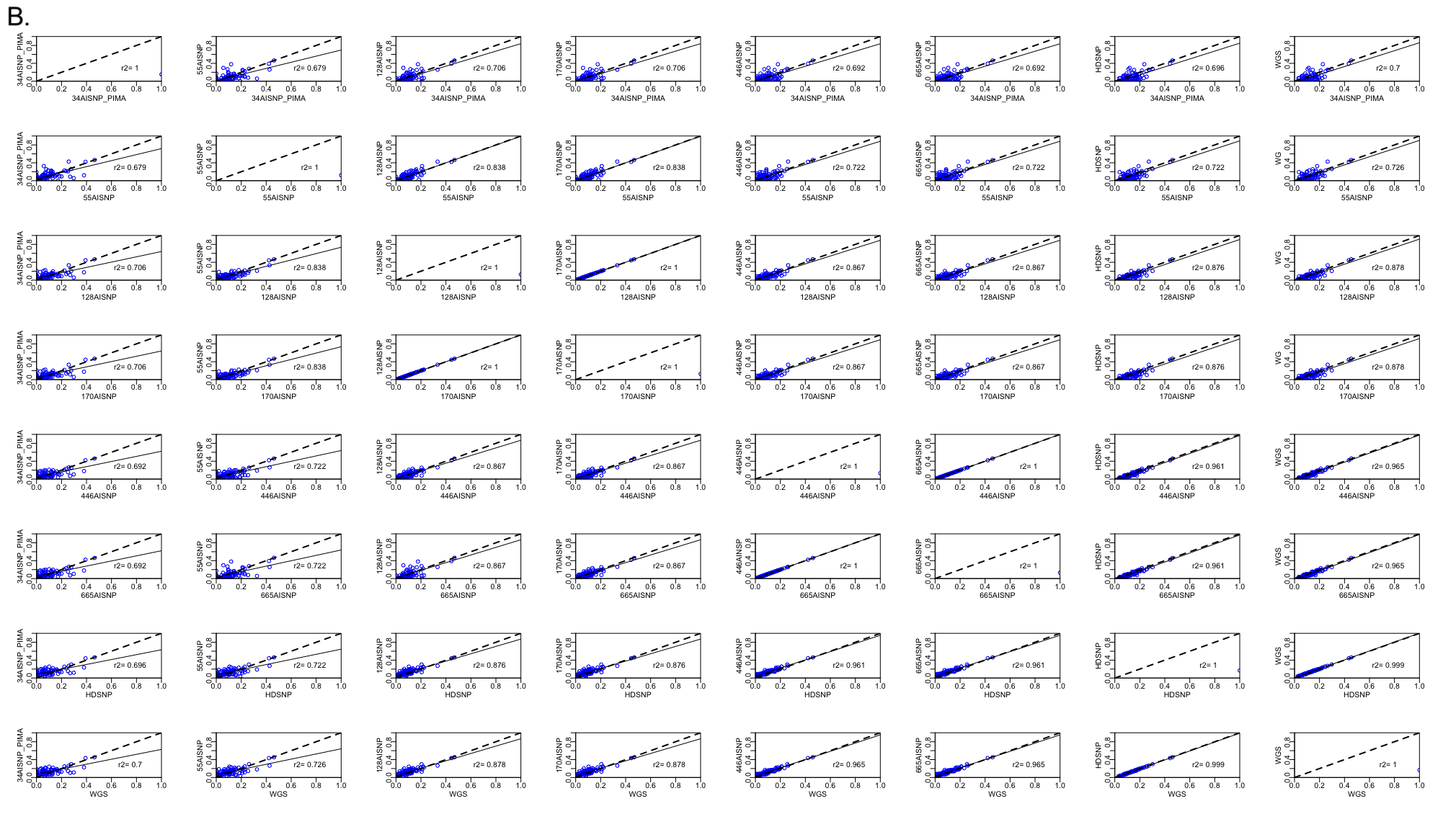


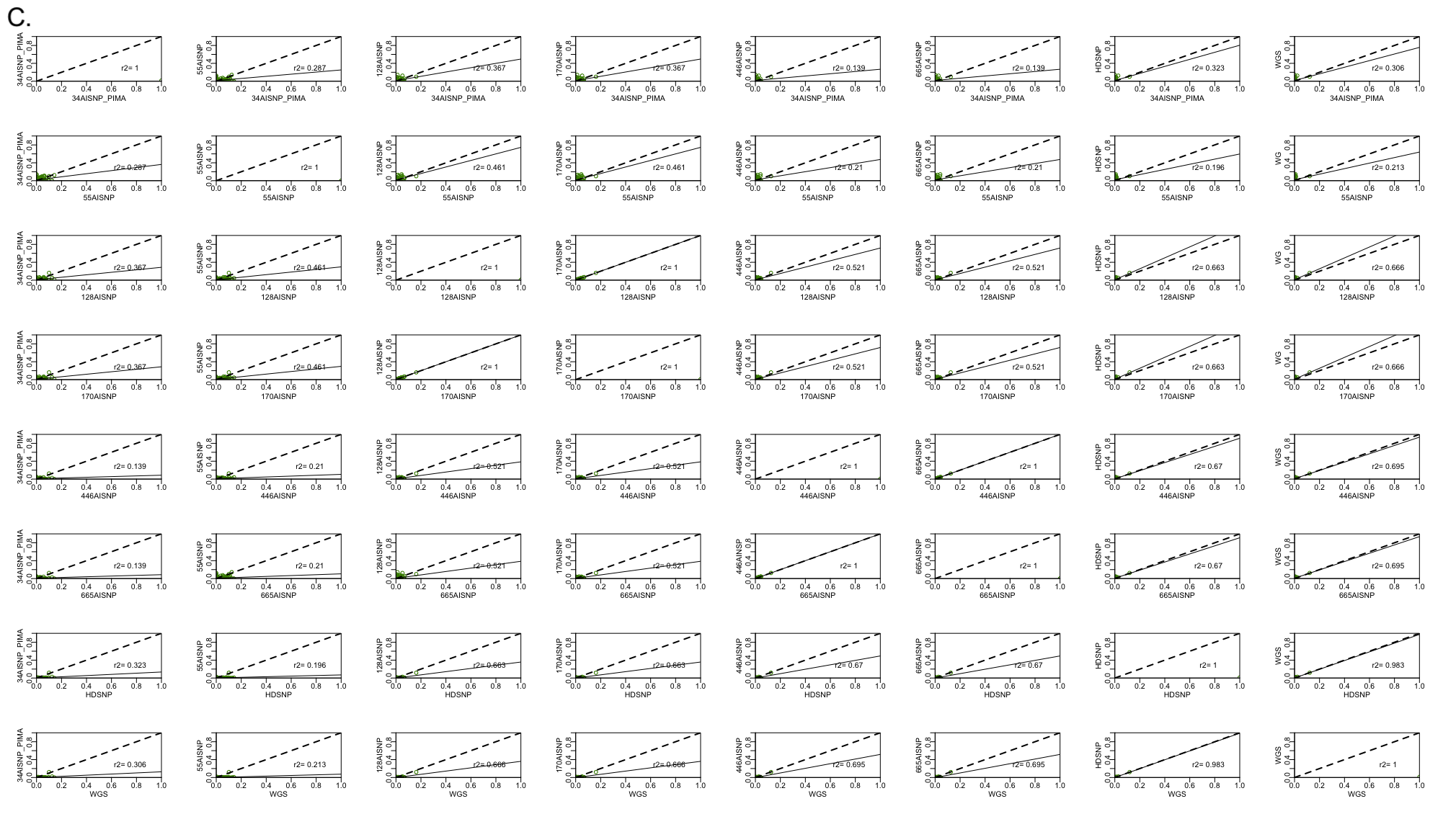


**Figure S11. Pairwise comparison of ancestry inferences for the African Caribbean samples with the 8 panel sets evaluated (34 AISNP +PIMA; 55 AISNP; 128 AISNP; 170 AISNP; 446 AISNP; 672 AISNP; HDSNP, WGS).** In the figure, r2 corresponds to the correlation coefficient, the black dashed line represents the trend, and the solid black line the perfect agreement between two panels. Components: (A) African ancestry (red), (B) European ancestry (blue) and (C) Native American ancestry (green).

**
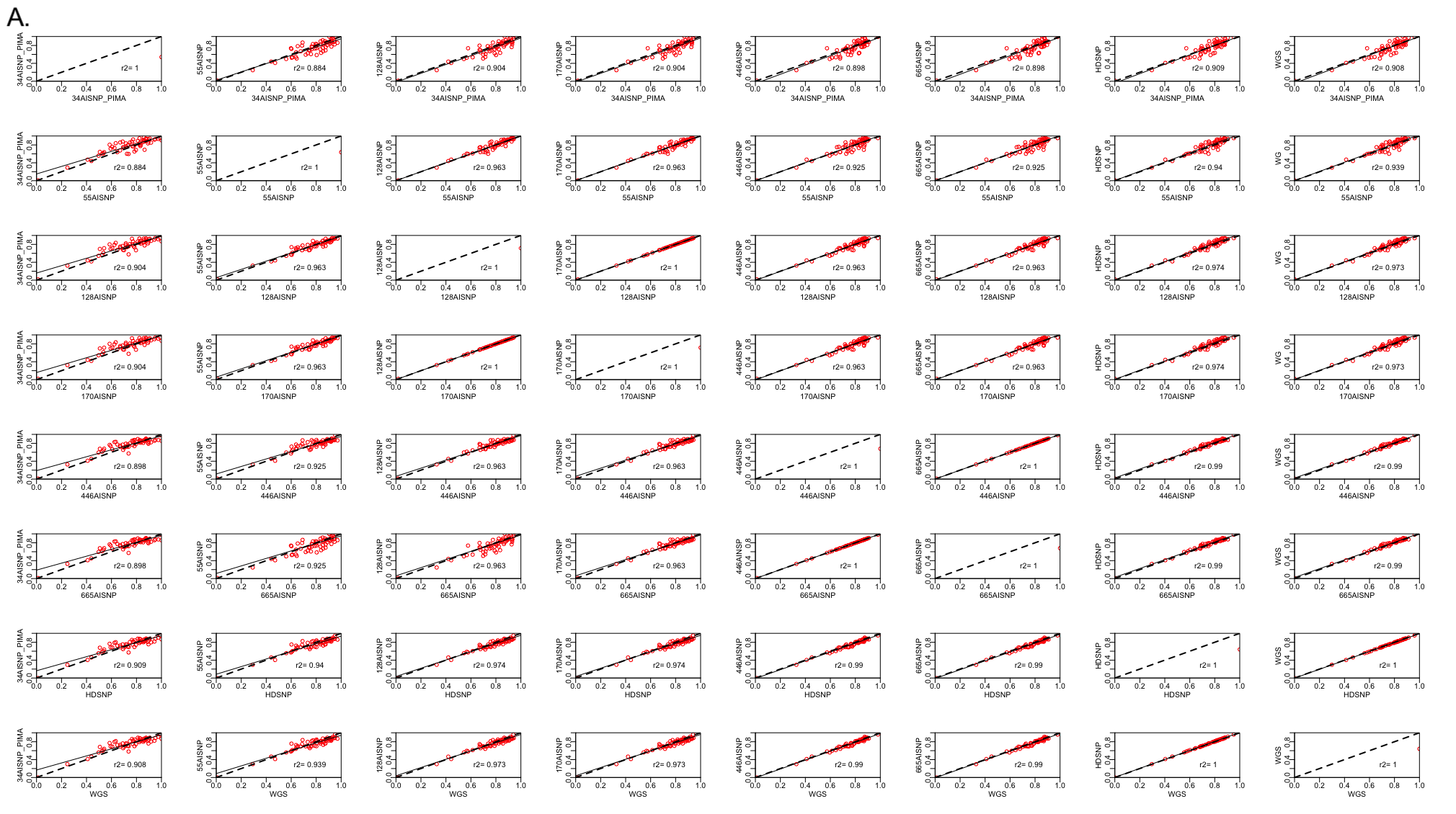
**

**
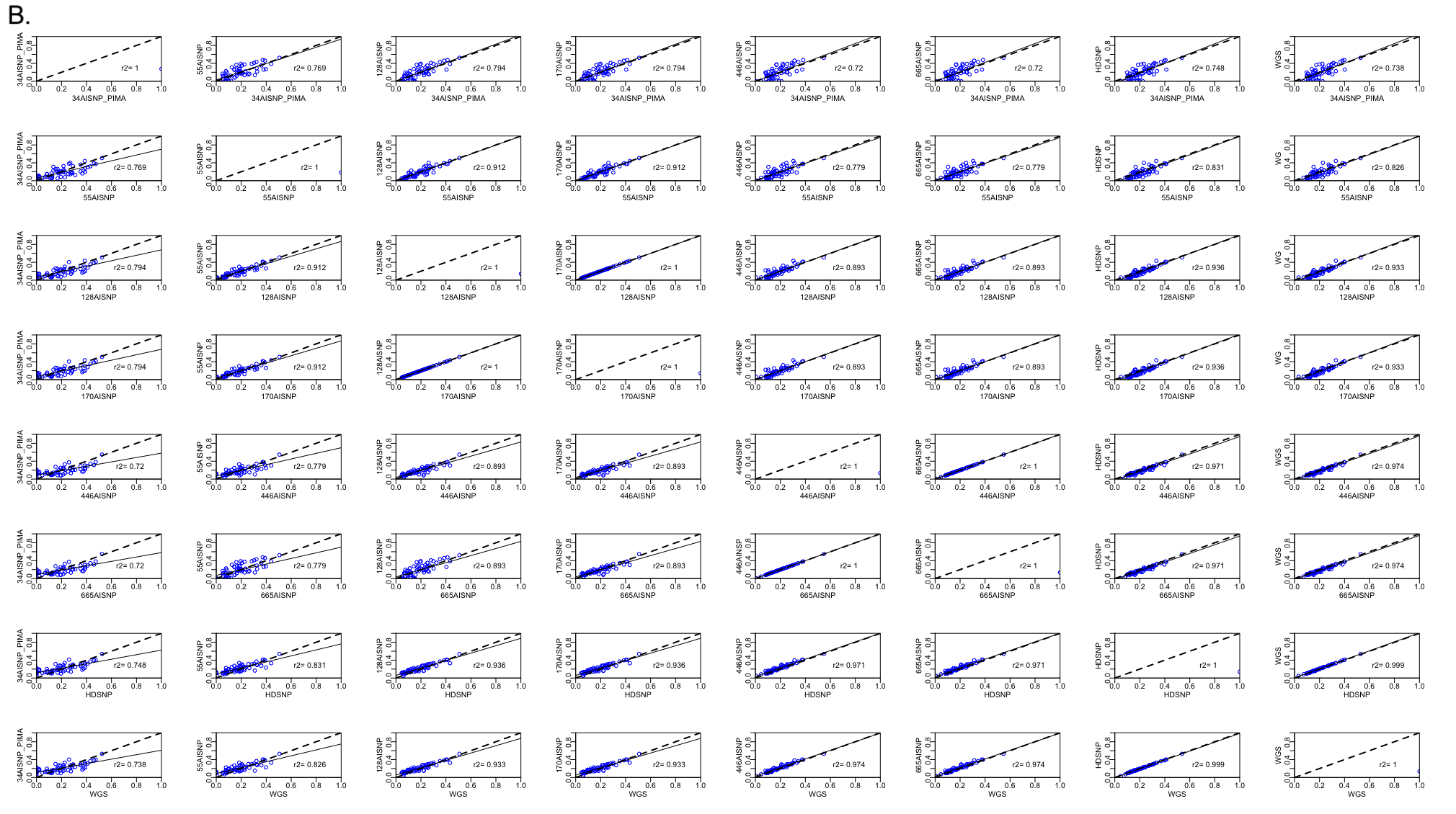
**

**
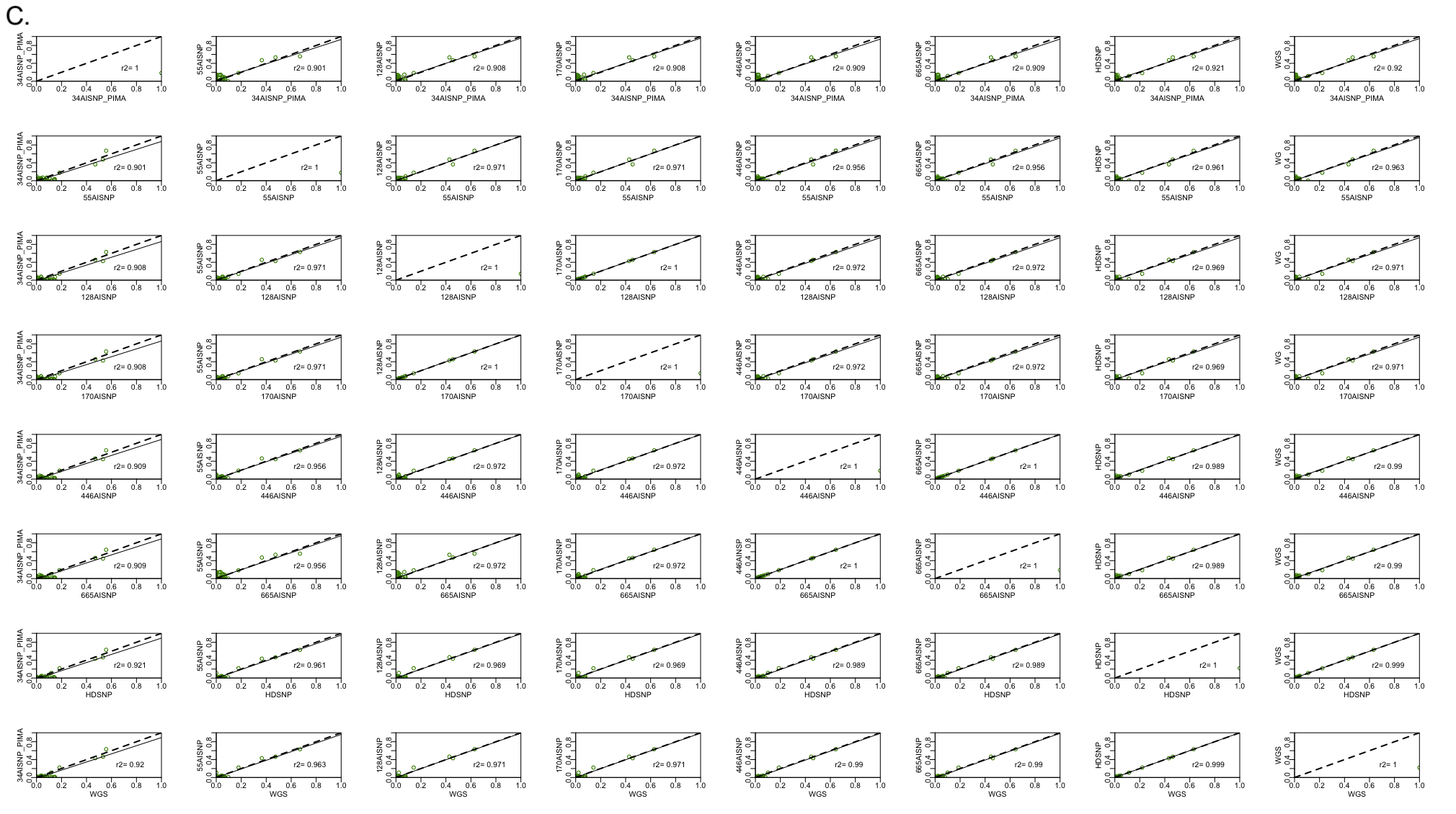
**

**Figure S12. Pairwise comparison of ancestry inferences for the African American samples with the 8 panel sets evaluated (34 AISNP +PIMA; 55 AISNP; 128 AISNP; 170 AISNP; 446 AISNP; 672 AISNP; HDSNP, WGS).** In the figure, r2 corresponds to the correlation coefficient, the black dashed line represents the trend, and the solid black line the perfect agreement between two panels. Components: (A) African ancestry (red), (B) European ancestry (blue) and (C) Native American ancestry (green).


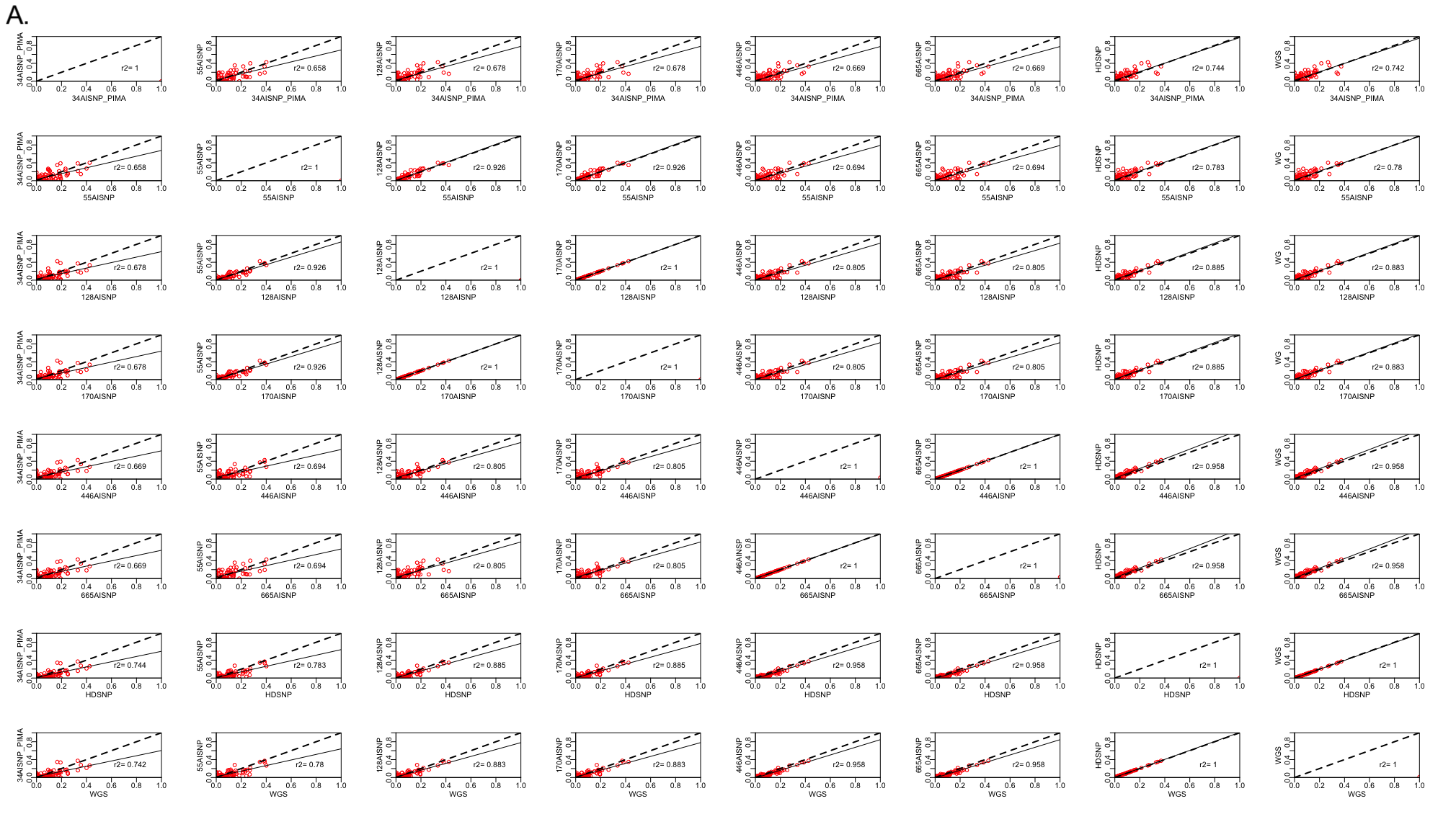


**
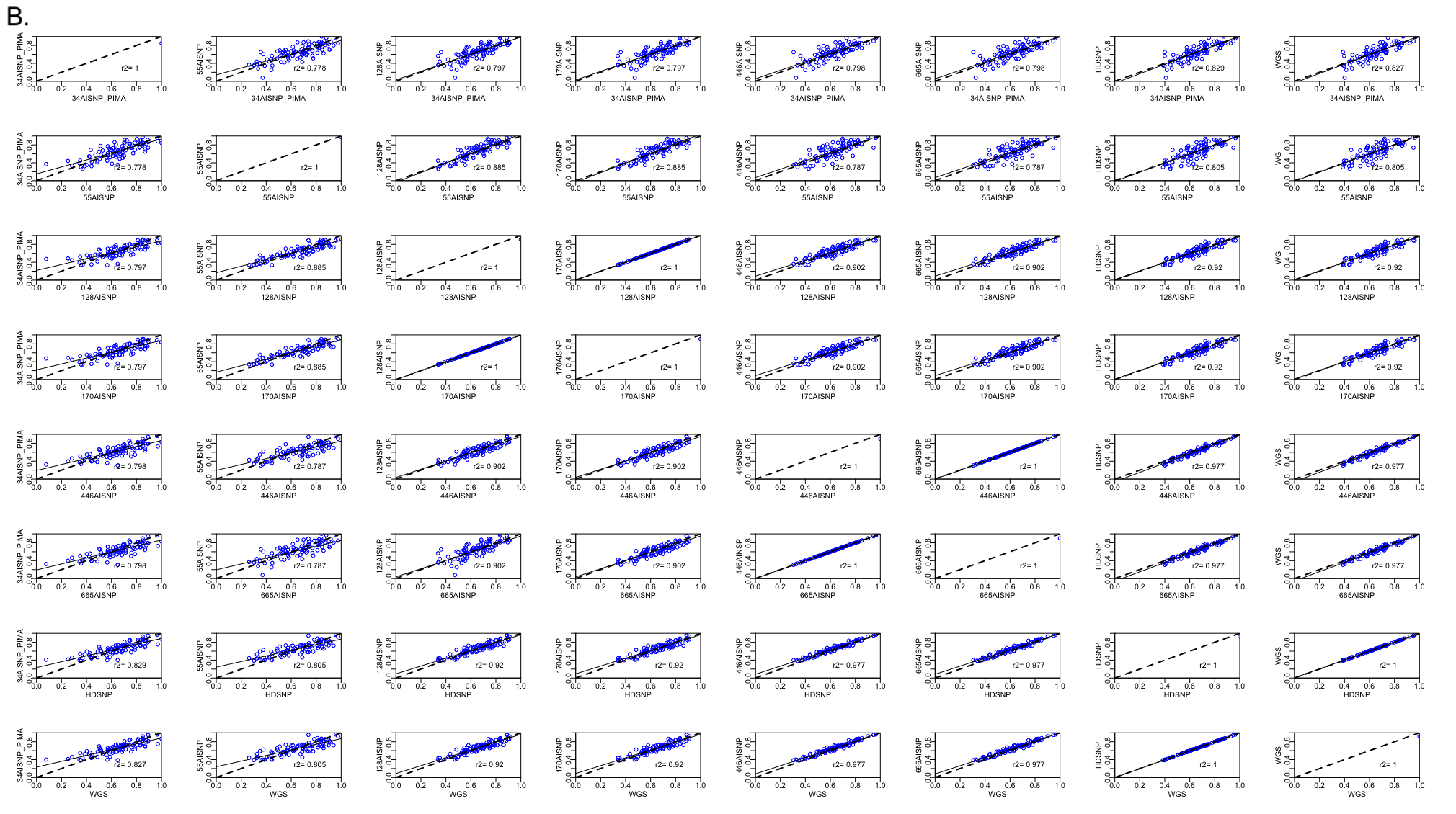
**

**
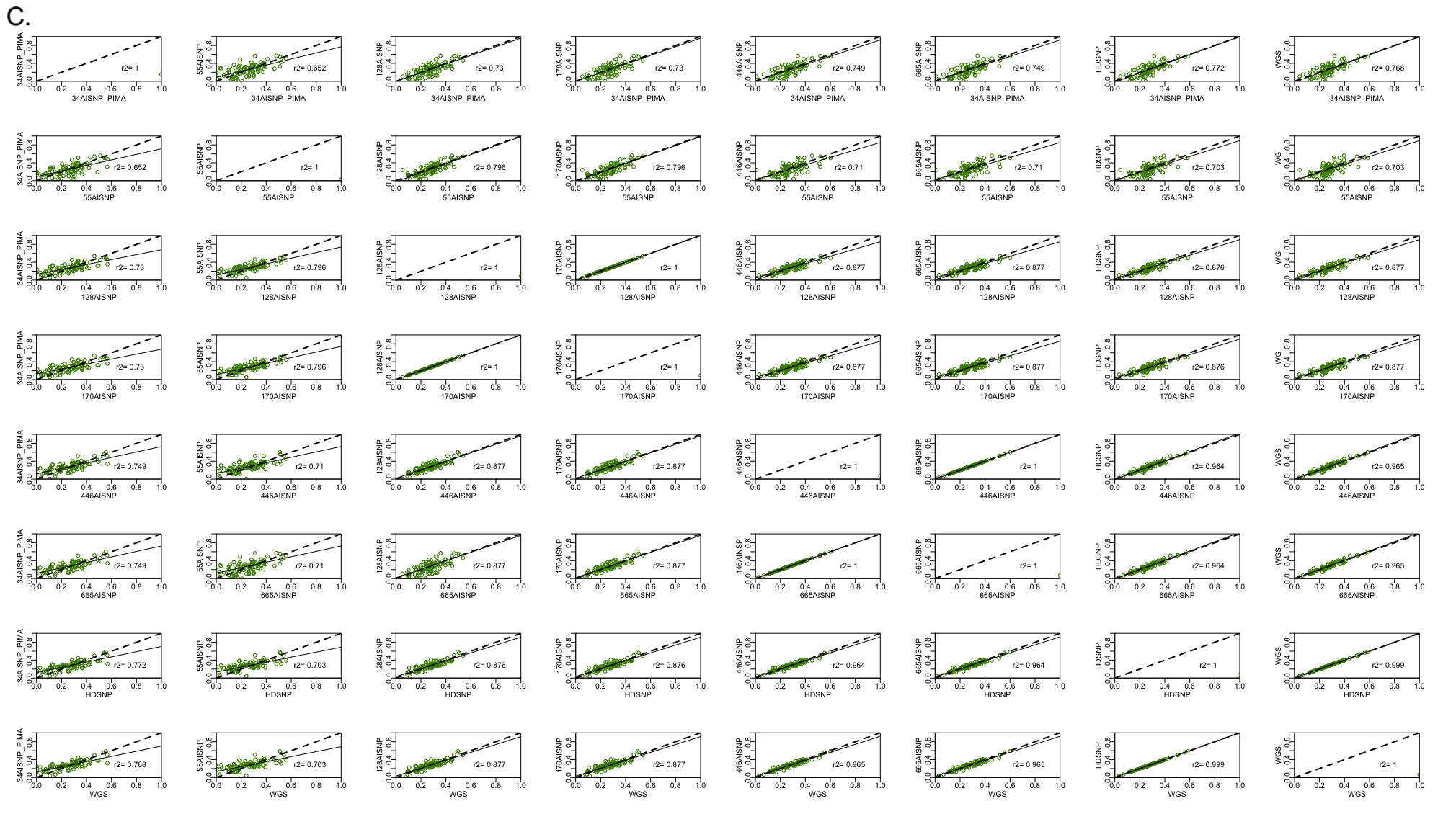
**

**Figure S13. Pairwise comparison of ancestry inferences for the Colombian samples with the 8 panel sets evaluated (34 AISNP +PIMA; 55 AISNP; 128 AISNP; 170 AISNP; 446 AISNP; 672 AISNP; HDSNP, WGS).** In the figure, r2 corresponds to the correlation coefficient, the black dashed line represents the trend, and the solid black line the perfect agreement between two panels. Components: (A) African ancestry (red), (B) European ancestry (blue) and (C) Native American ancestry (green).


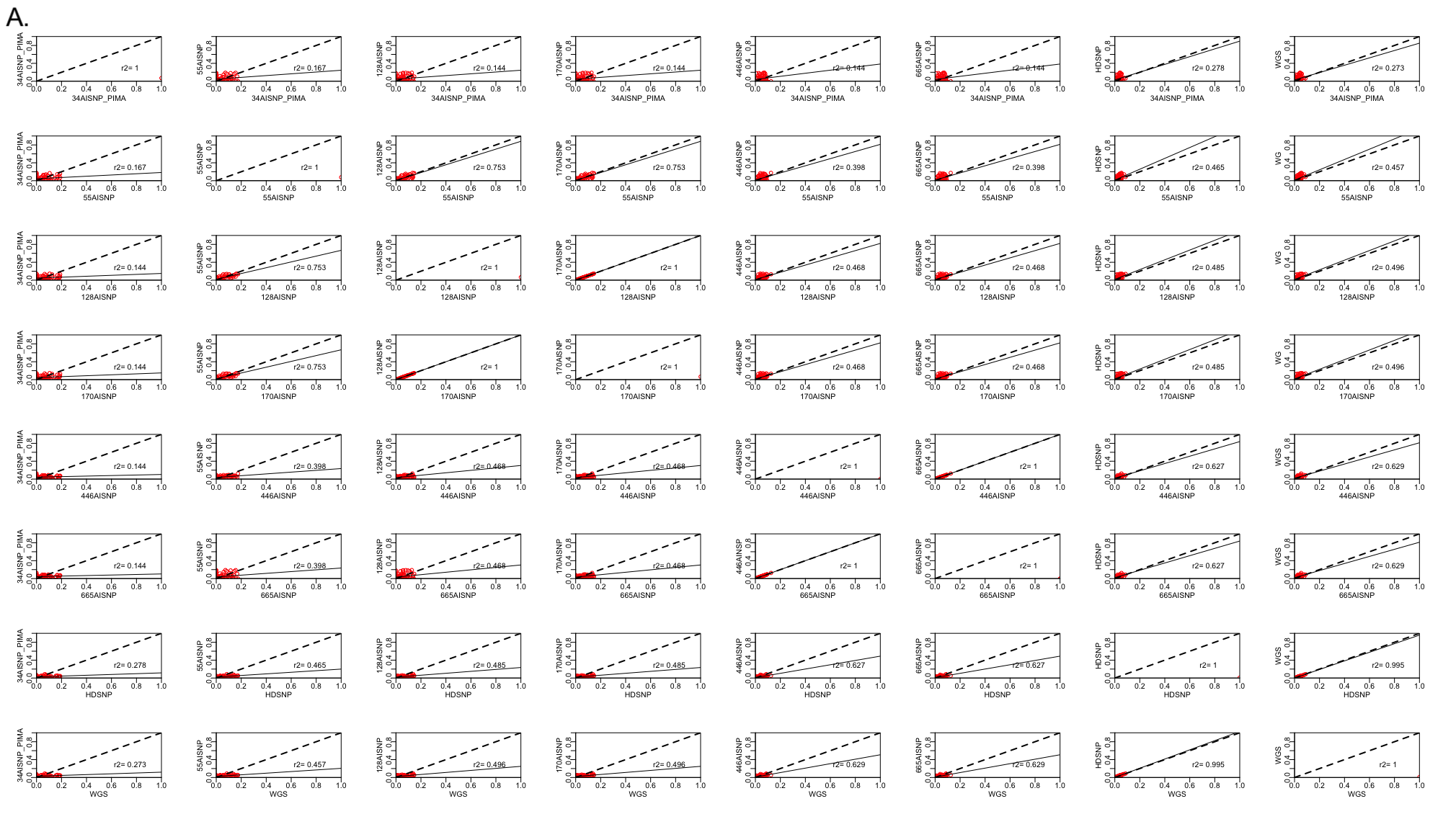


**
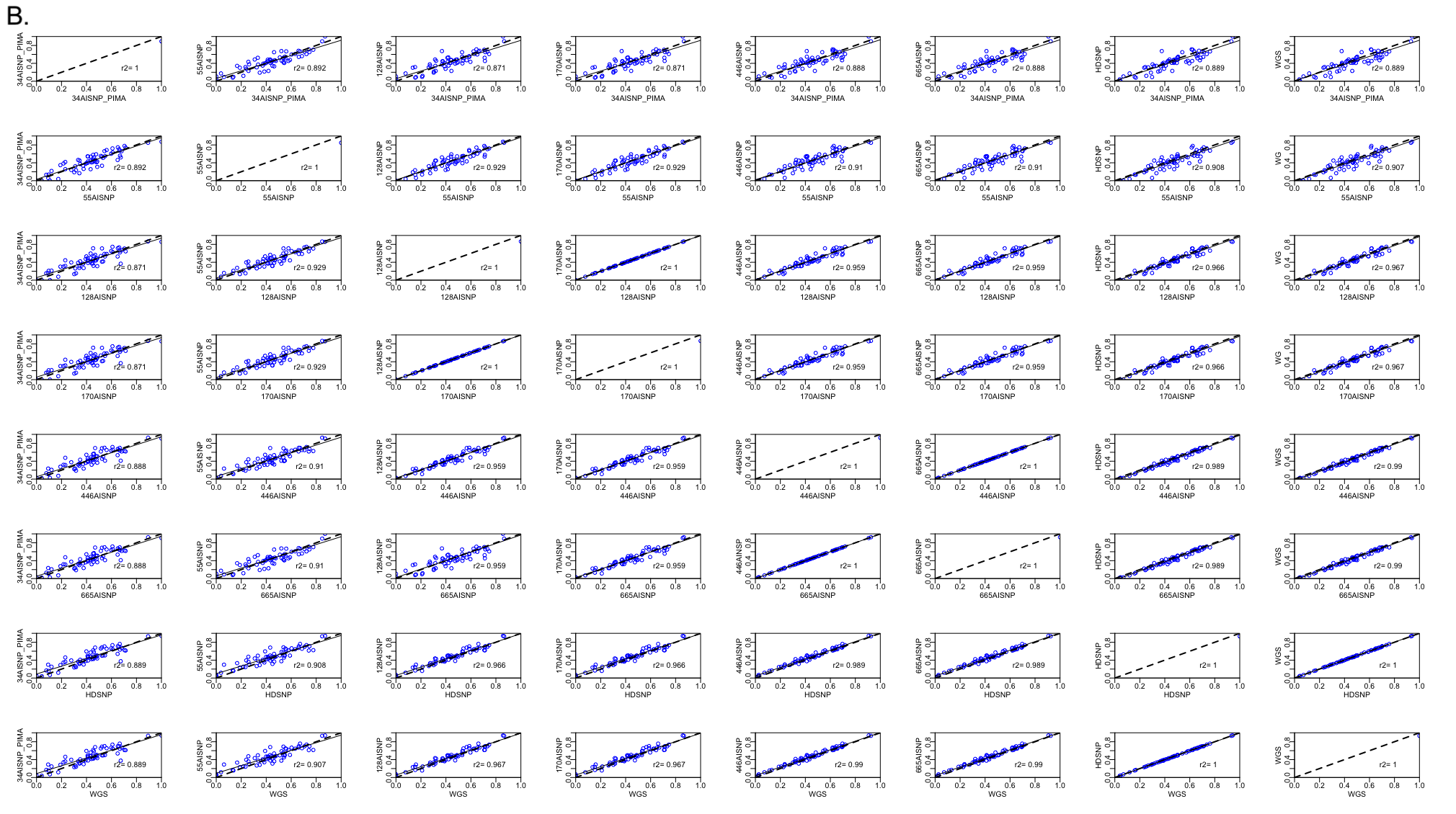
**

**
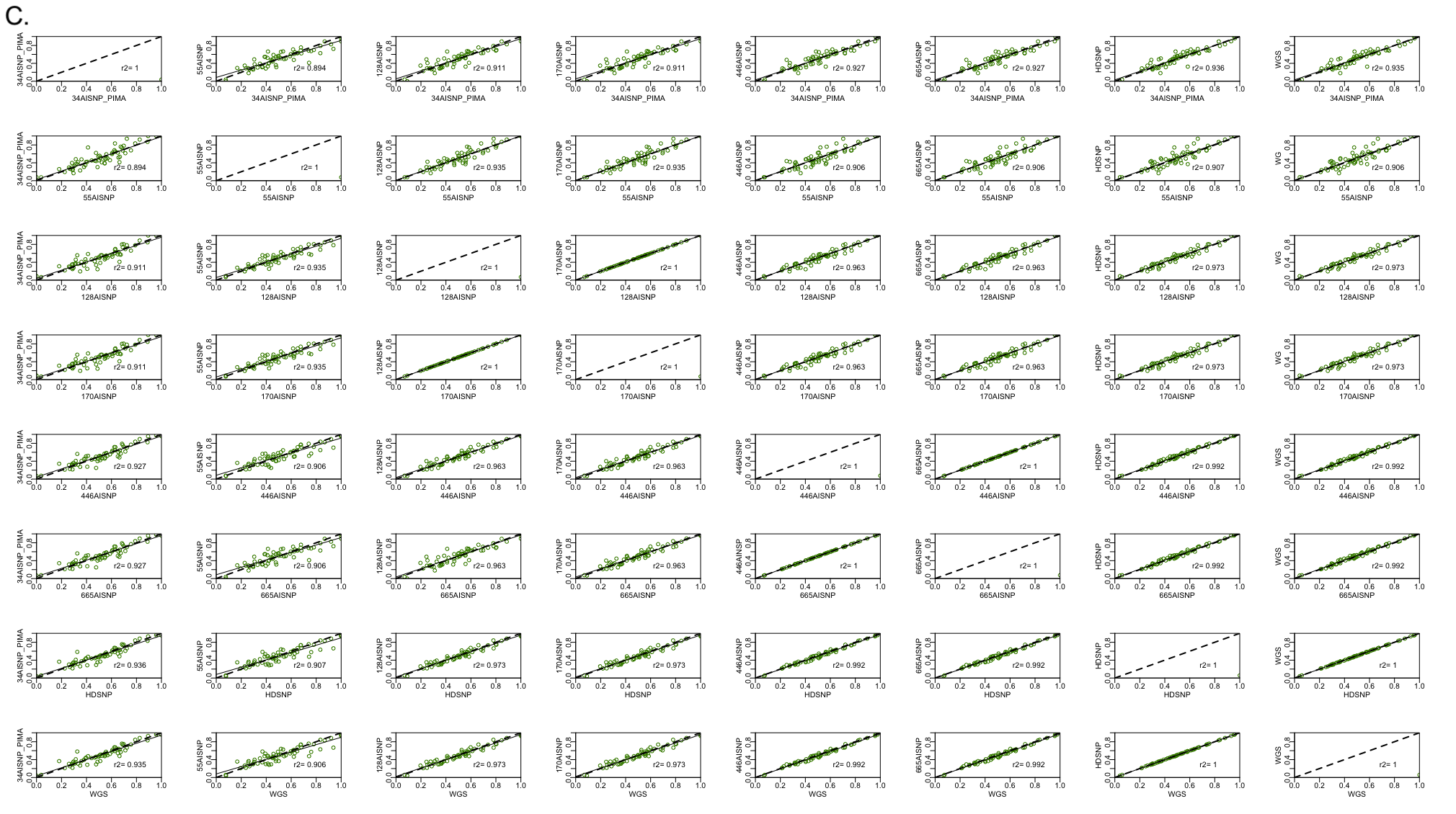
**

**Figure S14. Pairwise comparison of ancestry inferences for the Mexican samples with the 8 panel sets evaluated (34 AISNP +PIMA; 55 AISNP; 128 AISNP; 170 AISNP; 446 AISNP; 672 AISNP; HDSNP, WGS).** In the figure, r2 corresponds to the correlation coefficient, the black dashed line represents the trend, and the solid black line the perfect agreement between two panels. Components: (A) African ancestry (red), (B) European ancestry (blue) and (C) Native American ancestry (green).


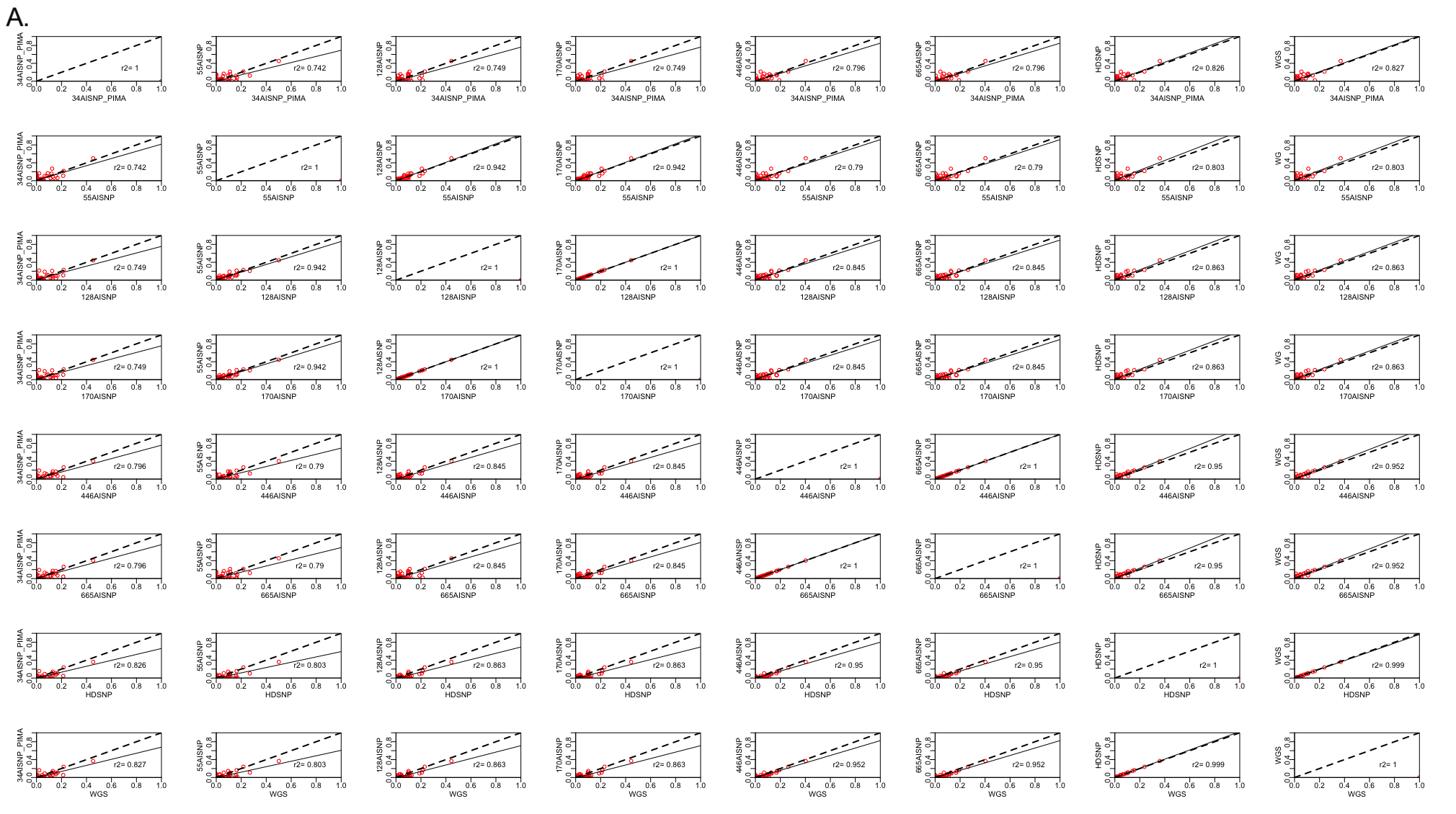


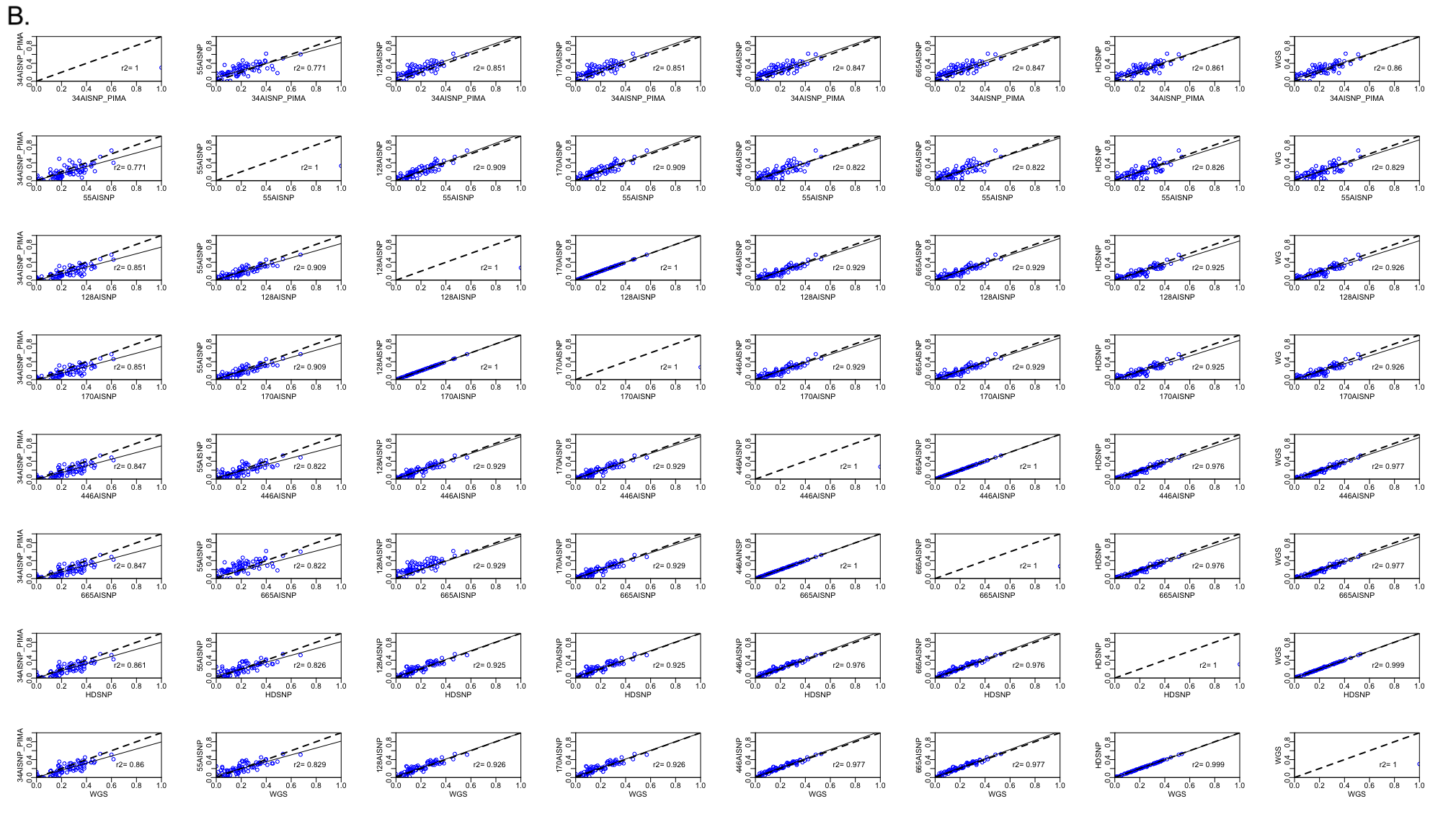


**
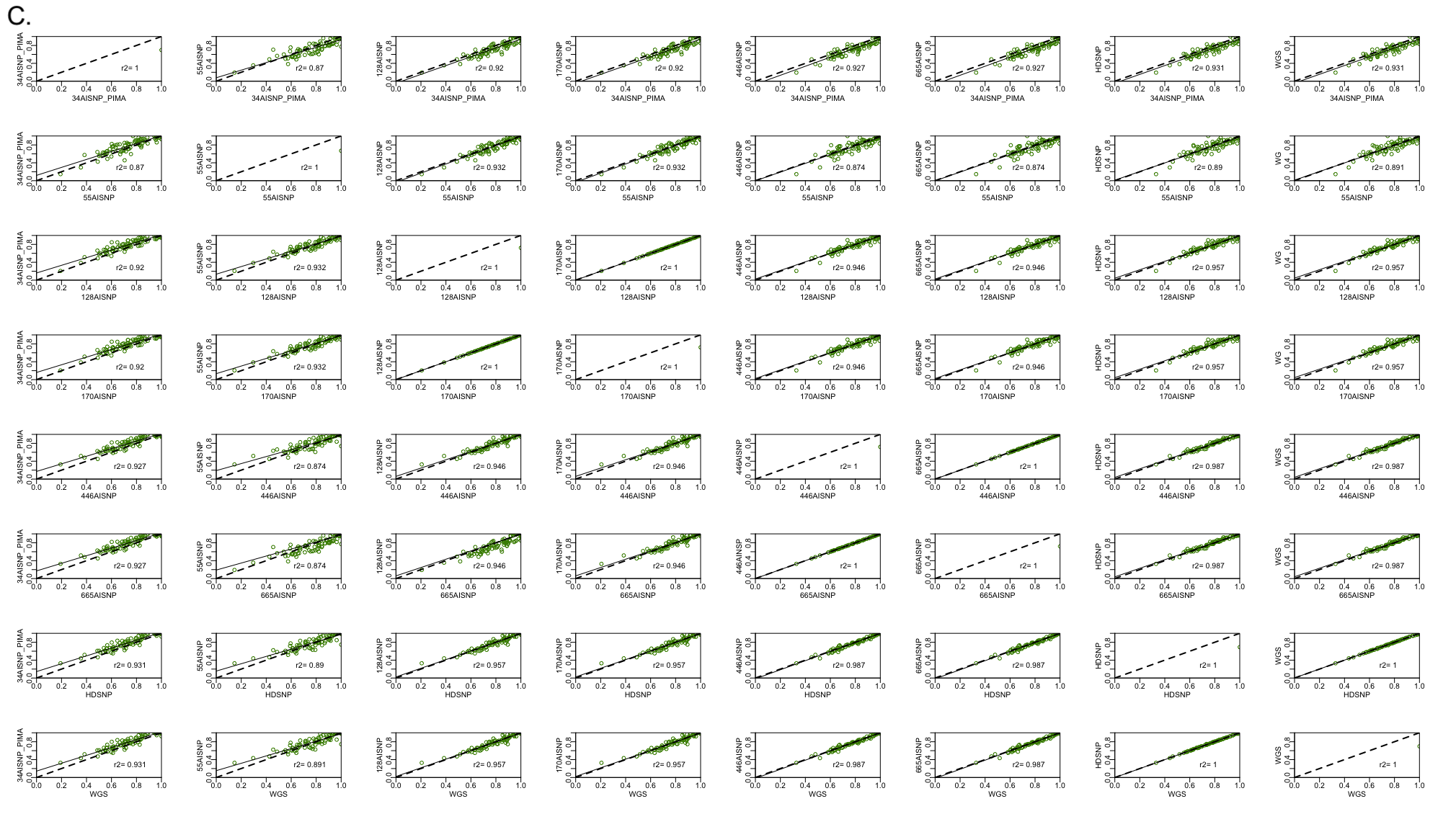
**

**Figure S15. Pairwise comparison of ancestry inferences for the Peruvian samples with the 8 panel sets evaluated (34 AISNP +PIMA; 55 AISNP; 128 AISNP; 170 AISNP; 446 AISNP; 672 AISNP; HDSNP, WGS).** In the figure, r2 corresponds to the correlation coefficient, the black dashed line represents the trend, and the solid black line the perfect agreement between two panels. Components: (A) African ancestry (red), (B) European ancestry (blue) and (C) Native American ancestry (green).


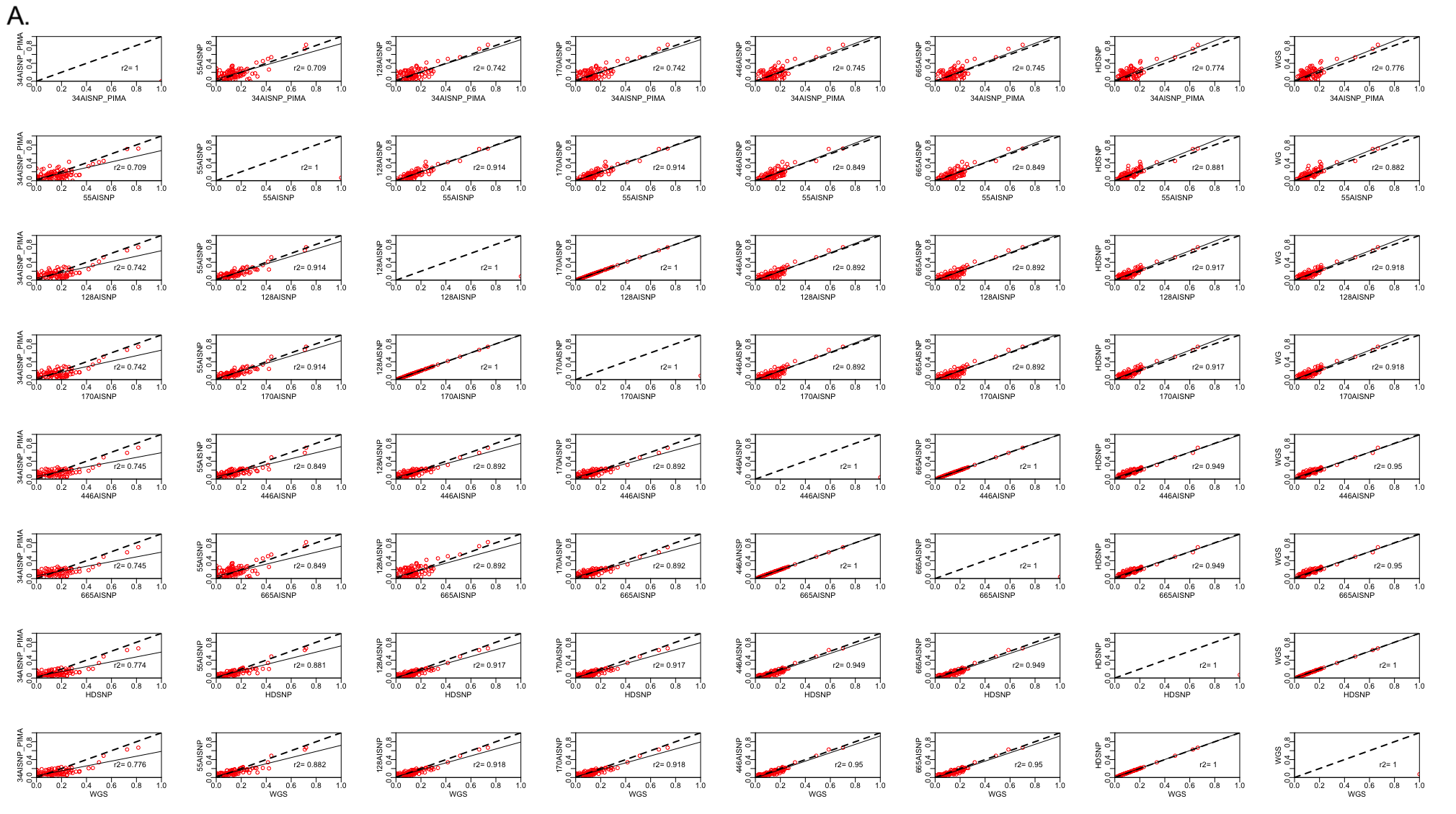


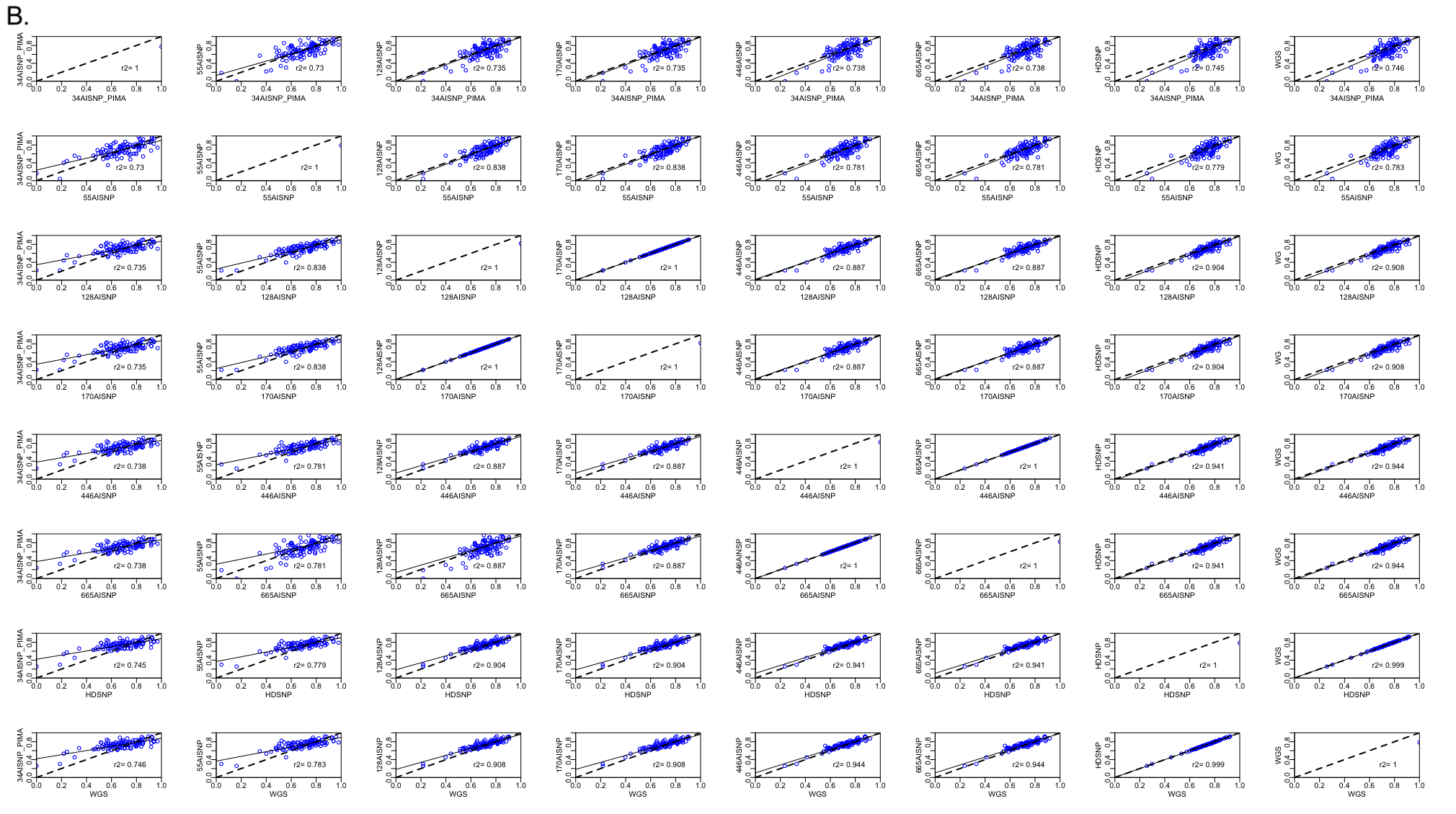


**
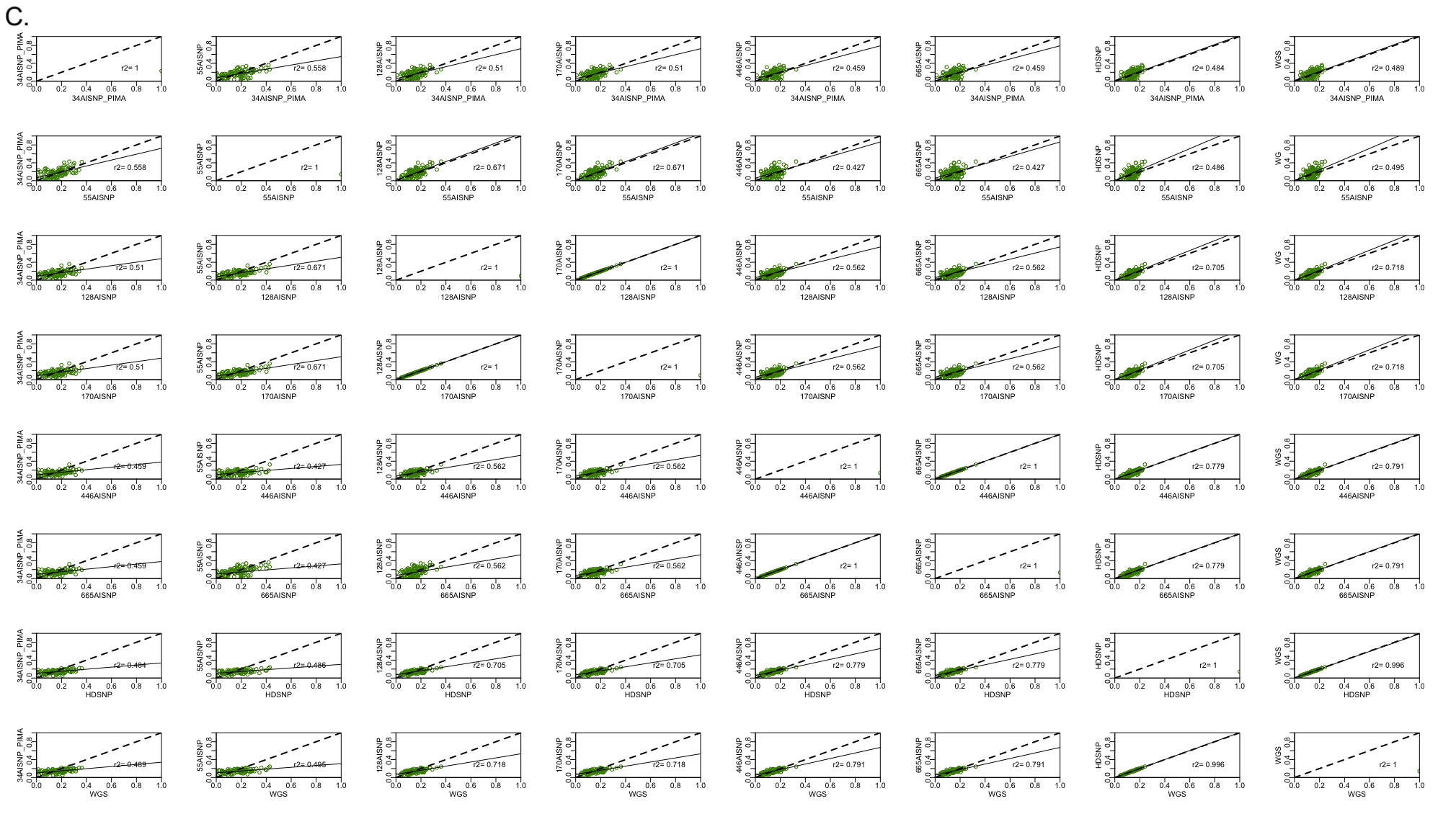
**

**Figure S16. Pairwise comparison of ancestry inferences for the Puerto Rican samples with the 8 panel sets evaluated (34 AISNP +PIMA; 55 AISNP; 128 AISNP; 170 AISNP; 446 AISNP; 672 AISNP; HDSNP, WGS).** In the figure, r2 corresponds to the correlation coefficient, the black dashed line represents the trend, and the solid black line the perfect agreement between two panels. Components: (A) African ancestry (red), (B) European ancestry (blue) and (C) Native American ancestry (green).

**
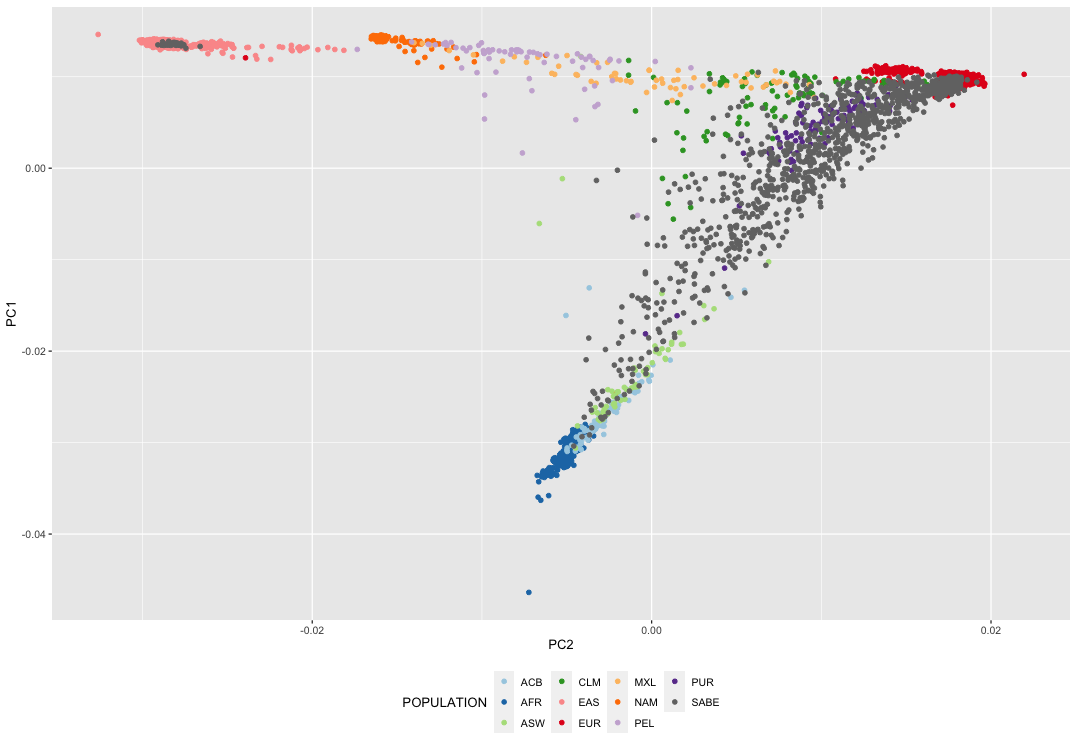
**

**Figure S1**. Principal Component Analysis (PCA) based on analysis of whole genome sequence data from the HGDP, 1KGP and SABE datasets (total of 3729 samples). The axes of the plot show the first and second principal components, for African (AFR), European (EUR), East Asian (EAS), Native American (NAM) samples and admixed population (Afro-American - ASW; Afro-Caribbean - ACB; Colombian - CLM; Mexican - MXL; Peruvian - PEL; Puerto Rican - PUR and Brazilian - SABE samples).
